# *DNMT3A* mutants provide proliferating advantage with augmentation of self-renewal activity in the pathogenesis of AML in *KMT2A-*PTD-positive leukemic cells

**DOI:** 10.1038/s41389-020-0191-6

**Published:** 2020-02-03

**Authors:** Rabindranath Bera, Ming-Chun Chiu, Ying-Jung Huang, Gang Huang, Yun-Shien Lee, Lee-Yung Shih

**Affiliations:** 1 Division of Hematology-Oncology, Chang Gung Memorial Hospital at Linkou, Taoyuan, Taiwan; 2 Divisions of Pathology and Experimental Hematology and Cancer Biology, Cincinnati Children’s Hospital Medical Center, 3333 Burnet Avenue, Cincinnati, OH 45229 USA; 3 School of Health Technologies, Ming Chuan University, Taipei, Taiwan; 4 Chang Gung University, Taoyuan, Taiwan

**Keywords:** Molecular biology, Cancer

## Abstract

Acute myeloid leukemia (AML) with partial tandem duplication of *histone-lysine N-methyltransferase 2A* (*KMT2A*-PTD) is a subtype of AML and is associated with adverse survival, yet the molecular pathogenesis of *KMT2A*-PTD is not fully understood. DNA methyltransferase 3A (*DNMT3A*) is mutated in various myeloid neoplasms including AML, especially at the Arg882. Recently, it has been found that *DNMT3A* mutations frequently coexisted with *KMT2A*-PTD and are associated with inferior outcomes. We aimed to understand the biological role of DNMT3A mutation in *KMT2A*-PTD-positive cells. Herein, we found that overexpression of *DNMT3A* mutants (MT) in *KMT2A*-PTD-positive EOL-1 cells augmented cell proliferation and clonogenicity. Serial colony replating assays indicated that *DNMT3A*-MT increased the self-renewal ability of *Kmt2a*-PTD-expressing mouse bone marrow cells with immature morphology. At 10 months post bone marrow transplantation, mice with the combined *Kmt2a-*PTD and *DNMT3A*-MT showed hepatosplenomegaly and leukocytosis with a shorter latency compared to control and *DNMT3A*-wild-type. Gene expression microarray analyses of bone marrow samples from human AML with *KMT2A*-PTD/*DNMT3A*-MT showed a stem cell signature and myeloid hematopoietic lineage with dysregulation of *HOXB* gene expression. In addition, human bone marrow AML cells carrying *KMT2A*-PTD/*DNMT3A*-MT showed abnormal growth and augmented self-renewal activity in primary cell culture. The present study provides information underlying the pathogenic role of DNMT3A-MT with KMT2A-PTD in proliferating advantage with augmentation of self-renewal activity in human leukemia, which may help to better understand the disease and to design better therapy for AML patients with these mutations.

## Introduction

Acute myeloid leukemia (AML) is a heterogeneous malignant hematopoietic disorder, which is classified by distinct morphologies, cytogenetics and molecular subtypes^1^. The *histone-lysine N-methyltransferase 2A* (*KMT2A*) gene, located on chromosome (11q23), has been found in more than 100 different fusion partners^2^. The SET domain of KMT2A has histone methyltransferase activity that specifically methylates lysine 4 on histone H3 (H3K4), a modification typically associated with transcriptionally active regions of chromatin^3–5^. Apart from translocation the *KMT2A*-PTD is an in-frame duplication of *KMT2A* exons (e.g. e9/e3 and e11/e3) in a 5′ to 3′ direction and produces a gain-of-function elongated protein^6–8^. Our previous study found that 6% of de novo AML patients had *KMT2A*-PTD mutation, which was associated with poor outcomes^9^. A *Kmt2a*^PTD/WT^ mice model was reported not to develop leukemia but characterized by a proliferative advantage, abnormal self-renewal capability, and blockage of myeloid differentiation in hematopoietic stem/progenitor cells^10,11^.

Because *KMT2A*-PTD alone does not generate leukemia, the acquisition of other cooperating mutations is required for leukemia transformation. Previously, using a large cohort of 98 de novo AML patients with *KMT2A*-PTD, we reported that 90.8% of patients had at least one additional gene mutation including *FLT3*-ITD (44.9%), *DNMT3A* (32.7%), *RUNX1* (23.5%) and *TET2* (18.4%) mutations^12^. We observed not only a high frequency of coexistence of *DNMT3A* mutations with *KMT2A*-PTD but also a poor outcome in patients carrying both mutations^12^. However, the biological functions of *DNMT3A* mutation in *KMT2A*-PTD-positive leukemia cells have not been studied before.

## Results

### Addition of DNMT3A-MT in KMT2A-PTD-positive EOL-1 cells augmented cell proliferation and clonogenicity

**Fig. 1:**
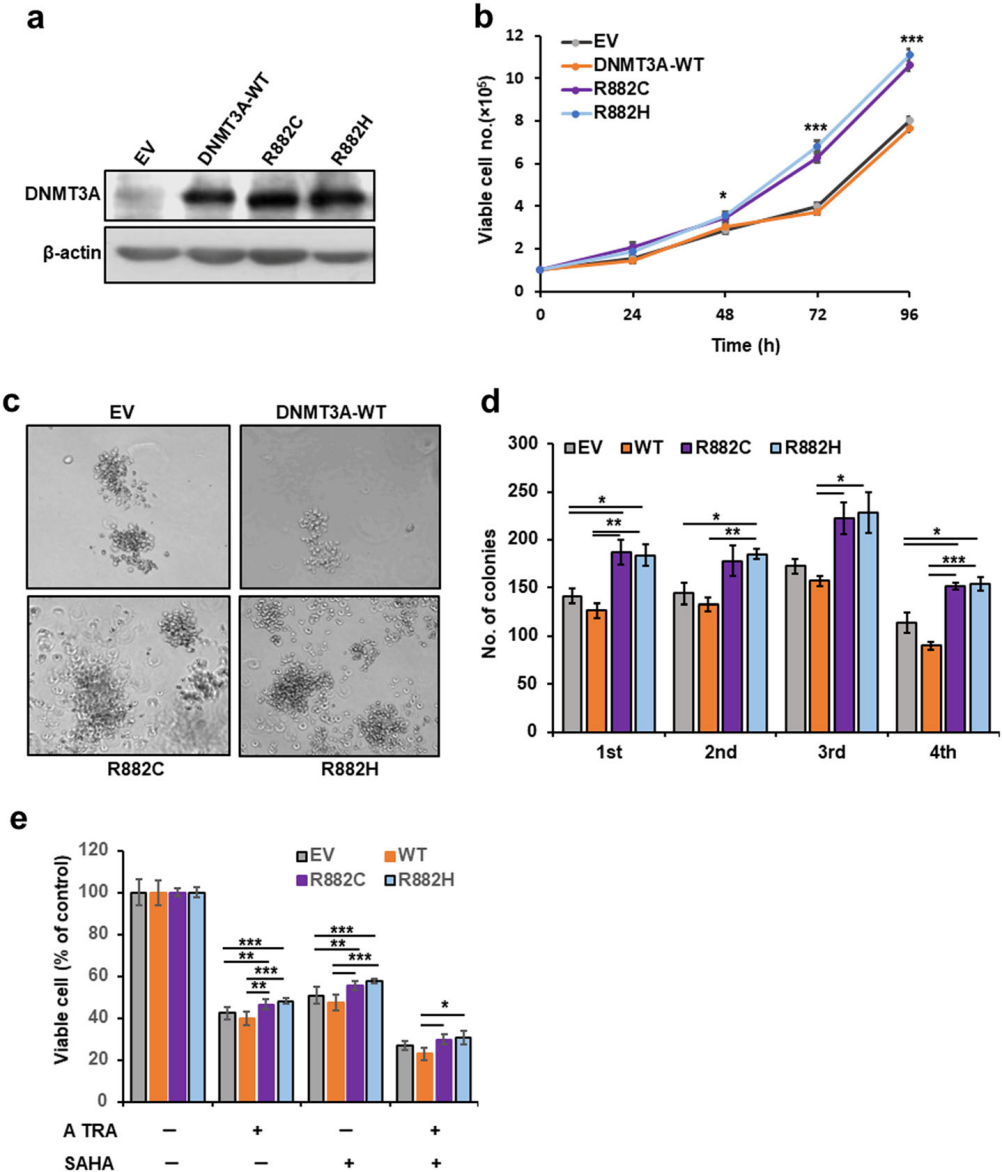
*DNMT3A*-MT deregulates cell proliferation and clonogenicity in EOL-1 cells. **a** Immunoblot of DNMT3A overexpressed with WT and mutant-*DNMT3A* in *KMT2A*-PTD positive EOL-1 cells. **b** Growth curves of EOL-1 cells stably transduced with WT- and mutant-*DNMT3A*. Representative results from three independent replicates are shown. **c**, **d** Colony-formation ability in methylcellulose containing medium after stable overexpression of WT; and mutant-*DNMT3A* in EOL-1 cells (original magnification: ×100). Colonies of more than 50 cells were scored on day 10 of cultures. **e** Cell viability of transformed EOL-1 cells in the presence of 200 nM ATRA, 600 nM SAHA and the combination of 100 nM ATRA with 500 nM SAHA at 72 h. Error bars represent ± s.d. of the mean of duplicate cultures and each experiment repeated at least three times. *P < 0.05, **P < 0.03, ***P < 0.01, either compared with the control or as indicated in figures. Two-sided Student’s *t* test was used to calculate the *P* value.

To elucidate the functional role of DNMT3A-WT/MT in KMT2A-PTD-positive leukemia cells, we stably expressed *DNMT3A*-wild-type (WT), *DNMT3A*-R882C/H (*DNMT3A*-MT) and empty vector (EV) control in EOL-1 cells (Fig. 1a). EOL-1 cell line was the reported *KMT2A*-PTD-positive human acute myeloid (eosinophilic) cells^13^. We assayed cell proliferation, clonogenicity and self-renewal activity of transduced cells. The results showed that cell proliferation, colony formation, and self-renewal activity were significantly higher in EOL-1 cells carrying *DNMT3A*-MT compared to WT or control (Fig. 1b−d). To check the drug sensitivity, EOL-1 cells were cultured in the presence or absence of various concentrations of all-trans retinoic acid (ATRA) and suberanilohydroxamic acid (SAHA) for 3 days (Supplemental Fig. S1a). To understand the potency of ATRA and SAHA in endogenously expressed *KMT2A*-WT cells, we used U937 cells (Supplemental Fig. S1b). U937 cell line was one of the myeloid cell lines that expressed WT-*KMT2A*. We found that cell viability of EOL-1 and U937 cells in the presence of ATRA or SAHA was dissimilar and dependent on different cell context manners. To test the sensitivity of ATRA and SAHA of transduced EOL-1 cells, we cultured *DNMT3A*-WT/MT-stably expressing cells in the presence of 200 nM ATRA, 600 nM SAHA and the combination of 100 nM ATRA with 500 nM SAHA for 3 days. All drugs effectively reduced cell proliferation at selective doses; reduction of cell proliferation of *DNMT3A*-MT-expressing cells was less than those of WT or control cells (Fig. 1e). Moreover, it was found that the combination of ATRA and SAHA was more effective compared to the single drug (Fig. 1e). To assess the role of *DNMT3A* mutants in the differentiation of *KMT2A*-PTD-positive EOL-1 cells, we incubated transduced EOL-1 cells with ATRA, Na-byturate, and SAHA. Transduced EOL-1 cells treated with ATRA or Na-butyrate induced morphological change with modest differentiation; however, very little effect was observed in EOL-1 cells treated with 500 nM SAHA (Supplemental Fig. S2a, b). With the treatment of 100 nM ATRA, Liu’s reagents stained smears showed a similar morphological feature of transformed EOL-1 cells (Supplemental Fig. S2c). However, EOL-1 cells transduced with *DNMT3A*-R882H mutant had a lower number but not significantly different expression of CD11b positive cells (Supplemental Fig. S2d). Flow cytometry data showed that CD11b expression was decreased in EOL-1 cells transduced with *DNMT3A*-MT compared to WT or EV, although there were no major morphological differences after 500 μM Na-butyrate treatment on transduced EOL-1 cells (Supplemental Fig. S2e). In contrast, transformed EOL-1 control cells treated with DMSO alone had no effect on CD11b expression (Supplemental Fig. S2f).

### Primary human KMT2A-PTD/DNMT3A mutants bone marrow cell (BMC) exhibited hyperproliferation, clonogenicity and self-renewal activity

**Fig. 2:**
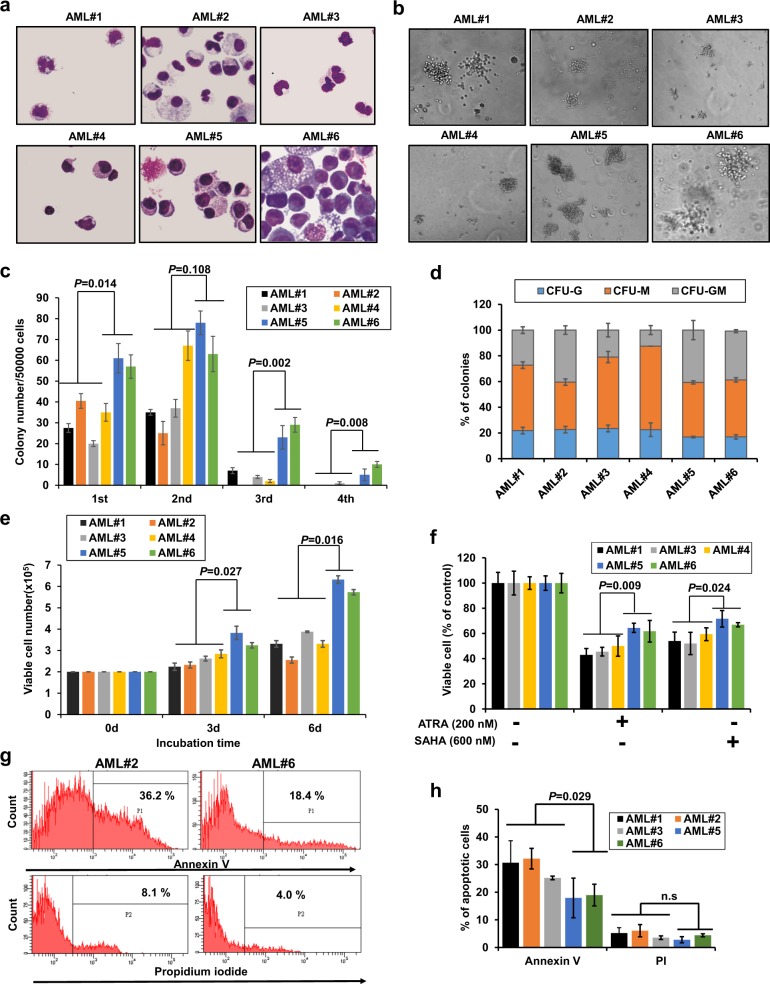
Primary human KMT2A-PTD/DNMT3A mutants BM cells exhibited hyperproliferation, clonogenicity and self-renewal activity. **a** Representative cell morphology with Liu’s reagents stained smears (original magnification: ×400) of primary human *KMT2A*-PTD AML cells with *DNMT3A*-WT/MT. *DNMT3A*-WT: AML#1, AML#2, AML#3, AML#4; *DNMT3A*-MT: AML#5, AML#6. **b**, **c** Colony-forming potentials and self-renewal activity of *KMT2A*-PTD/*DNMT3A*-WT/MT AML bone marrow cells in human MethoCult H4535 medium-enriched without erythropoietin (EPO), representative images of colonies in the second-round replating were shown. Colonies of more than 30 cells were scored using an inverted microscope on day 10 of cultures (**b**), original magnification: ×100. Columns represent the number of serially replated colonies (**c**). **d** Proportion of CFU-colonies of first-round plating. **e** Primary AML cell proliferation at day 3 (3d) and day 6 (6d) in a liquid culture medium containing 20% FBS and 20% conditional medium. **f** Cell viability of primary AML cells harboring *KMT2A*-PTD/*DNMT3A*-WT/MT in the presence of 200 nM ATRA, 600 nM SAHA incubated for 72 h. Each experiment repeated two times and error bars represent ±s.d. of duplicate cultures. **g** Representative flow cytometry data to determine the Annexin-V and propidium iodide (PI)-positive primary AML cells. **h** Primary BM cells were cultured in RPMI medium containing 20% FBS, 20% conditional medium with antibiotic for 3 days and the percentage of Annexin V and PI-positive cells were analyses by flow cytometry. Data are presented as means ± s.d. (*n* = 3). Two-sided Student’s *t* test was used to calculate the *P* value and compared between *KMT2A*/*DNMT3A*-WT and *KMT2A*/*DNMT3A*-MT groups, n.s. not significant.

Primary AML cells from four patients (AML#1, AML#2, AML#3 and AML#4) with *KMT2A*-PTD/*DNMT3A*-WT and two patients (AML#5 and AML#6) with *KMT2A*-PTD/*DNMT3A*-MT were cultured in six-well plate. The characteristics of six AML patients were presented in Supplemental Table S1. To explore the biological effect, we checked cell morphology, assayed cell proliferation, clonogenicity and self-renewal activity of primary human AML cells (Fig. 2a−e). Clonogenicity and self-renewal ability in methylcellulose medium were enhanced in primary AML cells with *KMT2A*-PTD/*DNMT3A*-MT compared with *KMT2A*-PTD/*DNMT3A*-WT cells (Fig. 2b, c). Results from the colony-forming unit (CFU) assays assessed the type of colonies, granulocyte (CFU-G), macrophage (CFU-M), and granulocyte with macrophage (CFU-GM) (Fig. 2d). Primary AML cell proliferation was significantly increased with more immature cells in *KMT2A*-PTD/*DNMT3A*-MT compared to *KMT2A*-PTD/*DNMT3A*-WT cells (Fig. 2a, e). Viability of primary AML cells was reduced in the presence of ATRA or SAHA; however, AML cells with *KMT2A*-PTD/*DNMT3A*-WT were more sensitive to those drugs when compared with *KMT2A*-PTD/*DNMT3A*-MT AML cells (Fig. 2f). Conversely, flow cytometry data showed that primary AML cells with *KMT2A*-PTD/*DNMT3A*-MT expressed lower Annexin-V and propidium iodide (PI)-positive cells indicating apoptosis reduction and more viable cells relative to *KMT2A*-PTD/*DNMT3A*-WT cells (Fig. 2g, h). It was noted that primary BM cells from one patient (AML#5) had expressed *FLT3*-ITD with *KMT2A*-PTD/*DNMT3A*-MT. Interestingly, in the context of cell proliferation, clonogenicity, and drug-induced cell death, there was no much difference between two human BMC harboring *KMT2A*-PTD/*DNMT3A*-MT with *FLT3*-ITD (AML#5) or without *FLT3*-ITD (AML#6).

### DNMT3A mutants deregulate hematopoietic stem cells (HSCs) activation, proliferation and RNA modifying-associated genes in primary AML cells with KMT2A-PTD

**Fig. 3:**
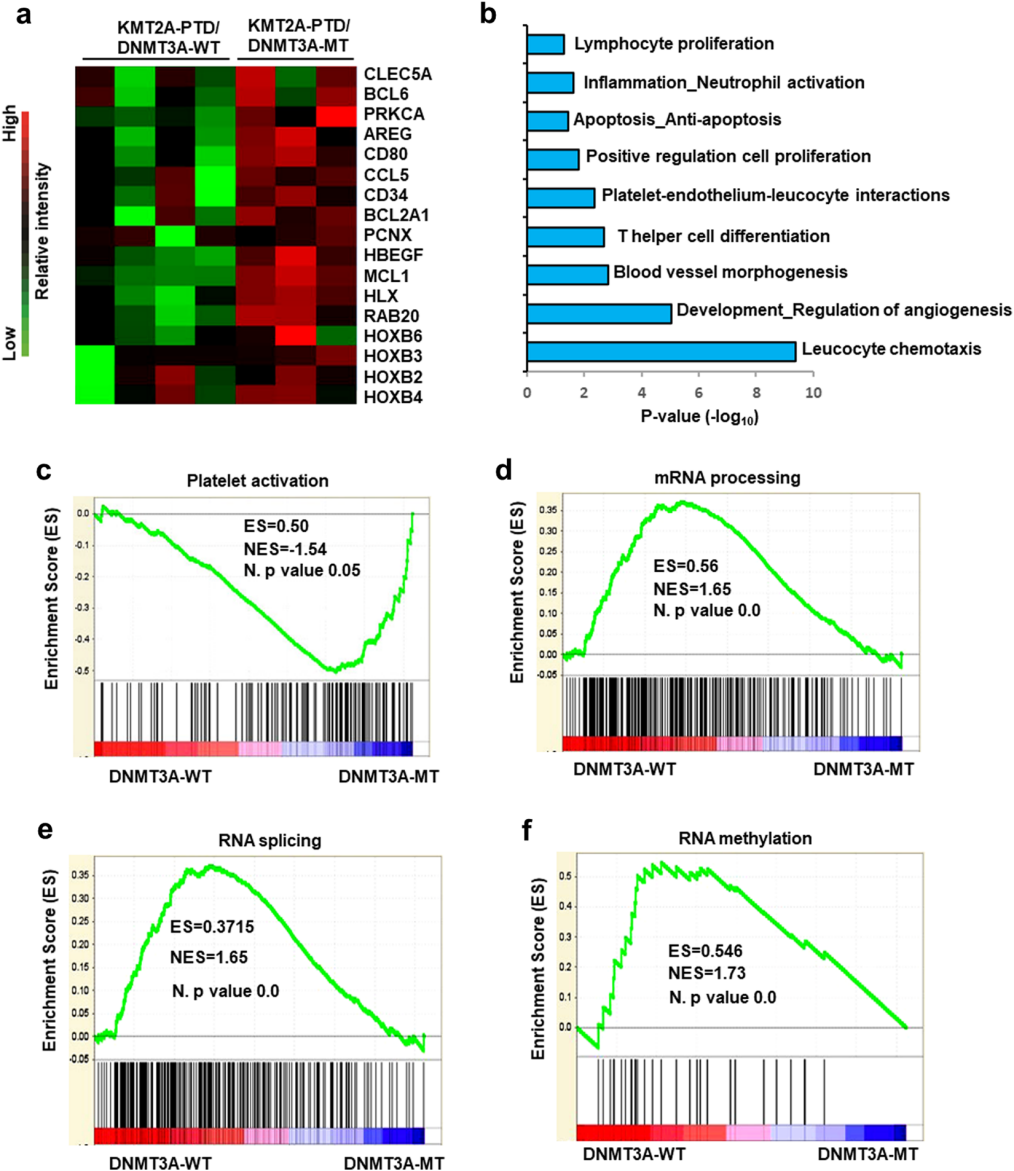
DNMT3A mutations deregulate HSC activation, proliferation, and RNA modifying-associated genes. **a** Heatmap representation of HSC activation, positive regulation of cell proliferation, *HOXB* gene expression identified as being differentially expressed in human primary AML cells harboring *KMT2A*-PTD with *DNMT3A*-WT and mutants. Red indicates upregulated genes compared to WT (green). **b** GO analyses of upregulated genes in *DNMT3A*-MT with *KMT2A*-PTD AML cells showing a series of genes enriched in different cellular and molecular processes including cell proliferation, angiogenesis, and regulation of apoptosis. **c**−**f** GSEA determining specific gene sets or pathways that are positively or negatively regulated by *DNMT3A* mutants with *KMT2A*-PTD. Compared with *KMT2A*-PTD/*DNMT3A*-MT AML (right side), *KMT2A*-PTD/*DNMT3A*-WT (left side) negatively correlated with gene sets downregulated in platelet activation (**c**), positively correlated with those of mRNA processing (**d**), RNA splicing (**e**) and RNA methylation (**f**). The enrichment scores (ES), normalized enrichment scores (NES) and *P* values were shown in figures.

To understand the mechanism underlying the biological effect of *DNMT3A* mutants in *KMT2A*-PTD-positive leukemia, we examined the patient samples harboring both *KMT2A*-PTD and *DNMT3A*-WT/MT by using global gene expression microarray analysis. Previously we had analyzed 25 AML samples of which 15 were *KMT2A*-PTD and 10 were *KMT2A*-translocation^14^. From these datasets, we analyzed seven samples, four *KMT2A*-PTD with *DNMT3A*-WT and three *KMT2A*-PTD with *DNMT3A*-R882 mutants (accession no. GSE15013). The characteristics of patients and disease subtypes are shown in Supplemental Table S2. Gene expression microarray data revealed that 762 and 209 genes were upregulated and downregulated, respectively, more than 2-folds in *KMT2A*-PTD AML with *DNMT3A* mutations compared to *DNMT3A*-WT samples (Supplemental Datasets S1, S2). We found that a group of genes associated with HSC activation, positive regulation of cell proliferation and myeloid differentiation was upregulated in *DNMT3A*-MT samples (Fig. 3a). *HOXB* genes were upregulated in *KMT2A*-PTD AML with *DNMT3A* mutations. Upregulated genes in *KMT2A*-PTD AML samples with *DNMT3A*-MT compared to *KMT2A*-PTD with *DNMT3A*-WT were used for Gene Ontology (GO) classification and enrichment analysis. Indeed, several upregulated gene sets were enriched in different cellular and molecular processes including lymphocyte proliferation, neutrophil activation, T-cell differentiation, regulation of angiogenesis (Fig. 3b). In addition, analysis based on the Hematopoietic Fingerprints Database^15^ revealed 83 genes were upregulated in *KMT2A*-PTD AML samples with *DNMT3A* mutation compared to *KMT2A*-PTD with *DNMT3A*-WT (Supplemental Table S3) samples. Gene set enrichment analyses (GSEA) of gene expression data revealed that the mutation of *DNMT3A* with *KMT2A*-PTD significantly altered the expression profile compared to *KMT2A*-PTD with *DNMT3A*-WT (Fig. 3c−f). GSEA indicated that the gene set induced by *DNMT3A*-WT with *KMT2A*-PTD positively correlated with those of enriched in mRNA processing, RNA splicing and RNA methylation activated gene sets, and negatively correlated with those enriched in platelet activation when compared with the gene sets induced by *DNMT3A* mutant with *KMT2A*-PTD AML (Fig. 3c−f).

### DNMT3A-MT upregulates HOXB gene expression in KMT2A-PTD-positive EOL-1 and primary AML cells

**Fig. 4:**
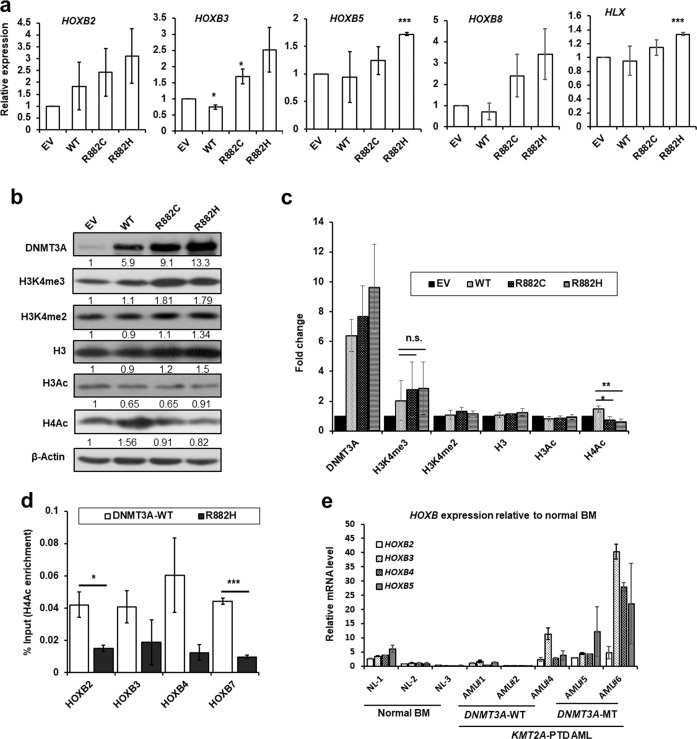
*DNMT3A*-MT deregulates *HOXB* gene expression in EOL-1 and primary AML cells. **a** *HOXB* expression in EOL-1 cells transduced with *DNMT3A*-WT/MT by quantitative RT-PCR analyses showing the same patterns observed in gene expression microarray analysis of patient samples with *KMT2A*-PTD/*DNMT3A*-WT and mutants. Data are expressed as mean ± s.d. of three independent experiments. *P < 0.05, ***P < 0.005 compared to EV. Two-sided Student’s *t* test was used to calculate the *P* value. **b** Immunoblot data showing H3K4me3 and H4Ac protein levels increased and decreased respectively in EOL-1 cells expressing DNMT3A-MT. β-Actin was used as a control for equal loading. **c** Quantitation of indicated proteins in transduced EOL-1 cells. Error bars presented as mean ± s.d. of three independent experiments. *P < 0.02, **P < 0.006; n.s. not significant. Two-sided Student’s *t* test was used to calculate the *P* value. **d** Levels of H4Ac at the promoters of *HOXB* genes in *DNMT3A-*WT and *DNMT3A*-R882H-expressing EOL-1 cells as detected by ChIP-qPCR. The relative amounts of immunoprecipitated DNA were depicted as a percentage of input DNA. Error bars presented as mean ± s.d. of two independent experiments. *P < 0.05, ***P < 0.005. Two-sided Student’s *t* test was used to calculate the *P* value. **e** Relative expression levels of *HOXB* genes were examined by quantitative RT-PCR in BM cells derived from normal control (*n* = 3) and from primary AML cells with *KMT2A*-PTD/*DNMT3A*-WT/MT. The values were normalized by GAPDH mRNA levels and expression was shown as relative to normal BM cells (average of 3). Experiment was repeated twice and data are expressed as mean ± s.d. of two experiments.

From gene expression microarray data analyses, we found that several genes including the *HOXB* cluster were upregulated in *KMT2A*-PTD AML with *DNMT3A* mutations compared to *DNMT3A*-WT. A portion of the differentially expressed genes was validated in EOL-1 cells stably expressed-*DNMT3A*-WT/MT (Supplemental Fig. S3a). Among them, antiapoptotic genes *BCL2A1* and *MCL1* that act as a key driver of survival in AML were also upregulated in mutant cells^16,17^. Moreover, we found that *HOXB* cluster genes including *HOXB2*, *HOXB3*, *HOXB5*, and *HOXB8* were upregulated in EOL-1 cells expressing *DNMT3A*-MT when compared with EV or *DNMT3A*-WT (Fig. 4a). However, expression of *HOXA* cluster genes including *HOXA5*, *HOXA7*, *HOXA9*, and *HOXA10* was not changed in mutant cells compared to either EV or WT cells (Supplemental Fig. S3b). Immunoblot data showed that EOL-1 cells transduced with *DNMT3A*-MT had increased but not a significantly different expression of H3K4me3 and significantly decreased expression of H4Ac compared to WT cells (Fig. 4b, c). We then asked whether *DNMT3A* mutation affected the status of H4 acetylation at the locus of *HOXB* cluster genes. ChIP assays were performed with antibodies against H4Ac. ChIP-qPCR for H4Ac in EOL-1 cells carrying *DNMT3A*-R882H mutation and *DNMT3A*-WT revealed a reduction of H4Ac enrichment at the *HOXB* promoter regions with R882H mutation compared to *DNMT3A*-WT (Fig. 4d). Similarly, qRT-PCR analyses showed that primary BM cells harboring both *KMT2A*-PTD and *DNMT3A*-MT upregulated *HOXB* (B2, B3, B4, and B5) expression compared to cells with *KMT2A*-PTD/*DNMT3A*-WT (Fig. 4e). Immunoblot data showed that DNMT3A expression was downregulated in *KMT2A*-PTD/*DNMT3A*-MT AML cells; conversely, the expression of BCL2 and CDK1 was upregulated in similar cells compared to *KMT2A*-PTD/*DNMT3A*-WT AML cells (Supplemental Fig. S4). However, the expressions of H3K4me2, H3K4me3, H3, H3Ac, and H4Ac was comparable between the two groups (Supplemental Fig. S4).

### DNMT3A-MT induces modifications of genomic methylation patterns in KMT2A-PTD-positive EOL-1 cells

**Fig. 5:**
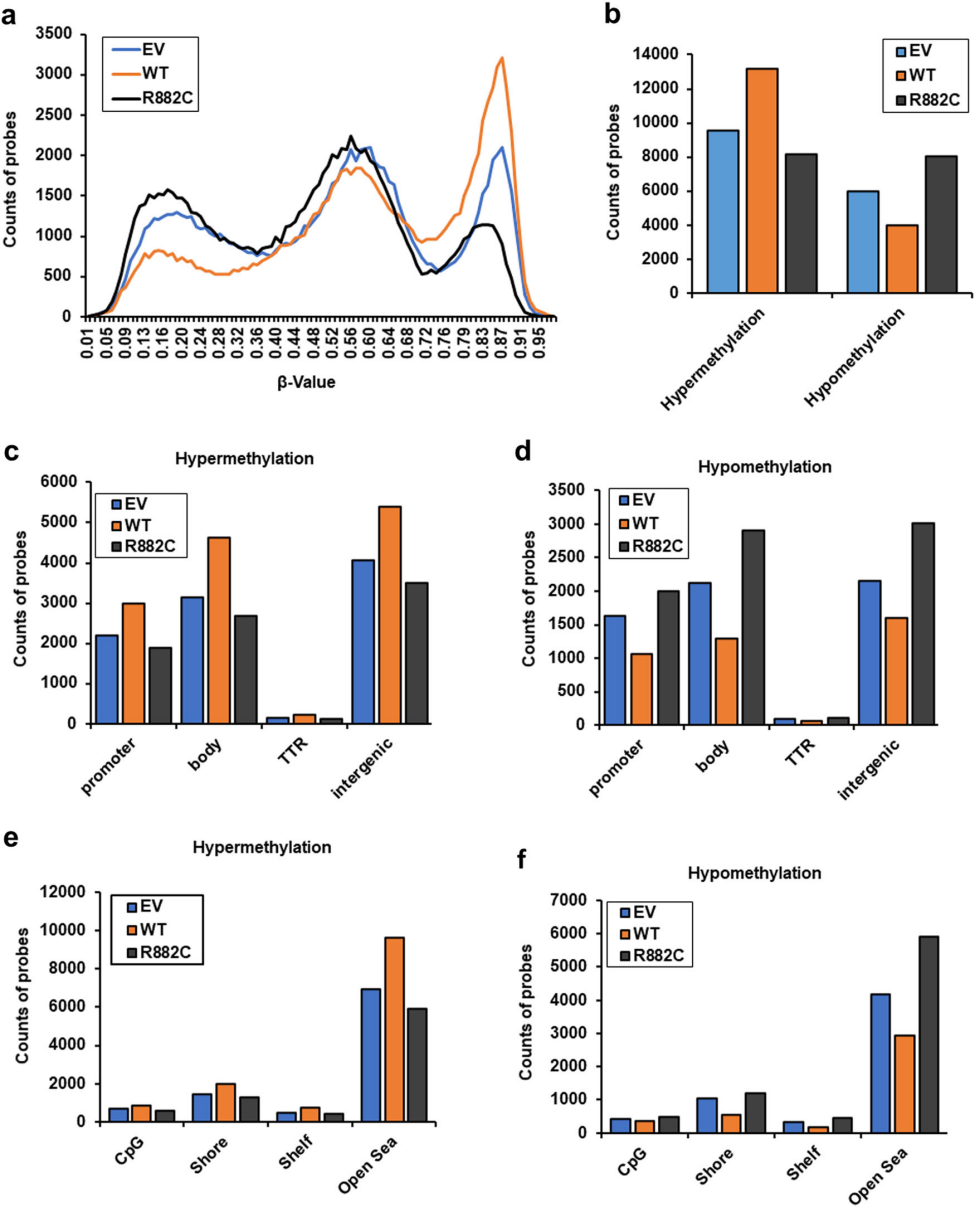
*DNMT3A*-R882 mutant induces modifications of genomic methylation patterns in transduced EOL-1 cells. **a** Distribution of genomic methylation pattern (*β* value) in the whole genome of EOL-1 cells transduced with EV control, *DNMT3A*-WT, and *DNMT3A*-R882C. **b** Hypo- and hypermethylation probes count obtained from transduced EOL-1 cells shown as bar-diagram. *β* value < 0.25 and >0.75 considered as hypomethylation and hypermethylation peaks, respectively. **c**, **d** The total hypermethylation and hypomethylation probes counted in each region defined by genomic structure shown in bar graph. **e**, **f** Methylation patterns in four regions defined by the distance from CpG islands, such as CpG islands, Shore, Shelf, and Open Sea region were shown.

Next, we analyzed the DNA methylation status of EOL-1 cells transduced with WT-, mutant *DNMT3A*, and control to assess whether *DNMT3A* mutant altered gene expression profiles were due to their changes of methyltransferase activity. Indeed, both DNA-hypomethylation and hypermethylation features were observed in the specific region throughout the whole genome (Fig. 5a). Overall, R882C mutation was more hypomethylated and less hypermethylated compared to EV or WT-expressing EOL-1 cells (Fig. 5b). Also, the changes in hypo- and hypermethylation patterns were seen in the context of gene structure, namely promoter, gene body, the transcriptional termination region (TTR), and the intergenic region. We found that R882C mutation was more hypomethylated in the intergenic and gene body regions, whereas WT- and control cells were more hypermethylated in those regions (Fig. 5c, d). We then examined the methylation patterns in four regions defined by the distance from the CpG islands^18^, such as CpG islands, Shore, Shelf, and Open Sea regions. Most of the hypo- and hypermethylation patterns were identified in the Open Sea region (Fig. 5e, f). In the context of gene methylation patterns, we found that the *CCL5* gene was differentially methylated in promoter regions and *AREG* mainly in gene body region (Supplemental Fig. S5a, b) of *DNMT3A*-R882C EOL-1 cells. To understand the relationship between global DNA methylation and gene expression changes, we examined upregulated genes in *KMT2A*-PTD AML samples with *DNMT3A*-MT compared to *KMT2A*-PTD with *DNMT3A*-WT (Supplemental Dataset S1). We analyzed the DNA methylation status of those genes in EOL-1 cells expressing DNMT3A-WT and R882C mutation. We observed that 399 genes out of 762 upregulated genes were less methylated (differential *β* value < −0.3) in EOL-1 cells expressing R882C compared to DNMT3A-WT (Supplemental Dataset S3), indicating the reduction of methyltransferase activity due to *DNMT3A* mutation. In contrast, 49 genes were more methylated (differential *β* value > 0.3) in EOL-1 cells expressing R882C compared to *DNMT3A*-WT (Supplemental Dataset S4a). Whereas genomic features of 29 genes showed both decreased (differential *β* value < −0.3) and increased (differential *β* value > 0.3) methylation at different genomic regions in EOL-1 cells expressing R882C compared to *DNMT3A*-WT (Supplemental Dataset S4b). Moreover, 276 genes of 762 upregulated genes were hypomethylated (*β* value < 0.25) in EOL-1 cells expressing R882C compared to *DNMT3A*-WT (Supplemental Dataset S5).

### Expression of DNMT3A-MT in Kmt2a-PTD-expressing mouse BMC enhanced clonogenic and self-renewal activity

**Fig. 6:**
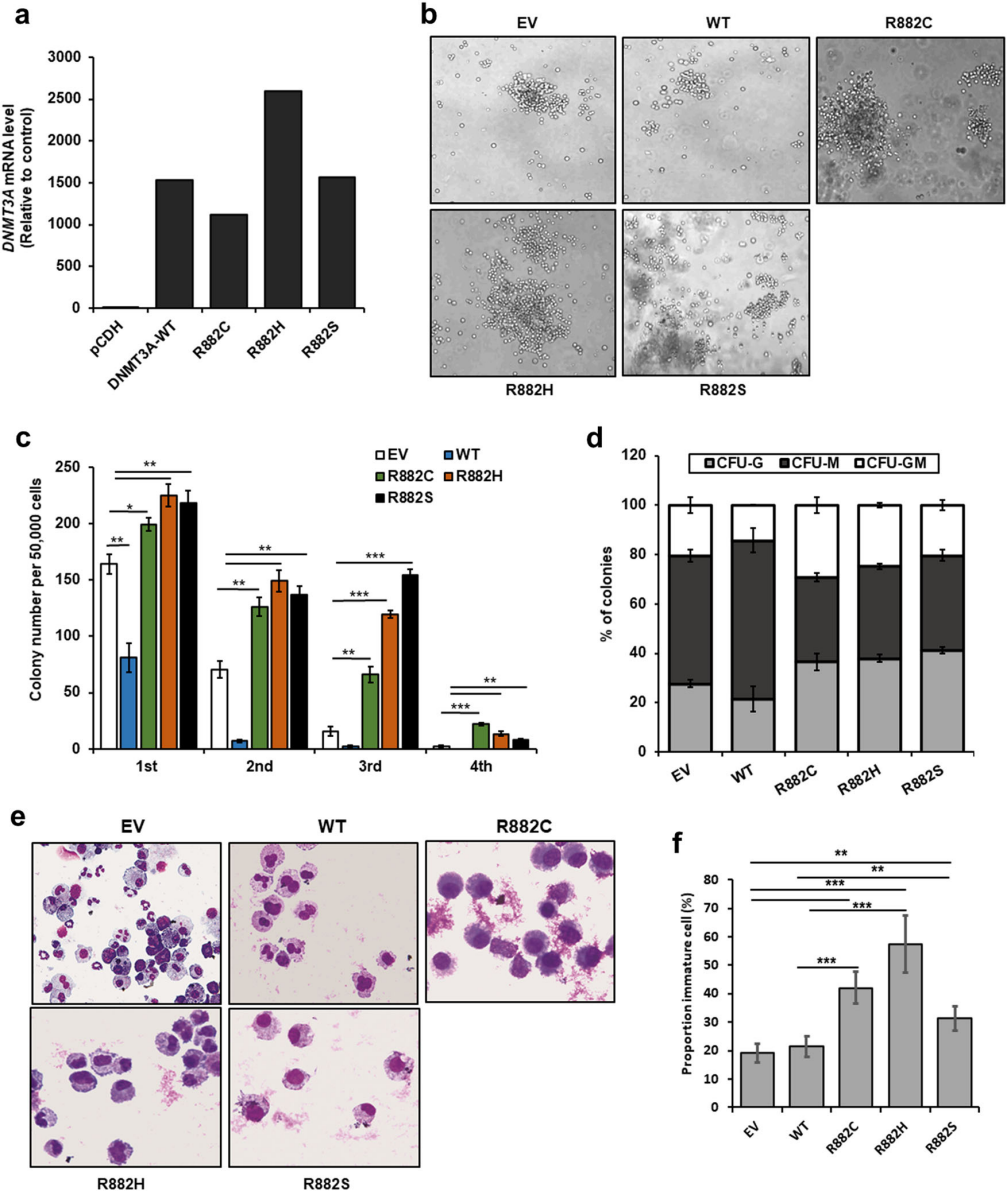
Addition of *DNMT3A*-MT in *Kmt2a*-PTD mouse BM cells increased colony-forming and self-renewal activity. **a** Lentivirus-mediated transduction of *DNMT3A*-WT/mutants in *Kmt2a*-PTD-positive mouse BM cells and *DNMT3A* mRNA level was detected by quantitative RT-PCR using first colony-forming cells. **b**, **c** Colony-forming potentials of *Kmt2a*-PTD^+^ mouse BM cells transduced with indicated plasmids in methylcellulose colony-forming media, representative images of colonies in the second-round plating were shown. Colonies at least 50 cells were scored on day 8 of cultures. Colonies were photographed and counted manually, original magnification: ×100. Columns represent the number of serially replated colonies. Error bars represent the mean ± s.d. from duplicated cultures. *P < 0.05, **P < 0.03, ***P < 0.01, compared with the control. **d** Proportion of CFU-colonies of second-round serial replating were shown. **e** Cytospin smeared preparations of cultured cells in the first plating colonies assays by Liu’s reagents staining were shown. **f** Statistical analysis of the numbers of immature cells in the first plating colonies cells. Error bars represent the mean ± s.d. of 4–5 different microscopic fields. **P < 0.01, ***P < 0.001, compared with the control. Two-sided Student’s *t* test was used to calculate the *P* value.

To determine the effect of *DNMT3A-*MT on clonogenicity and self-renewal activity in mouse BMC harboring *Kmt2a*-PTD, we transduced *Kmt2a*-PTD-positive mouse BMC with *DNMT3A*-WT/MT and EV, and transgene expressions were checked by qRT-PCR (Fig. 6a). We examined their colony-forming ability (Fig. 6b) and self-renewal activity (Fig. 6c) in M3434 medium and analyses type of colonies, granulocyte, macrophage, and granulocyte with macrophage (Fig. 6d). However, we could not observe erythroid (CFU-E) and burst-forming unit erythroid (BFU-E) colonies. We found that *DNMT3A*-MT-transduced *Kmt2a*-PTD-positive BM cells had significantly greater colony-formation ability with relatively immature morphology (Fig. 6c, e, f) compared with either *DNMT3A*-WT or empty vector control BM cells. Moreover, *DNMT3A*-MT-transduced cells produced significantly more colonies in the second- and third-round plating compared with *DNMT3A*-WT-expressing cells (Fig. 6c). Similarly, more dense colonies were seen in second-round plating of the *DNMT3A*-MT/*Kmt2a*-PTD BM cells indicating that *DNMT3A*-MT cells may maintain an immature phenotype longer than *DNMT3A*-WT cells.

### The collaboration of DNMT3A-MT with Kmt2a-PTD in the myeloid transformation

**Fig. 7:**
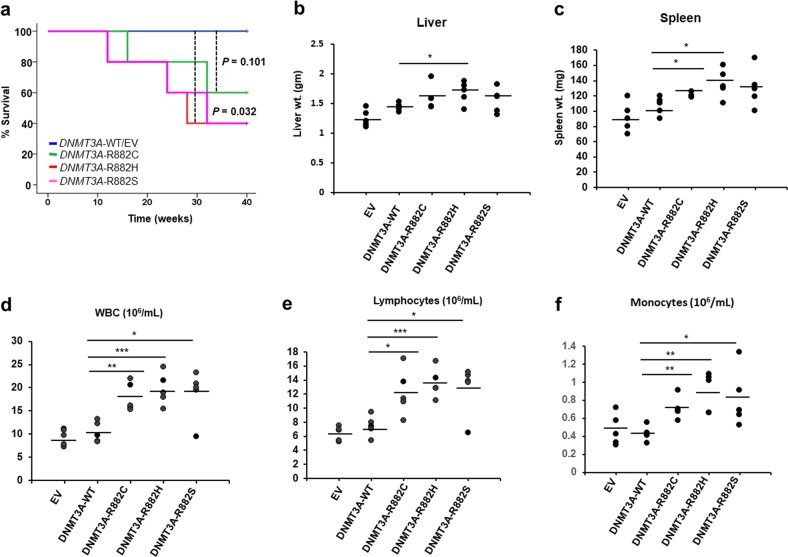
The collaboration of *DNMT3A-MT* with *Kmt2a*-PTD shows shorter latency in vivo. **a** Kaplan−Meier curve shows the survival of mice transplanted with BM cells harboring *Kmt2a* and transduced with empty vector (EV, *n* = 5) control, *DNMT3A*-WT, *DNMT3A*-R882C/H/S (*n* = 5). *P* values were calculated using a log-rank test. **b**, **c** Quantification of hepatomegaly (**b**) and splenomegaly (**c)** in mice transduced with EV, *DNMT3A*-WT, *DNMT3A*-R882C/H/S were shown (*n* = 5). **d**−**f** Peripheral blood counts of transduced mice were shown (*n* = 5 for each group). *P < 0.05, **P < 0.01, ***P < 0.005, compared with the *DNMT3A*-WT group.

To explore the in vivo transformation effect of *DNMT3A-*WT*/*MT with *KMT2A-*PTD, we carried out bone marrow transplantation (BMT) experiments using murine BMC harboring *Kmt2a*-PTD, lentiviral transduced with *DNMT3A*-WT/MT, and EV constructs. To determine whether susceptibility to hematologic malignancy was affected by the cooperation of Kmt2a-PTD with DNMT3A-WT/MT, we monitored mice up to 10 months. Mice carrying either empty vector or DNMT3A-WT displayed no hematologic abnormalities. At 10 months post transplantation, 2 and 3 of 5 mice transplanted with *Kmt2a*-PTD-BM cells expressing *DNMT3A*-R882C and *DNMT3A*-R882H/S mutants, respectively, died with marked splenomegaly and hepatomegaly compared with *Kmt2a*-PTD/EV-transplanted mice (Fig. 7a−c). The survival of DNMT3A-WT/MT and EV control mice are shown in Fig. 7a. In contrast, all of the five mice transplanted with *Kmt2a*-PTD BM cells expressing *DNMT3A*-WT constructs were healthy and alive at the same time. Peripheral blood counts showed leukocytosis (Fig. 7d−f and Supplemental Fig. S6a) but there was no anemia or thrombocytopenia in the *Kmt2a*-PTD/*DNMT3A*-MT-transplanted mice (Supplemental Fig. S6b, c). Flow cytometric analyses of lineage markers from the bone marrow cells of *Kmt2a*-PTD/*DNMT3A*-MT-transplanted mice revealed more Sca-1-, cKit/Sca-1- and CD34-positive cells and lesser number of Mac-1 (CD11b) and CD14-positive cells (Supplemental Fig. S7).

## Discussion

It is unclear how *KMT2A*-PTD contributes to AML. Potential mechanisms of how *KMT2A*-PTD regulates normal hematopoiesis have been reported^19,20^. Repetitive DNA-binding domain in *KMT2A*-PTD exhibits transcriptional potential in vitro^19^ and KMT2A-PTD contributes to the leukemic phenotype in *KMT2A*-WT AML cells via a recessive gain-of-function mechanism^20^. Little is known about the mechanism of crosstalk and cooperation between *KMT2A-*PTD and other gene mutations in the leukemogenesis. We have observed a high frequency of coexistence of *DNMT3A* mutations with *KMT2A*-PTD; more importantly, it conferred a very poor outcome in our AML patients^12^. Moreover, most *DNMT3A* mutations (67.7%) were in the methyltransferase domain at amino acid R882^12^. The present results suggested that *DNMT3A*-R882 mutations with *KMT2A*-PTD promoted myeloid malignancy transformation in vitro and in vivo.

In accordance with the in vitro cooperation of *DNMT3A*-MT with *KMT2A*-PTD, we observed the transduction of *DNMT3A*-MT in EOL-1 cells, a *KMT2A*-PTD-positive leukemias cell line, augmented cell growth and clonogenicity, as well as increased self-renewal activity of the transduced-primary murine BMC-expressing *Kmt2a*-PTD. The EOL-1 cell line endured eosinophilic differentiation upon Na-butyrate treatment and ATRA regulated the expression of CD11b but did not induce mature eosinophil morphology^21,22^. During the eosinophilic differentiation of EOL-1 cells, the expression of CD11b was increased under differentiating stimuli^23,24^. In primary human BM cells expressing *KMT2A*-PTD and *DNMT3A*-MT showed hyperproliferation and self-renewal clonogenic potential compared to *DNMT3A*-WT with *KMT2A*-PTD, suggesting a critical role of mutant *DNMT3A* in AML patients with *KMT2A*-PTD. It was reported that ATRA reduced cell growth with the induction of apoptosis of EOL-1 cells^22^. We found that on the treatment of either ATRA or SAHA, *DNMT3A*-WT/MT-transduced EOL-1 cells showed inhibition of cell growth, and the combination of both agents was more effective than a single agent. However, ATRA was more potent than SAHA in transduced EOL-1 cells. The growth-inhibitory potency of ATRA or SAHA on EOL-1 cells was higher compared to U937 cells indicating that the mechanism of growth inhibition of these two drugs was different in different cell lines harboring endogenous *KMT2A* or *KMT2A*-PTD. Moreover, ATRA and SAHA significantly reduced the proliferation of primary *KMT2A*-PTD AML cells with *DNMT3A*-WT/MT. However, these agents were more active in the reduction of cell growth in *KMT2A*-PTD/*DNMT3A*-WT AML cells compared to *KMT2A*-PTD/*DNMT3A*-MT cells. One possible explanation of this finding was that *DNMT3A* mutations induced HSC expansion, cooperated with *KMT2A*-PTD to induce AML and contribute to the resistance to these drugs. Though it might be possible that cooperation of other oncogenic abnormalities with *DNMT3A* mutation in EOL-1 cells, the co-occurrence of *KMT2D*-PTD with *DNMT3A* mutation should play a role at least partly in the leukemogenesis in the current situation as *DNMT3A* mutations frequently coexist with *KMT2A*-PTD. Previously, it was demonstrated that the combination of decitabine, a hypomethylating agent and HDAC (histone deacetylase) inhibitor, vorinostat (also known as SAHA) decreased the transcription ratio of the *KMT2A*-PTD-to-*KMT2A*-WT alleles and simultaneously induced apoptosis in *KMT2A*-PTD-positive blasts^25^. In our animal model, we could not find an acute leukemia transformation in *Kmt2a*-PTD mice with *DNMT3A*-MT, but we observed hematologic abnormalities with shorter latency and hepatosplenomegaly compared to control or *DNMT3A*-WT mice. It has been reported that either *KMT2A*-PTD or *Flt3*-ITD do not develop AML; however, the cooperation of *Flt3*-ITD with *Kmt2a*-PTD developed acute leukemia in a mouse model^26^. It was also reported that the *Dnmt3a* mRNA level was increased in BM from double knock-in (*Kmt2a*-PTD and *Flt3*-ITD) mice compared to single knock-in or WT control^26^. However, we observed that downregulation of the DNMT3A level in *KMT2A*-PTD/*DNMT3A*-MT AML primary cells with *FLT3*-ITD or without *FLT3*-ITD relative to *KMT2A*-PTD/*DNMT3A*-WT, suggesting that *DNMT3A*-WT/MT status had maintained DNMT3A expression rather than *FLT3-*ITD status. Previously, Huang et al.^27^ reported that *DNMT3A* expression was significantly decreased with *DNMT3A*-R882 mutations and the reduced expression levels were associated with a lower complete remission rate in the AML patients. It was also reported that Arg882 mutants reduced their DNA methylation activities compared to DNMT3A-WT^28^. Therefore, lower amounts of DNMT3A protein might reduce enzymatic activities in AML patients with DNMT3A mutations, which regulate crucial gene expression in the pathogenesis of AML in *KMT2A-*PTD-positive leukemic cells. Recently, we found that overexpression of *DNMT3A*-R882C/H mutations enhanced clonogenicity, cell proliferation, and growth with impairment of apoptosis in the endogenously *KMT2A*-WT-expressing U937 and HL-60 cells with different mechanisms^29^.

To test the transformational ability of *DNMT3A* mutation in *KMT2A*-PTD AML, we investigated gene expression profiles of BM cells of *KMT2A*-PTD with *DNMT3A*-WT/MTs. Previously, we found that AML with *KMT2A*-PTD can be distinguished from AML with *KMT2A-*translocation on the basis of *HOXB* gene clusters^14^. Analyses of a subset of the same microarray dataset, we found that AML with *KMT2A*-PTD/*DNMT3A* mutations enhanced the *HOXB* gene expression compared with *KMT2A*-PTD/*DNMT3A*-WT which was consistent with our in vitro data in EOL-1 cells expressing *DNMT3A*-WT/MT. These results demonstrated that the DNMT3A function was important for *HOXB* gene regulation in human leukemia cells harboring *KMT2A*-PTD. Similarly, primary BM cells from AML patients with *KMT2A*-PTD/*DNMT3A* mutations showed higher *HOXB* expression compared to *KMT2A*-PTD/*DNMT3A*-WT, which indicate a critical role of *HOXB* in AML with *KMT2A*-PTD/*DNMT3A*-MT. We observed that *KMT2A*-PTD was frequently associated with *FLT3*-ITD in AML^12^; however, previously using three large-scale datasets analyses, we reported that upregulation of *HOXB* in *KMT2A*-PTD might not be related to *FLT3*-ITD status^14^. Gene expression profiling analyses of transduced U937 cells with *DNMT3A*-MT revealed aberrant expression of several cell-cycle and apoptosis-related genes; however, no differentially expressed *HOXB* compared to *DNMT3*-WT-transduced U937 cells have been reported recently (accession number GSE90934)^29^. It was demonstrated that *Kmt2a*-PTD induced aberrant *Hox* expression including *Hoxa7*, *Hoxa9*, and *Hoxa10* in a mouse model^30^. In contrast, we could not find the upregulation of *HOXA* including *HOXA9* in our study. We also found that the expression of series of genes associated with HSC activation, myeloid differentiation and cell survival, such as *MEIS1*, *HBEGF*, *BCL6*, and *MCL1*, was significantly upregulated in patients with *KMT2A*-PTD/*DNMT3A*-MT. The transcription factor *Meis1* was associated with long-term hematopoiesis and always coactivated with *Hoxa7* or *Hoxa9* in leukemic mice^31,32^. In addition to the upregulation of *MCL-1*, pathways involved in granulocytic/monocytic lineage proliferation, including *HOXB2*, *RAB20*, *SOCS3*, and *MEIS1*, were activated, and those involved in platelet activation, including *PRKCA*, *PF4*, and *ITGA2B*, were upregulated in *KMT2A*-PTD AML with *DNMT3A* mutations. RNA modifications, including RNA methylation, mRNA processing, and RNA splicing, play a critical role in important biological processes^33,34^. RNA modifications have been associated with embryonic stem cell maintenance and differentiation^35–37^. By using GSEA analyses of primary *KMT2A*-PTD AML with *DNMT3A*-WT/MT, we identified several deregulated pathways. Among them, the RNA methylation pathway is critical in AML, as the previous study of other investigators showed that genetic alterations of m6A (N6-methyladenosine) regulatory genes are associated with worse survival and inferior outcome in AML patients^38^. N6-methyladenosine was the most established and internal modification that occurred in the mRNA which was catalyzed by a methyltransferase complex containing methyltransferase-like 3 (METTL3), RNA methyltransferase-like 1 (RNMTL1) and methyltransferase-like 1 (METTL1), were downregulated, and Wilm’s-tumor-1-associated protein (WTAP)^33,39^ was upregulated in *KMT2A*-PTD AML with *DNMT3A* mutations.

DNMT3A is a DNA methyltransferase that catalyzes DNA methylation. Both KMT2A and DNMT3A regulate normal hematopoiesis. Cancer progression involves a diversity of hypo- and hypermethylated genomic regions, resulting in dramatic alterations in gene expression patterns^40^. From DNA methylation microarray analyses using transformed EOL-1 cells, we observed that *DNMT3A*-R882C mutation had both DNA methylation-dependent and -independent effects and exhibited distinct mechanism for leukemogenesis. *DNMT3A*-R882C-expressing EOL-1 cells were more hypomethylated in the gene body and intergenic regions rather than promoter region compared to *DNMT3A*-WT-expressing cells. However, there was no much difference in the CpG region. Similar results were observed in U937 cells transduced with *DNMT3A*-WT/MT (GSE90934)^29^. The results indicated that *DNMT3A*-WT/MT but not *KMT2A*-WT or *KMT2A*-PTD were responsible for the hypo- or hypermethylation features in cells. We next compared the DNA methylation status and gene expression changes in EOL-1 and primary AML cells, respectively using two different datasets. It was observed that many upregulated genes in *KMT2A*-PTD AML with *DNMT3A*-MT were less methylated and some genes were more methylated in EOL-1 cells expressing *DNMT3A*-R882C mutation compared to *DNMT3A*-WT, indicating that *DNMT3A* mutations controlled the gene expression changes by both DNA methylation-dependent and -independent manner in *KMT2*A-PTD AML. It was observed that a group of upregulated genes associated with HSC activation and positive regulation of cell proliferation, including *CD34*, *HBEGF*, *STOX2*, *MEIS1*, *MCL1*, *PCNX*, *AREG*, and *HLX*, less methylated in EOL-1 cells expressing *DNMT3A*-R882C mutation compared to *DNMT3A*-WT. Loss of *Dnmt3a* was shown to upregulate multipotency genes, downregulate differentiation factors, and progressively impair differentiation in HSCs, attributable to both increased and decreased methylation at distinct loci^41^. The previous study illustrated that *DNMT3A* mutations with reduced enzymatic activity or aberrant affinity to histone H3 would alter DNA methylation patterns and/or gene expression profiles such as *HOXB* upregulation due to hypomethylation in AML^28^.

Up to date, to the best of our knowledge, the present study is the first to determine the biological role of *DNMT3A* mutation in *KMT2A*-PTD-positive cells, which might contribute to leukemogenesis in vitro and in vivo. The present study provides additional information underlying the pathogenic role of DNMT3A-MT with KMT2A-PTD in proliferating advantage with stem cell signature, enhanced self-renewal activity and aberrant *HOXB* expression in human leukemia, which may help to better understand the disease and to design better therapy for AML patients with these mutations.

## Materials and methods

### Reagents and antibodies

RPMI 1640, FBS and antibiotic-antimitotic were purchased from Thermo Fisher Scientific (Waltham, MA, USA); puromycin (101-58-58-2) from MD Bioscience; human and mouse methocult medium, MethoCult H4535 and MethoCult M3434 respectively from STEMCELL Technologies (Vancouver, BC, Canada); mouse stem cell factor and recombinant mouse IL-3 from R&D (Minneapolis, MN, USA); polybrene (H9268), ATRA (R2625), SAHA (SML0061) and β-Actin (A5441) from Sigma-Aldrich (St. Louis, MO, USA); anti-DNMT3A (#3598) from Cell Signaling Technology (Danvers, MA, USA); ChIP Assay Kit (#17–295), anti-H3K4me2 (#07–030), anti-H3Ac (#17–615), anti-H4Ac (#06866) from Millipore (Temecula, CA, USA); anti-H3K4me3 (ab8580), anti-BCL2 (ab692) and anti-H3 (ab70550) from Abcam (Cambridge, UK); Liu’s reagents A and B (03R011/03R021) from ASK Biotech (Taiwan); trypan blue from Gibco-Life Technologies Corporation (Grand Island, NY, USA) and anti-CDK1 (# GTX20018) from GeneTex (Irvine, CA).

### Cell lines, mouse and human primary BM cells

EOL-1 and U937 cells were cultured in RPMI-1640 medium supplemented with 10% fetal bovine serum (FBS) and 1× antibiotic-antimycotic in a humidified chamber with 5% CO_2_ atmosphere at 37 °C. EOL-1 cell line was obtained from the laboratory of Dr. Gang Huang, Cincinnati Children’s Hospital Medical Center (Cincinnati, USA) and U937 from our own stocks. Both cell lines were authenticated by cellular morphology and STR analysis at Chang Gung Memorial Hospital (CGMH) (January−February 2017). Primary human BM cells were collected at initial diagnosis according to the CGMH guidelines after getting written consent from patients. The study was approved by the Institutional Board of CGMH. Liquid nitrogen storing (<1 year) cells were cultured in MethoCult H4535 medium for 5 days, then washed with complete RPMI medium and cultured in RPMI medium containing 20% FBS, 20% conditional medium (5637 cells cultured soup) and 1× antibiotic; medium changed every 2 days interval. BM samples having more than 90% of viable cells were selected for biological analyses. *Kmt2a*-PTD^+^ mouse BM cells were generated and maintained in the laboratory of Dr. Gang Huang. The development of the *Kmt2a*^PTD/WT^ mouse has been described previously^10,11^. Mouse BM cells were maintained by the M3434 MethoCult medium. In each experiment, cells were washed by phosphate-buffered saline (PBS) and subsequently cultured in RPMI 1640 medium supplemented with 20% FBS; 1× antibiotic-antimitotic, 100 ng/ml mouse stem cell factor, and 10 ng/ml recombinant mouse IL-3. For morphological studies, cytospin smears were stained with Liu’s reagents. Digital images were acquired using Olympus (model no. U-TV0.5XC-3) microscope equipped with a digital camera.

### Plasmid construction, lentiviral preparation, and infection

The full-length cDNA of human *DNMT3A-*WT and point mutants, R882C, R882H, and R882S, were constructed as described previously^29^. Lentiviruses production and infection were performed as described previously^42^. The infected cells were selected with 2 µg/ml puromycin for 2 weeks to obtain stable clones.

### Mice and bone marrow transplantation (BMT) experiment

All animal care and experiments were approved by the Department of Animal Experimentation at CGMH. Female C57BL/6 mice (NARLabs, Taiwan) aged 6−8 weeks were used for the BMT experiments. Mouse BMT was performed as described previously^43^. Briefly, the murine BM cells harboring *Kmt2a*-PTD were used for transduction of *DMMT3A*-WT/MT with lentiviruses by spun inoculation in the presence of 8 μg/ml polybrene. The infection was repeated after 48 h of the first infection and transduced BM cells (1 × 10^6^ cells/mouse) were transplanted into intraperitoneally injected busulfan (a single dose of 30 mg/kg prior to 3 days) mice via tail vein^44,45^. Mice were monitored every day up to 10 months and moribund mice were euthanized according to the animal house guidelines.

### Cell proliferation assay

Viable cells were assessed with either the trypan blue exclusion method or using flow cytometry analyses staining with Annexin V and propidium iodide at different time points. To check the sensitivity of different drugs, 1 × 10^5^/ml EOL-1 and U937 cells were cultured in the presence of ATRA and SAHA at different concentrations for 72 h and single dose of each drug was selected to test the activity in transformed EOL-1 cells. To check cell proliferation assays, 2 × 10^5^/ml human primary cells were cultured in liquid medium for 3 and 6 days. To check the sensitivity of ATRA and SAHA, human primary cells were cultured in the presence of 200 nM ATRA and 600 nM SAHA for 3 days, respectively. The percentage of viable cells was calculated by comparing the number of drug-treated cells to that in the untreated control cells.

### Colony formation and self-renewal activity assays

For clonogenic growth assays, transformed stable EOL-1 cells were cultured in 12-well plate at 2 × 10^3^ cells/well in 1% methylcellulose containing RPMI medium supplemented with 10% FBS for 10 days. 5 × 10^4^ transduced mouse BM cells were mixed with 2 ml MethoCult M3434 medium in six-well plate and cultured for 8 days. For human primary BM colony-formation assays, cells were seeded in six-well plates with 2 ml H4535 medium at a density of 5 × 10^4^ cells/well. The photograph was taken by a phase-contrast microscope (Nikon Eclipse TS100, Japan). For serial replating assays, all cells were harvested, washed twice with RPMI medium and counted. A similar number of cells were then replated and the process was repeated four times to check colony formation and self-renewal activity. The results from colony-forming unit (CFU) assays assessed granulocyte (CFU-G; colorless, more dense smaller and round cells), macrophage (CFU-M; colorless, less dense, large and elongated cells), granulocyte with macrophage (CFU-GM; colorless, heterogeneous population of small, round cells and large, elongated cells) and erythroid (CFU-E; red color either very small colonies or BFU-E; clusters containing group of tiny cells in irregular shape).

### Flow cytometry analysis

Flow cytometry experimentation was described in a previous publication^29^. Anti-CD11b PE (#12–0112) was purchased from eBioscience (San Diego, CA) and the apoptosis was detected by Annexin V antibody and propidium iodide staining according to the manufacturer’s instructions (Annexin V-FITC kit, #556547, BD Bioscience, CA).

### Isolation of RNA, reverse transcription and quantitative real-time polymerase chain reaction (qRT-PCR)

Methods were described previously in detail^29^. Briefly, after RNA extraction, samples were reverse-transcribed to cDNA and used for quantitative PCR with iQTM SYBR® Green Supermix (ABI, Thermo Fischer Scientific) according to the manufacturer’s protocol. The sequences of oligonucleotides used for qRT-PCR are listed in Supplementary Table S4.

### Immunoblotting analysis

Immunoblot was performed with the following antibodies: anti-DNMT3A, anti-H3K4me2, anti-H3Ac, anti-H4Ac, anti-H3K4me3, anti-BCL2, anti-H3, anti-CDK1, and anti-β-actin. Cell lysates were extracted with RIPA buffer and subjected to SDS-PAGE and Western blot analysis as described previously^29^. A loading control of the detection of β-actin was included for all immunoblots.

### Chromatin immunoprecipitation (ChIP) analysis

The ChIP assays were carried out according to the manufacturer’s (Millipore) protocol. Briefly, 1 × 10^6^ transduced EOL-1 cells were used for each immunoprecipitation. Cells were cross-linked by adding 1% formaldehyde, collected, resuspended in ChIP lysis buffer and sonicated to obtain chromatin fragments in a size between 200 and 1000 base pairs. DNA−protein complexes were then immunoprecipitated with a 5 µl anti-H4Ac antibody. Protein A Agarose/Salmon Sperm DNA beads were used to capture the antibody−chromatin complex. The immunoprecipitated samples and an aliquot of the input were subjected to reverse cross-link. Purified CHIP product was quantified by RT-PCR using SYBR Green on an ABI Prism 7900HT Fast Real-Time PCR system (Thermo Fisher Scientific). Quantitative real-time PCR quantification of ChIP was performed in duplicate using primers (Supplementary Table S5) specific for promoter regions.

### DNA methylation microarray analysis

Genome-wide DNA methylation was assessed using the Illumina Infinium Human MethylationEPIC Beadchip (Illumina Inc, CA, USA) according to the manufacturer’s instructions. Briefly, genomic DNA was extracted and purified from all samples using a column-based DNA extraction kit (Qiagen DNeasy Kit) according to the standard method. Five hundred nanograms of DNA of each sample was subjected to bisulfite conversion with the EZ DNA methylation kit (Zymo Research) according to the manufacturer’s instructions. Bisulfite converted DNA was amplified, fragmented and hybridized to Illumina Infinium Human MethylationEPIC Beadchip using standard Illumina protocol. The sample was labeled with Cy5 and Cy3 using Illumina standard protocol. Arrays were imaged using iSCAN using standard recommended Illumina scanner setting. Illumina GenomeStudio software v2010.3 was used to extract the raw signal intensities of each probe (without background correction or normalization). The resulting raw data were normalized (control normalization) and background corrected by the manufacturer to generate methylation *β* values. Genomic methylation patterns (hypo- or hypermethylation) of different samples were analyzed after the variance filter (STD > 0.2) and raw data (without filter) used for the analyses of a specific gene methylation pattern. *β* values <0.25 and >0.75 were considered as hypomethylation and hypermethylation peaks, respectively. The data, including all raw genomic-methylation data, have been deposited in the Gene Expression Omnibus (GEO) database with accession number GSE109364.

### Statistical analysis

The Kaplan–Meier analysis was used to evaluate mouse survival. Differences in survival were assessed using the log-rank test. All in vitro data represented here are mean ± s.d. as indicated. The significance of the differences between groups was determined using the two-sided Student’s *t* test. A *P* value of <0.05 was considered significant for all analyses.

## Supplementary information

Supplementary

Figure S1

Figure S2

Figure S3

Figure S4

Figure S5

Figure S6

Figure S7

Table S1

Table S2

Table S3

Table S4

Table S5

Dataset S1

Dataset S2

Dataset S3

Dataset S4

Dataset S5
