# Supplementary material for: DNMT3A mutants provide proliferating advantage with augmentation of self-renewal activity in the pathogenesis of AML in KMT2A-PTD-positive leukemic cells
Source: Oncogenesis. 2020 Feb 3;9(2):7. doi: 10.1038/s41389-020-0191-6 (PMC6997180; doi:10.1038/s41389-020-0191-6)
Supplement: Supplementary file 1 — Supplementary [file 41389_2020_191_MOESM1_ESM.docx]

***DNMT3A* mutants provide proliferating advantage with augmentation of self-renewal activity in the pathogenesis of AML in *KMT2A-*PTD-positive leukemic cells**

**Rabindranath Bera,^1^ Ming-Chun Chiu,^1^ Ying-Jung Huang,^1^ Gang Huang, ^3^ Yun-Shien Lee^4^ and Lee-Yung Shih,^1,2*^**

^1^Division of Hematology-Oncology, Chang Gung Memorial Hospital at Linkou, Taiwan;

^2^Chang Gung University, Taoyuan, Taiwan;

^3^Divisions of Pathology and Experimental Hematology and Cancer Biology, Cincinnati Children’s Hospital Medical Center, 3333 Burnet Avenue, Cincinnati, Ohio 45229, USA.

^4^School of Health Technologies, Ming Chuan University, Taipei, Taiwan

**Supplementary Information**

**Supplementary Figure Legends S1-S7**

**Supplementary Table Legends S1-S5**

**Supplementary Material Legends**

**Supplementary Figure Legends S1-S7**

**Figure S1. KMT2A-PTD positive, EOL-1 and KMT2A-WT, U937 cells reduced proliferationa in the presence of different drugs as a dose dependent manner (related Fig. 1)**. **a** Viability of EOL-1 cells in the presence of different doses of ATRA (left panel) and SAHA (right panel) at 72 h incubation. **b** Viability of U937 cells in the presence of different doses of ATRA (left panel) and SAHA (right panel) at 72 h incubation. Cell proliferation was measured using trypan blue exclusion method. Error bars represent ± s.d. of the mean of duplicate cultures and each experiment repeated three times. ***P*<0.03, *P*-value compare to control cells was calculated; ns, not significant.

**Figure S2. Addition of DNMT3A-MT in KMT2A-PTD-positive EOL-1 cells modestly affected on differentiation. a, b** EOL-1 control cells without stimulant (**a**) and transformed EOL-1 cells were treated with 500 nM SAHA for 96 h; cell morphology with Liu’s reagents stained smears (original magnification: ×400) (**b**, left panel) and percentage of CD11b positive cells were shown (**b**, right panel). **c** Transformed EOL-1 cells were treated with 100 nM ATRA for 96 h; cell morphology with Liu’s reagents stained smears (original magnification: ×400). **d** Percentage of CD11b positive cells after treatment with 100 nM ATRA for 96 h. **e** EOL-1 cells were treated with 500 μM Na-butyrate for 96 h; cell morphology with Liu’s reagents stained smears (original magnification: ×400) (**e,** left, upper panel), representative flow cytometry (**e**, left, lower panel) and percentage of CD11b positive cells were shown (**e,** right panel). **f** Representative flow cytometry data to check CD11b expression of transformed EOL-1 cells treated with DMSO only for 96 h. Error bars (**b**) represent ± s.d. of the mean of 3 separate experiments and error bars (**d** and **e**) represent ±s.d. of the mean of 2 separate experiments. ***P* <.03, ****P* <.01, either compared with the control or as indicated in figures; n.s. not significant. Two-sided Student’s *t* test was used to calculate the *P* value.

**Figure S3. Validation of gene expression in EOL-1 cells (related Fig. 4).** **a** Quantitative RT-PCR of representative deregulated genes in transduced EOL-1 cells which were upregulated in *KMT2A*-PTD/*DNMT3A*-MT AML compared to *KMT2A*-PTD/*DNMT3A*-WT AML. Data are expressed as mean ± s.d. of three independent experiments. **P*<0.05, ****P*<0.005 compared to EV. Two-sided Student’s *t*-test was used to calculate the *P*-value. **b** Quantitative RT-PCR analyses of *HOXA5, HOXA7, HOXA9* and *HOXA10* expression in EOL-1 cells transduced with *DNMT3A*-WT/MT showing no change in different groups. Error bars represent ±s.d. of the mean of two separate experiments.

**Figure S4**. Representative immunoblot data of primary AML cells harboring *KMT2A*-PTD/*DNMT3A*-WT/MT. The values in immunoblot indicating normalized signal density corresponding to β-actin expression.

**Figure S5. Genomic methylation pattern of genes in transduced EOL-1 cells (related Fig. 5).** **a, b** Methylation pattern of *CCL5* and *AREG* genes showing the difference of methylation in different regions in EOL-1 cells transduced with EV, WT and R882C.

**Figure S6. Peripheral blood counts in BMT mice (related Fig. 7).** **a-c** Peripheral blood counts of transplanted mice with *Kmt2a*-PTD-BM cells expressing *DNMT3A*-WT, *DNMT3A*-R882C and *DNMT3A*-R882H/S mutants, and EV control were shown (n = 5 for each group). **P* <0.05, ***P* <0.01, compared with the *DNMT3A*-WT group.

**Figure S7. The immunophenotypic analysis of bone marrow cells in BMT mice (related Fig. 7).** Flow cytometric analyses of BM cells derived from mice transplanted with *Kmt2a*-PTD-BM cells expressing *DNMT3A*-WT, *DNMT3A*-R882C and *DNMT3A*-R882H/S mutants, and EV control at around 10 months post-BMT (n=3 of each group). *P*-value showing calculated as indicated in figures.

**Supplementary Table Legends S1-S5**

**Table S1.** Characteristic of *KMT2A*-PTD AML cells with *DNMT3A*-WT/MT patient samples used for primary culture

**Table S2.** Characteristic of *KMT2A*-PTD positive patient samples used for microarray analyses

**Table S3.** List of upregulated genes (>2 folds) in *KMT2A*-PTD/*DNMT3A*-MT vs *KMT2A*-PTD/*DNMT3A*-WT patient’s samples matched with fingerprint genes

**Table S4.** List of primer sets for quantitative RT-PCR to check mRNA expression of different genes

**Table S5.** List of primer pairs for Chromatin-immunoprecipitation (ChIP) quantitative RT-PCR to check the enrichment of H4Ac at different *HOXB* promoter regions

**Supplementary Material file Legends**

**Supplemental Dataset S1:** List of upregulated Genes (>2 folds) in *KMT2A*-PTD/*DNMT3A*-MT AML samples compared to *KMT2A*-PTD/*DNMT3A*-WT.

| \| **Supplemental Dataset S2:** List of downregulated Genes (<-2 folds) in *KMT2A*-PTD/*DNMT3A*-MT AML samples compared to *KMT2A*-PTD/*DNMT3A*-WT.  **Supplemental Dataset S3:** List of differentially methylated (differential β-value <-0.3) genes in *DNMT3A*-R882C-expressing EOL-1 cells compared to *DNMT3A*-WT-expressing EOL-1 cells, which were upregulated (>2 folds) in *KMT2A*-PTD/*DNMT3A*-MT AML cells compared to *KMT2A*-PTD/*DNMT3A*-WT cells \| \| \| --- \| --- \| \| **Supplemental Dataset S4:** **a**. List of differentially methylated (differential β-value >0.3) genes in DNMT3A-R882C-expressing EOL-1 cells compared to DNMT3A-WT-expressing EOL-1 cells, which were upregulated (>2 folds) in KMT2A-PTD/DNMT3A-MT AML cells compared to KMT2A-PTD/DNMT3A-WT cells.  **b**. List of differentially methylated genes (differential β-value both >0.3 and <-0.3) in the different genomic regions of DNMT3A-R882C-expressing EOL-1 cells compared to DNMT3A-WT-expressing EOL-1 cells, which were compared to KMT2A-PTD/DNMT3A-WT cells upregulated (>2 folds) in KMT2A-PTD/DNMT3A-MT AML cells.  **Supplemental Dataset S5:** List of hypomethylated (β-value <0.25) genes in DNMT3A-R882C-expressing EOL-1 cells compared to DNMT3A-WT-expressing EOL-1 cells, which were upregulated (>2 folds) in KMT2A-PTD/DNMT3A-MT AML cells compared to KMT2A-PTD/DNMT3A-WT cells. \| \|  \| \|  \| \|  \| |
| --- | --- | --- | --- | --- | --- | --- |
|  |
