# Supplementary figures and images for: DNMT3A mutants provide proliferating advantage with augmentation of self-renewal activity in the pathogenesis of AML in KMT2A-PTD-positive leukemic cells
Source: Oncogenesis. 2020 Feb 3;9(2):7. doi: 10.1038/s41389-020-0191-6 (PMC6997180; doi:10.1038/s41389-020-0191-6)

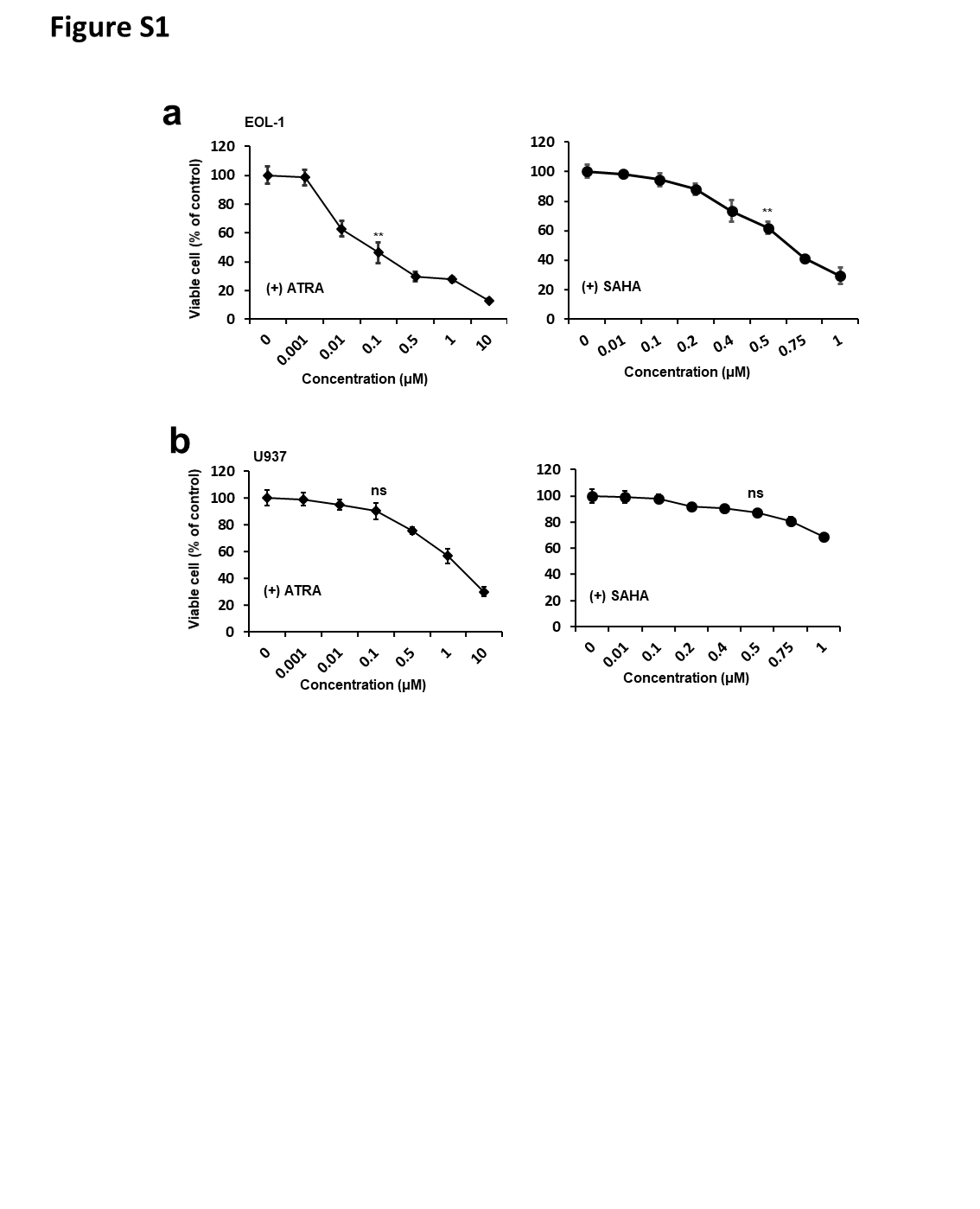

Supplement: Supplementary file 2 — Figure S1 [file 41389_2020_191_MOESM2_ESM.tif]

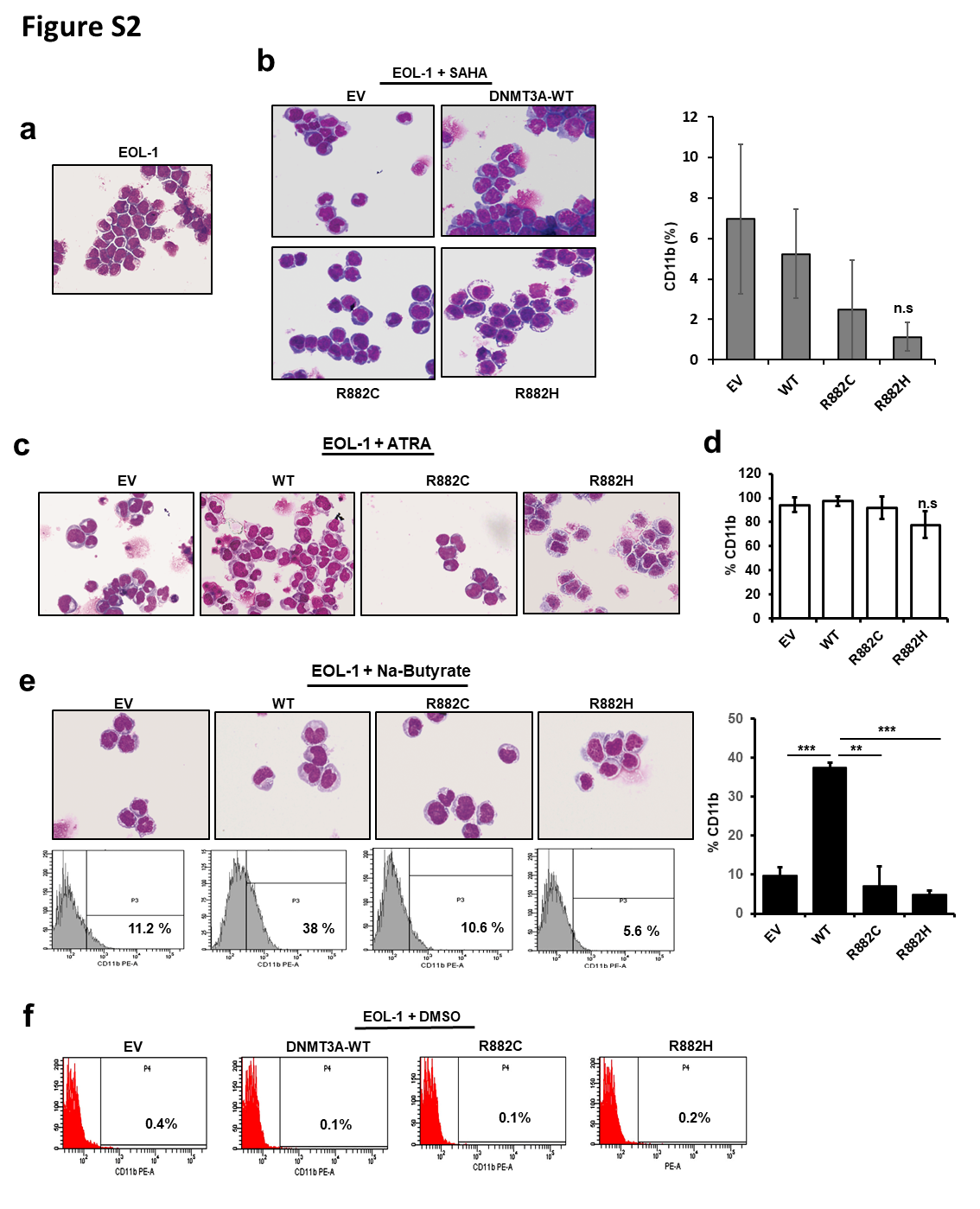

Supplement: Supplementary file 3 — Figure S2 [file 41389_2020_191_MOESM3_ESM.tif]

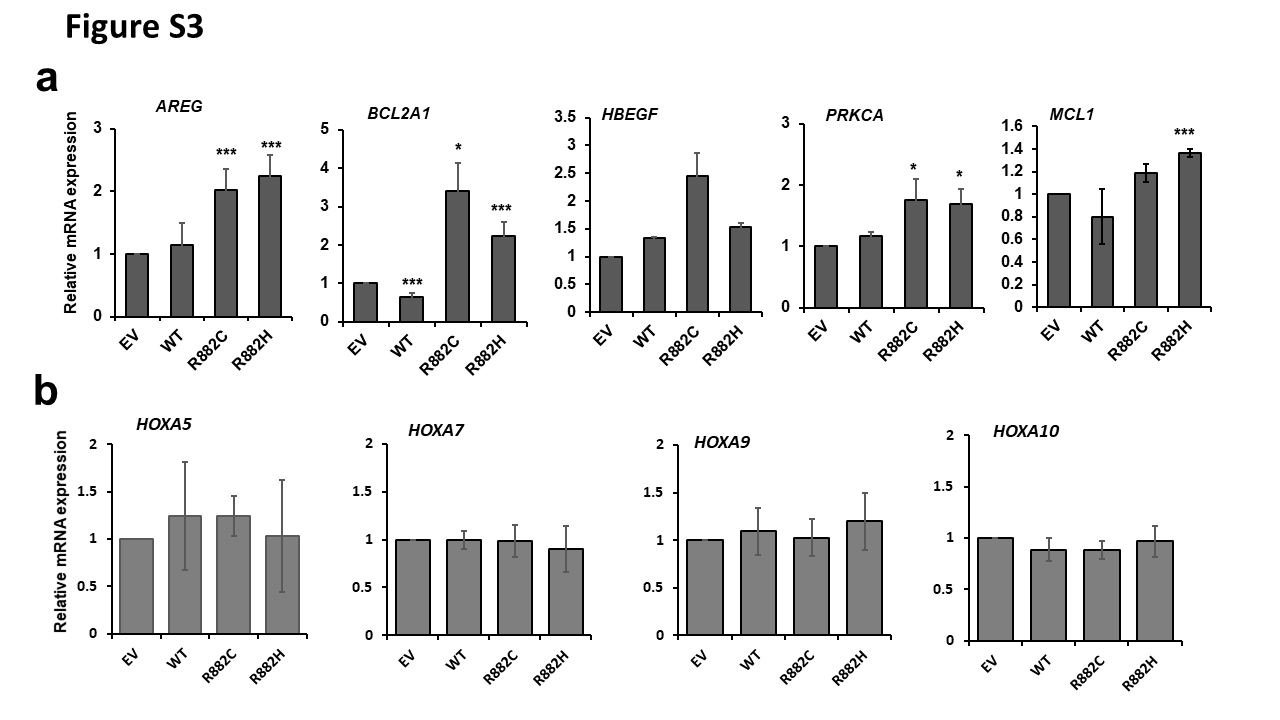

Supplement: Supplementary file 4 — Figure S3 [file 41389_2020_191_MOESM4_ESM.tif]

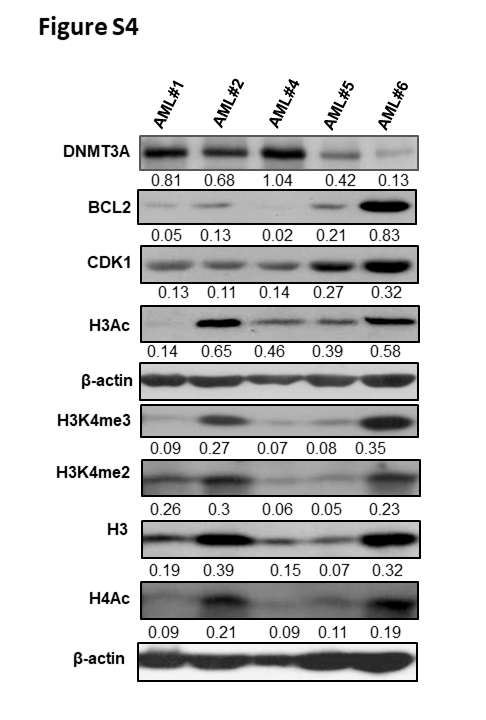

Supplement: Supplementary file 5 — Figure S4 [file 41389_2020_191_MOESM5_ESM.tif]

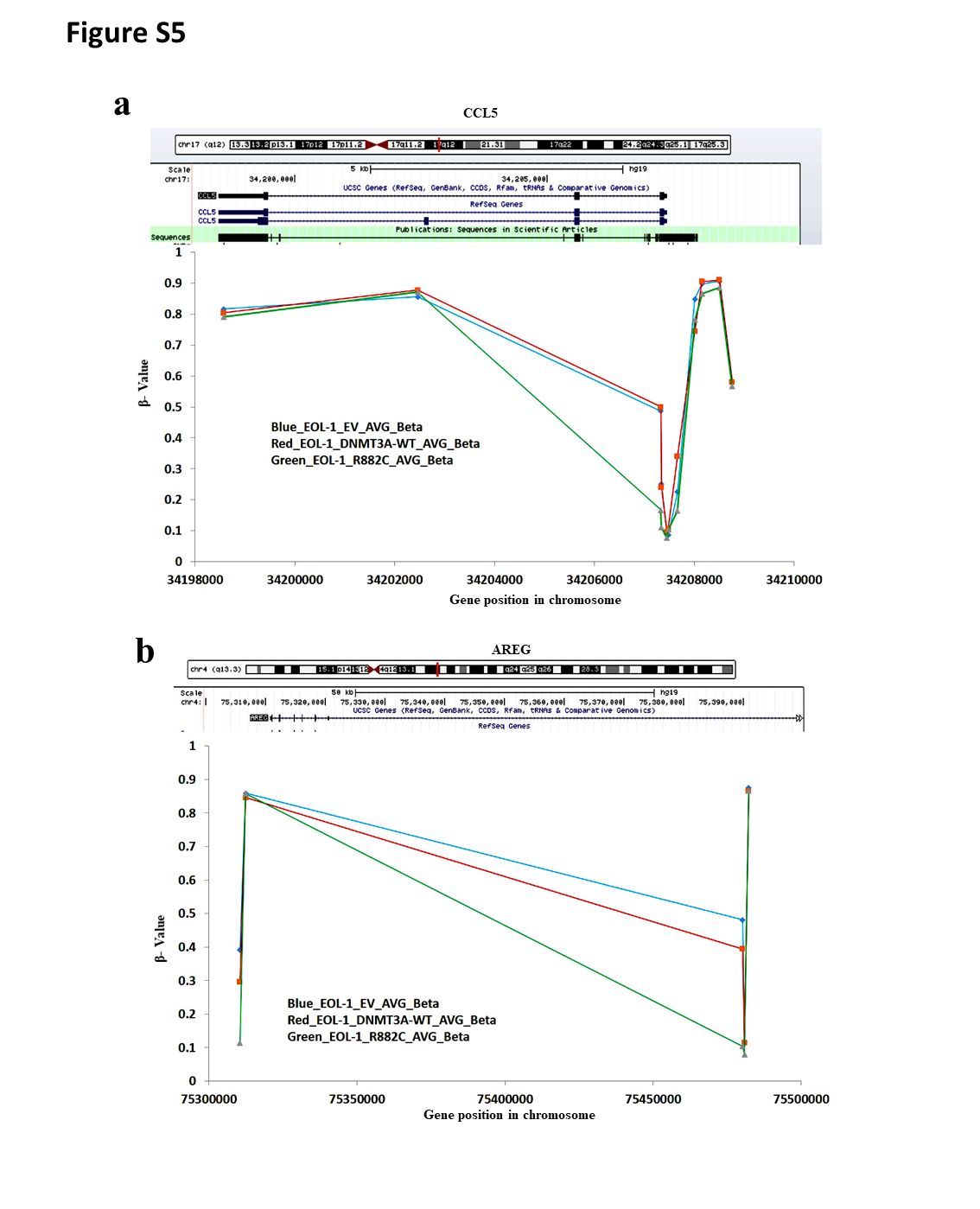

Supplement: Supplementary file 6 — Figure S5 [file 41389_2020_191_MOESM6_ESM.tif]

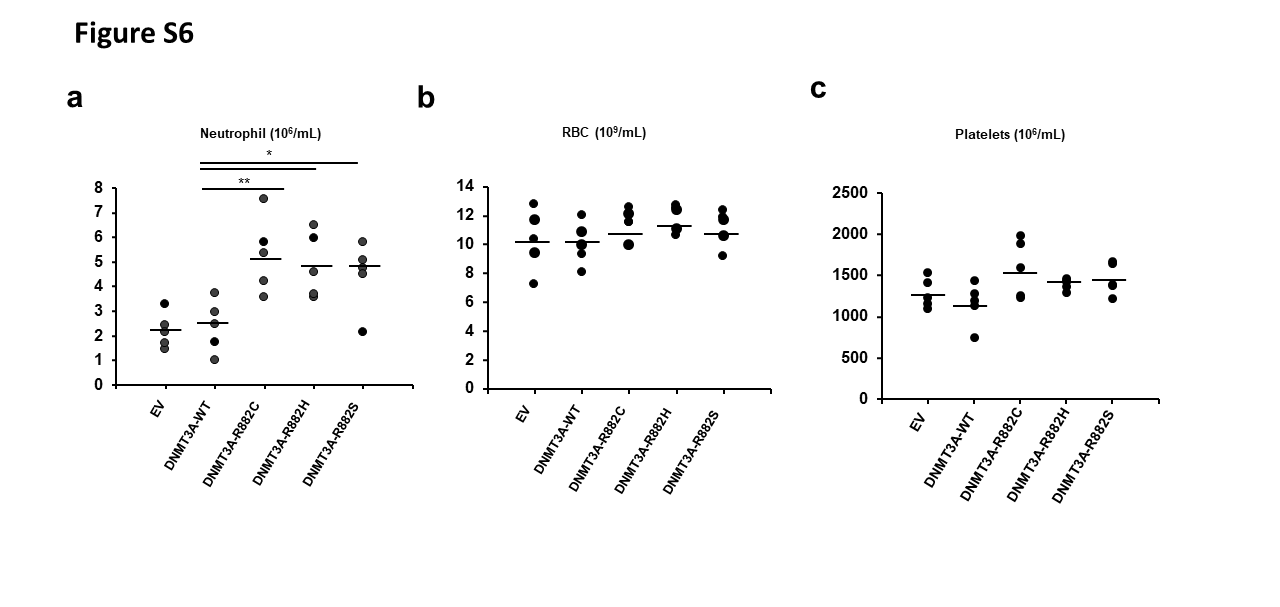

Supplement: Supplementary file 7 — Figure S6 [file 41389_2020_191_MOESM7_ESM.tif]

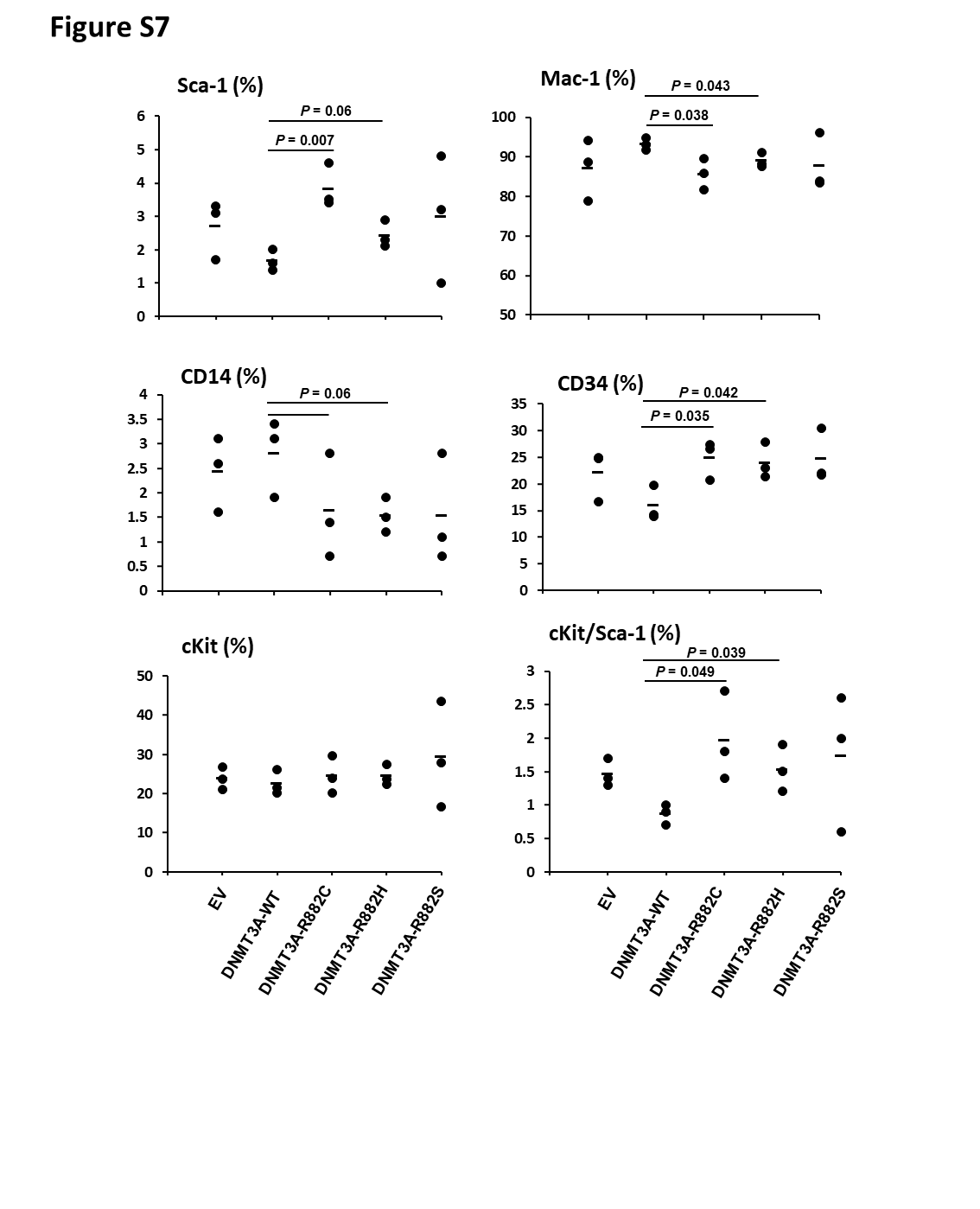

Supplement: Supplementary file 8 — Figure S7 [file 41389_2020_191_MOESM8_ESM.tif]
