# Supplementary material for: DNMT3A mutants provide proliferating advantage with augmentation of self-renewal activity in the pathogenesis of AML in KMT2A-PTD-positive leukemic cells
Source: Oncogenesis. 2020 Feb 3;9(2):7. doi: 10.1038/s41389-020-0191-6 (PMC6997180; doi:10.1038/s41389-020-0191-6)
Supplement: Supplementary file 9 — Table S1 [file 41389_2020_191_MOESM9_ESM.pdf]

Table S1. Characteristics of *KMT2A*-PTD with *DNMT3A*-WT/MT patient samples used for primary culture

Sample No	FAB	CEPBA	FLT3/ITD	NPM mutant	DNMT3A-WT/MT
F160523001-AML#1	M2	N	N	N	DNMT3A-WT
H161006004-AML#2	M0	N	N	N	DNMT3A-WT
F161121001-AML#3	M1	N	N	N	DNMT3A-WT
F170125001-AML#4	M4	N	N	N	DNMT3A-WT
F151214001-AML#5	M4	N	Y	N	DNMT3A-R882H
F120711001-AML#6	M2	N	N	N	DNMT3A-R882H
