# Supplementary material for: DNMT3A mutants provide proliferating advantage with augmentation of self-renewal activity in the pathogenesis of AML in KMT2A-PTD-positive leukemic cells
Source: Oncogenesis. 2020 Feb 3;9(2):7. doi: 10.1038/s41389-020-0191-6 (PMC6997180; doi:10.1038/s41389-020-0191-6)
Supplement: Supplementary file 10 — Table S2 [file 41389_2020_191_MOESM10_ESM.pdf]

Table S2. Characteristics of *KMT2A*-PTD positive patient samples used for microarray analyses

Sample No	FAB	FLT3/ITD	NPM mutant	DNMT3A mutant
GSM366163	M2	Y	N	N
GSM366165	M2	Y	N	N
GSM366166	M2	Y	N	N
GSM366172	M1	Y	N	N
GSM366164	M4	Y	N	Y
GSM366167	M2	Y	N	Y
GSM366171	M1	Y	N	Y

Y: DNMT3A-mutation, **N: DNMT3A-wild-type**
