# Supplementary material for: DNMT3A mutants provide proliferating advantage with augmentation of self-renewal activity in the pathogenesis of AML in KMT2A-PTD-positive leukemic cells
Source: Oncogenesis. 2020 Feb 3;9(2):7. doi: 10.1038/s41389-020-0191-6 (PMC6997180; doi:10.1038/s41389-020-0191-6)
Supplement: Supplementary file 11 — Table S3 [file 41389_2020_191_MOESM11_ESM.pdf]

Table S3. List of upregulated genes (>2 folds) in *KMT2A*-PTD/*DNMT3A*-MT vs *KMT2A*-PTD/*DNMT3A*-WT patient's samples matched with fingerprint genes

HSC	Naïve T-cell	Myeloid	Lymphoid	All Differentiated	Granulocyte	Monocytic	N. ER	NK cell	B-Cell
MEIS1	*ITK*	*ADPRH*	*LY9*	*CCL5*	*RAB20*	*CEBPD*	*E2F2*	*ID2*	*CRISP3*
FHL1	*IL7R*	*NFAM1*		*LILRB4*	*PTGS1*	*GCNT2*	*GALNT10*	*LRRC8C*	*CD180*
MPP7	*BCL11B*	*ENTPD1*		*POU2AF1*	*CPNE3*	*RETN*	*LMNA*	*TNFSF12*	*MYL4*
PTRF	*DDX3Y*	*BCL6*		*CD48*	*TBC1D8*	*NLRP1*	*OGFRL1*	*PRF1*	*NID1*
CALML4		*IL13RA1*			*CYBB*	*ADAM8*	*XPO7*	*SULF2*	*MTSS1*
GNAI1		*GLIPR2*				*SOCS3*	*KLF1*		*CCR6*
MMRN1		*PCNX*				*NFKBIZ*	*MFHAS1*		
STOX2		*RIN2*				*RGS2*	*ARHGEF12*		
FZD6		*APP*				*MSR1*	*SOX6*		
NR4A2		*CLEC5A*					*SLC22A4*		
PDK4		*TLR4*					*ARL4C*		
EMP1		*IL1RN*					*ANK1*		
HBEGF		*FGR*							
VNN1		*CRISPLD2*							
CD34		*HK3*							
		CD80							
		TREM1							
		SLC11A1							
		NCF1C							
		FCGR3A							
		CCR1							
