# Supplementary material for: DNMT3A mutants provide proliferating advantage with augmentation of self-renewal activity in the pathogenesis of AML in KMT2A-PTD-positive leukemic cells
Source: Oncogenesis. 2020 Feb 3;9(2):7. doi: 10.1038/s41389-020-0191-6 (PMC6997180; doi:10.1038/s41389-020-0191-6)
Supplement: Supplementary file 12 — Table S4 [file 41389_2020_191_MOESM12_ESM.pdf]

Table S4. List of primer sets for quantitative RT-PCR to check mRNA expression of different genes

Gene	Forward primer	Reverse primer
HOXA5	GGCTACAATGGCATGGATCT	CTGAGGAGAGTGCGTGGAC
HOXA7	ACTTCAACCGCTACCTGACG	CAGTCGGACCTTCGTCCTTA
HOXA9	CCACGCTTGACACTCACACT	CGCTCTCATTCTCAGCATTG
HOXA10	GGATTCCCTGGGCAATTCCA	CTAATCTCTAGGCGCCGCTC
HOXB2	ACACGCAGCTGCTGGAACT	TCTGCCGCTTGTGCTTCAT
HOXB3	AGGAGCCAGAGAAGGTGGTA	GATGTGGATTCCTTTCCGAT
HOXB4	CACCCTCTGACTGCCAGATA	GTGGGTGAGCAGTCATTCTG
HOXB5	CCAATTTCACCGAAATAGACG	GGGAATATTTGCGGAGTCTG
HOXB6	GCGAGACAGAAGAGCAGAAG	CAGCGTCTGGTAACGTGTGT
HOXB8	TTATTATGACTGCGGCTTCG	GTAGGGAGCCGTGGACAG
BCL2A1	ATTGCCCCGGATGTGGATA	AAAAGTCATCCAGCCAGATTTAGG
HLX	CCCTATGCTGTGCTCACGAA	GCGACCATGAACGCTTCCT
AREG	CGGCTCAGGCCATTATGC	GGTCCCCAGAAAATGGTTCA
HBEGF	GGAGCTGACTGTTCTTGGTAACTG	TCCCCACCTCCAACCTTCTC
PRKCA	CCAGTGGATGGTACAAGTTGCTTA	CCTTCCGGAATGGGTACGT
MCL1	GGAAGAGTGCTCCCCATTGA	TTGTTGCTGAAACTGAACTTTGC
