# Supplementary material for: DNMT3A mutants provide proliferating advantage with augmentation of self-renewal activity in the pathogenesis of AML in KMT2A-PTD-positive leukemic cells
Source: Oncogenesis. 2020 Feb 3;9(2):7. doi: 10.1038/s41389-020-0191-6 (PMC6997180; doi:10.1038/s41389-020-0191-6)
Supplement: Supplementary file 13 — Table S5 [file 41389_2020_191_MOESM13_ESM.pdf]

**Table S5. List of primer pairs for Chromatin-immunoprecipitated (ChIP) quantitative RT-PCR to check the enrichment of H4Ac at different *HOXB* promoter regions**

| Gene | Forward primer | Reverse primer |
|---|---|---|
| ***HOXB2*** | GTTTGTGTTGGGACCTAAACTCTTT | GACCTGCCCATCTCTATTTCC |
| ***HOXB3*** | GGTGGTCCAATTTAGATAGCAGAG | ACTTAAACCACAGCTGAAGAAGTTG |
| ***HOXB4*** | CCCAGTGTATATTTTAAGCACCCTA | AATGAAACATTTTCCCCCATTAG |
| ***HOXB7*** | CCTATTTTCTGTGTCTTCCTAAAGC | TGACAGTGTGTATGTTTGATGTCAG |
