# Supplementary material for: DNMT3A mutants provide proliferating advantage with augmentation of self-renewal activity in the pathogenesis of AML in KMT2A-PTD-positive leukemic cells
Source: Oncogenesis. 2020 Feb 3;9(2):7. doi: 10.1038/s41389-020-0191-6 (PMC6997180; doi:10.1038/s41389-020-0191-6)
Supplement: Supplementary file 14 — Dataset S1 [file 41389_2020_191_MOESM14_ESM.pdf]

List of upregulated (>2 folds) genes in KMT2A-PTD/DNMT3A-MT AML cells compared to KMT2A-PTD/DNMT3A-WT cells

Gene Symbol	Gene Title	Fold-Change
AREG	amphiregulin	28.2015
MPEG1	macrophage expressed 1	14.2533
MRC1	mannose receptor, C type 1	13.1809
MAFB	v-maf musculoaponeurotic fibrosarcoma oncogene homolog B (avian)	11.8836
VCAN	versican	11.626
S100A9	S100 calcium binding protein A9	11.063
KCTD12	potassium channel tetramerisation domain containing 12	10.8835
CD14	CD14 molecule	10.8539
C5AR1	complement component 5a receptor 1	10.3146
AREGB	Amphiregulin B	9.90513
CD86	CD86 molecule	9.49179
LGALS2	lectin, galactoside-binding, soluble, 2	9.15109
SERPINA1	serpin peptidase inhibitor, clade A (alpha-1 antiproteinase, antitrypsin)	9.1363
ALOX5	arachidonate 5-lipoxygenase	8.89379
LGALS3	lectin, galactoside-binding, soluble, 3	8.71032
C1QA	complement component 1, q subcomponent, A chain	8.46087
FGL2	fibrinogen-like 2	8.44722
TBC1D9	TBC1 domain family, member 9 (with GRAM domain)	8.42051
TLR8	toll-like receptor 8	8.29364
S100A8	S100 calcium binding protein A8	8.23478
PRKCA	protein kinase C, alpha	8.17017
CD24	CD24 molecule	8.09183
HLA-DQB1	major histocompatibility complex, class II, DQ beta 1	8.05753
PMP22	peripheral myelin protein 22	7.73296
CD36	CD36 molecule (thrombospondin receptor)	7.55307
HBM	hemoglobin, mu	7.50036
SGK1	serum/glucocorticoid regulated kinase 1	7.34263
SULF2	sulfatase 2	7.31323
NCF2	neutrophil cytosolic factor 2	7.28145
GPR109B	G protein-coupled receptor 109B	7.22532
ARL4C	ADP-ribosylation factor-like 4C	7.21945
ANXA5	annexin A5	7.19478
HBEGF	heparin-binding EGF-like growth factor	7.18908
CX3CR1	chemokine (C-X3-C motif) receptor 1	6.89832
BLNK	B-cell linker	6.88077
MYOF	myoferlin	6.87193
NRGN	neurogranin (protein kinase C substrate, RC3)	6.81458
CKAP4	cytoskeleton-associated protein 4	6.75327
LY96	lymphocyte antigen 96	6.74875
CD1D	CD1d molecule	6.67937
IFI30	interferon, gamma-inducible protein 30	6.56767
SH3BP5	SH3-domain binding protein 5 (BTK-associated)	6.44658

LDLRAD3	low density lipoprotein receptor class A domain containing 3	6.34757
EREG	epiregulin	6.27398
CPEB4	cytoplasmic polyadenylation element binding protein 4	6.26079
NPL	N-acetylneuraminate pyruvate lyase (dihydrodipicolinate synthase)	6.21955
LY86	lymphocyte antigen 86	6.17822
SERPINB9	serpin peptidase inhibitor, clade B (ovalbumin), member 9	6.15572
DDIT4	DNA-damage-inducible transcript 4	6.14432
MPP7	membrane protein, palmitoylated 7 (MAGUK p55 subfamily member 7)	6.09364
CD52	CD52 molecule	6.0307
CCR1	chemokine (C-C motif) receptor 1	6.01009
SIAE	sialic acid acetylesterase	5.96455
SRGAP2P1	SLIT-ROBO Rho GTPase activating protein 2 pseudogene 1	5.94958
KYNU	kynureninase (L-kynurenine hydrolase)	5.86325
LYZ	lysozyme	5.70248
LILRB2	ocyte immunoglobulin-like receptor, subfamily B (with TM and ITIM doma	5.67502
JAZF1	JAZF zinc finger 1	5.62182
CEBPD	CCAAT/enhancer binding protein (C/EBP), delta	5.60178
NCF1	neutrophil cytosolic factor 1	5.58005
FGD4	FYVE, RhoGEF and PH domain containing 4	5.55771
HLA-DQA1	major histocompatibility complex, class II, DQ alpha 1	5.53383
KIAA0146	KIAA0146	5.52016
LGALS3BP	lectin, galactoside-binding, soluble, 3 binding protein	5.50753
NID1	nidogen 1	5.48898
CLEC4A	C-type lectin domain family 4, member A	5.48803
BCL2A1	BCL2-related protein A1	5.44743
CECR1	cat eye syndrome chromosome region, candidate 1	5.40191
TPM1	tropomyosin 1 (alpha)	5.39928
SLC25A37	solute carrier family 25, member 37	5.38575
CTSH	cathepsin H	5.37771
MCL1	myeloid cell leukemia sequence 1 (BCL2-related)	5.33297
SELENBP1	selenium binding protein 1	5.32842
IGF2R	insulin-like growth factor 2 receptor	5.321
ITGAM	integrin, alpha M (complement component 3 receptor 3 subunit)	5.29494
IL10RA	interleukin 10 receptor, alpha	5.28888
CXCR4	chemokine (C-X-C motif) receptor 4	5.28272
BASP1	brain abundant, membrane attached signal protein 1	5.247
CD52	CD52 molecule	5.20447
IGSF6	immunoglobulin superfamily, member 6	5.19405
GBP1	guanylate binding protein 1, interferon-inducible, 67kDa	5.16799
MS4A1	membrane-spanning 4-domains, subfamily A, member 1	5.10368
FCGR3A	Fc fragment of IgG,	5.09212
PLBD1	phospholipase B domain containing 1	5.0771
NCF1C	neutrophil cytosolic factor 1C pseudogene	5.06844
DDX3Y	DEAD (Asp-Glu-Ala-Asp) box polypeptide 3, Y-linked	5.06724
MS4A7	membrane-spanning 4-domains, subfamily A, member 7	4.99884
IRF8	interferon regulatory factor 8	4.94841

SLC11A1	solute carrier family 11 (proton-coupled divalent metal ion transporters)	4.93578
TMEM49	transmembrane protein 49	4.92421
SGMS2	sphingomyelin synthase 2	4.9153
CLEC7A	C-type lectin domain family 7, member A	4.90767
CCR2	chemokine (C-C motif) receptor 2	4.89143
FBP1	fructose-1,6-bisphosphatase 1	4.89089
RPS4Y1	ribosomal protein S4, Y-linked 1	4.88379
ZBTB38	zinc finger and BTB domain containing 38	4.85385
PTPRC	protein tyrosine phosphatase, receptor type, C	4.82319
EIF1AY	eukaryotic translation initiation factor 1A, Y-linked	4.80379
RBM47	RNA binding motif protein 47	4.7805
TGFBI	transforming growth factor, beta-induced, 68kDa	4.73209
SLC7A7	lute carrier family 7 (cationic amino acid transporter, y+ system), membe	4.72133
FPR1	formyl peptide receptor 1	4.68393
IER3	immediate early response 3	4.66607
EPB42	erythrocyte membrane protein band 4.2	4.64474
ALDH2	aldehyde dehydrogenase 2 family (mitochondrial)	4.61563
TNS1	tensin 1	4.59097
ABHD5	abhydrolase domain containing 5	4.5867
CYTIP	cytohesin 1 interacting protein	4.55135
LONRF2	LON peptidase N-terminal domain and ring finger 2	4.5349
TREM1	triggering receptor expressed on myeloid cells 1	4.51979
PTPRJ	protein tyrosine phosphatase, receptor type, J	4.51256
NOD2	nucleotide-binding oligomerization domain containing 2	4.46525
ZFP36	zinc finger protein 36, C3H type, homolog (mouse)	4.45879
HLA-DQA1	major histocompatibility complex, class II, DQ alpha 1	4.44423
CRISP3	cysteine-rich secretory protein 3	4.44117
CD34	CD34 molecule	4.38522
FOSL2	FOS-like antigen 2	4.36599
MS4A6A	membrane-spanning 4-domains, subfamily A, member 6A	4.34302
CYBB	cytochrome b-245, beta polypeptide	4.34287
FCER1G	Fc fragment of IgE, high affinity I, receptor for; gamma polypeptide	4.31134
HLA-DQA2	major histocompatibility complex, class II, DQ alpha 1	4.31096
FAM198B	family with sequence similarity 198, member B	4.29735
RAB31	RAB31, member RAS oncogene family	4.29576
LPHN3	latrophilin 3	4.28824
PELI1	pellino homolog 1 (Drosophila)	4.27103
SLC16A6	solute carrier family 16, member 6 (monocarboxylic acid transporter 7)	4.25821
TNFRSF1B	tumor necrosis factor receptor superfamily, member 1B	4.25044
SORT1	sortilin 1	4.21351
STX11	syntaxin 11	4.20202
AHSP	alpha hemoglobin stabilizing protein	4.2003
CD80	CD80 molecule	4.19957
MS4A4A	membrane-spanning 4-domains, subfamily A, member 4	4.18201
VNN1	vanin 1	4.1564
TLR5	toll-like receptor 5	4.15197

CD93	CD93 molecule	4.14856
SCPEP1	serine carboxypeptidase 1	4.14406
HSPA6	heat shock 70kDa protein 6 (HSP70B')	4.13519
TNFAIP3	tumor necrosis factor, alpha-induced protein 3	4.1177
CDC42EP3	CDC42 effector protein (Rho GTPase binding) 3	4.11165
CHST15	carbohydrate (N-acetylgalactosamine 4-sulfate 6-O) sulfotransferase 15	4.09758
PHLDA1	pleckstrin homology-like domain, family A, member 1	4.09483
ADCY9	adenylate cyclase 9	4.09267
MICAL2	crotubule associated monoxygenase, calponin and LIM domain containin	4.08092
BACH2	BTB and CNC homology 1	4.06257
SAMSN1	SAM domain, SH3 domain and nuclear localization signals 1	4.0565
PF4	platelet factor 4	4.0376
HIP1	huntingtin interacting protein 1	4.01265
HLA-DPA1	major histocompatibility complex, class II, DP alpha 1	4.00169
PRKAR2B	protein kinase, cAMP-dependent, regulatory, type II, beta	3.99469
ANKRD9	ankyrin repeat domain 9	3.96604
CD163	CD163 molecule	3.95087
HCP5	HLA complex P5	3.95086
SV2B	synaptic vesicle glycoprotein 2B	3.95033
IL15	interleukin 15	3.94757
S100A12	S100 calcium binding protein A12	3.9303
CSF2RA	colony stimulating factor 2 receptor, alpha	3.91292
MARCH1	membrane-associated ring finger (C3HC4) 1	3.90045
FLVCR2	feline leukemia virus subgroup C cellular receptor family, member 2	3.87937
HK3	hexokinase 3 (white cell)	3.87875
IL7R	interleukin 7 receptor	3.87786
GCNT2	glucosaminyl (N-acetyl) transferase 2, I-branching enzyme (I blood group)	3.87195
LOXL1	lysyl oxidase-like 1	3.86003
FCN1	ficolin (collagen/fibrinogen domain containing) 1	3.85982
ZNF185	zinc finger protein 185 (LIM domain)	3.85852
LRRK2	leucine-rich repeat kinase 2	3.85574
EPB41L3	erythrocyte membrane protein band 4.1-like 3	3.83749
PAPSS2	3'-phosphoadenosine 5'-phosphosulfate synthase 2	3.83662
PI15	peptidase inhibitor 15	3.83428
CR1	complement component (3b/4b) receptor 1 (Knops blood group)	3.82958
CD74	CD74 molecule	3.8239
BCL6	B-cell CLL/lymphoma 6	3.8062
NAMPT	nicotinamide phosphoribosyltransferase	3.80359
THBS1	thrombospondin 1	3.80011
IL1R1	interleukin 1 receptor, type I	3.79046
TUBB2A	tubulin, beta 2A	3.78883
KDM5D	lysine (K)-specific demethylase 5D	3.77797
HLA-DPB1	major histocompatibility complex, class II, DP beta 1	3.77706
PDE4B	phosphodiesterase 4B, cAMP-specific	3.77293
PTPRE	protein tyrosine phosphatase, receptor type, E	3.75213
OSCAR	osteoclast associated, immunoglobulin-like receptor	3.75177

ALDH1A1	aldehyde dehydrogenase 1 family, member A1	3.74304
RETN	resistin	3.74241
FHL1	four and a half LIM domains 1	3.74067
CRISPLD2	cysteine-rich secretory protein LCCL domain containing 2	3.72342
LRRFIP1	leucine rich repeat (in FLII) interacting protein 1	3.70894
HNRNPU	ıeterogeneous nuclear ribonucleoprotein U (scaffold attachment factor A	3.69503
C1orf38	chromosome 1 open reading frame 38	3.68873
GPCPD1	glycerophosphocholine phosphodiesterase GDE1 homolog (S. cerevisiae)	3.68369
CDA	cytidine deaminase	3.67552
ZNF503	zinc finger protein 503	3.67188
FOXF1	forkhead box F1	3.65103
BCL11B	B-cell CLL/lymphoma 11B (zinc finger protein)	3.64509
RASSF4	Ras association (RalGDS/AF-6) domain family member 4	3.63521
NLRP3	NLR family, pyrin domain containing 3	3.61586
SGMS1	sphingomyelin synthase 1	3.58955
TM6SF1	transmembrane 6 superfamily member 1	3.58857
ARHGAP21	Rho GTPase activating protein 21	3.58628
CCL5	chemokine (C-C motif) ligand 5	3.58409
HEMGN	hemogen	3.57799
CYorf15B	chromosome Y open reading frame 15B	3.57777
CD48	CD48 molecule	3.57695
DAAM1	Dishevelled associated activator of morphogenesis 1	3.57489
ID1	inhibitor of DNA binding 1, dominant negative helix-loop-helix protein	3.57334
LILRB1	ɔcyte immunoglobulin-like receptor, subfamily B (with TM and ITIM doma	3.5639
SLC24A4	ɩolute carrier family 24 (sodium/potassium/calcium exchanger), member	3.56302
GYPA	glycophorin A (MNS blood group)	3.56102
MLLT4	oid/lymphoid or mixed-lineage leukemia (trithorax homolog, Drosophila)	3.55595
RAB7L1	RAB7, member RAS oncogene family-like 1	3.54912
ALAS2	aminolevulinate, delta-, synthase 2	3.54879
NLRP1	NLR family, pyrin domain containing 1	3.5481
FAM108C1	family with sequence similarity 108, member C1	3.54467
HLA-DRB1	major histocompatibility complex, class II, DR beta 1	3.53898
CD86	CD86 molecule	3.53459
F2RL1	coagulation factor II (thrombin) receptor-like 1	3.53106
RHAG	Rh-associated glycoprotein	3.52147
NACC2	NACC family member 2, BEN and BTB (POZ) domain containing	3.51263
VNN2	vanin 2	3.50874
LOC400931	hypothetical LOC400931	3.50276
C10orf128	chromosome 10 open reading frame 128	3.50056
FGR	Gardner-Rasheed feline sarcoma viral (v-fgr) oncogene homolog	3.49877
FCER1A	Fc fragment of IgE, high affinity I, receptor for; alpha polypeptide	3.49427
FAM65C	family with sequence similarity 65, member C	3.48176
HLA-DMB	major histocompatibility complex, class II, DM beta	3.47789
RNF144B	ring finger protein 144B	3.4712
MMP19	matrix metallopeptidase 19	3.46331
GPR183	G protein-coupled receptor 183	3.45952

NEURL1B	neuralized homolog 1B (Drosophila)	3.45651
MARCKS	myristoylated alanine-rich protein kinase C substrate	3.45492
TYMP	thymidine phosphorylase	3.44798
PMCH	pro-melanin-concentrating hormone	3.44787
AGPAT9	1-acylglycerol-3-phosphate O-acyltransferase 9	3.44661
ENPP4	ectonucleotide pyrophosphatase/phosphodiesterase 4 (putative function	3.4464
CH25H	cholesterol 25-hydroxylase	3.42943
SYTL3	synaptotagmin-like 3	3.4232
FGD2	FYVE, RhoGEF and PH domain containing 2	3.41762
GABBR1	gamma-aminobutyric acid (GABA) B receptor, 1	3.41288
NEDD9	neural precursor cell expressed, developmentally down-regulated 9	3.41281
POU2AF1	POU class 2 associating factor 1	3.40663
RTN1	reticulon 1	3.40213
DNTT	deoxynucleotidyltransferase, terminal	3.3837
YPEL4	yippee-like 4 (Drosophila)	3.38143
LDLR	low density lipoprotein receptor	3.37912
MIR21	microRNA 21	3.37694
SLC2A3	solute carrier family 2 (facilitated glucose transporter), member 3	3.37133
PDZK1IP1	PDZK1 interacting protein 1	3.358
RHOU	ras homolog gene family, member U	3.35504
PTGFR	prostaglandin F receptor (FP)	3.34608
LINS1	lines homolog 1 (Drosophila)	3.34356
GIMAP8	GTPase, IMAP family member 8	3.33733
CYTL1	cytokine-like 1	3.33025
HMOX1	heme oxygenase (decycling) 1	3.32912
SH3TC1	SH3 domain and tetratricopeptide repeats 1	3.32645
SLC6A8	solute carrier family 6 (neurotransmitter transporter, creatine), member 8	3.3163
METRNL	meteorin, glial cell differentiation regulator-like	3.31134
CACNA2D3	calcium channel, voltage-dependent, alpha 2/delta subunit 3	3.30832
DHRS9	dehydrogenase/reductase (SDR family) member 9	3.30623
MTMR11	myotubularin related protein 11	3.29031
NEAT1	nuclear paraspeckle assembly transcript 1 (non-protein coding)	3.27644
LRRC25	leucine rich repeat containing 25	3.26791
PTAFR	platelet-activating factor receptor	3.24468
AMICA1	adhesion molecule, interacts with CXADR antigen 1	3.2402
SLC2A14	solute carrier family 2 (facilitated glucose transporter), member 14	3.23285
CDKN1A	cyclin-dependent kinase inhibitor 1A (p21, Cip1)	3.22588
CD109	CD109 molecule	3.2212
ITGB2	integrin, beta 2 (complement component 3 receptor 3 and 4 subunit)	3.20963
MXD1	MAX dimerization protein 1	3.20904
SLC8A1	solute carrier family 8 (sodium/calcium exchanger), member 1	3.20815
TUBB1	tubulin, beta 1	3.20685
SERPINB6	serpin peptidase inhibitor, clade B (ovalbumin), member 6	3.20318
GNLY	granulysin	3.18226
PER1	period homolog 1 (Drosophila)	3.16823
RORA	RAR-related orphan receptor A	3.1615

LMNA	lamin A/C	3.14351
GZMA	ınzyme A (granzyme 1, cytotoxic T-lymphocyte-associated serine esterase	3.1403
KLF9	Kruppel-like factor 9	3.12754
LILRB4	›cyte immunoglobulin-like receptor, subfamily B (with TM and ITIM doma	3.11825
GYPB	glycophorin B (MNS blood group)	3.11684
ITGAL	grin, alpha L (antigen CD11A (p180), lymphocyte function-associated anti	3.11322
TBC1D8	TBC1 domain family, member 8 (with GRAM domain)	3.10753
SFRS11	Splicing factor, arginine/serine-rich 11	3.09943
C11orf9	chromosome 11 open reading frame 9	3.08789
SIDT1	SID1 transmembrane family, member 1	3.06758
SLFN13	schlafen family member 13	3.06594
SLC4A1	e carrier family 4, anion exchanger, member 1 (erythrocyte membrane pr	3.05425
AIF1L	allograft inflammatory factor 1-like	3.05335
DPP4	dipeptidyl-peptidase 4	3.05181
LILRA2	›cyte immunoglobulin-like receptor, subfamily A (with TM domain), mem	3.03341
CDC42EP3	CDC42 effector protein (Rho GTPase binding) 3	3.0289
SGSH	N-sulfoglucosamine sulfohydrolase	3.02635
FRMD3	FERM domain containing 3	3.02558
GIMAP4	GTPase, IMAP family member 4	3.0238
IL1RN	interleukin 1 receptor antagonist	3.02126
GAS6	growth arrest-specific 6	3.01945
PSAP	prosaposin	3.01938
BCL2L11	BCL2-like 11 (apoptosis facilitator)	3.01857
SRGAP2	SLIT-ROBO Rho GTPase activating protein 2	3.01245
GOLGA8A	golgin A8 family, member A	3.00823
MALAT1	etastasis associated lung adenocarcinoma transcript 1 (non-protein codin	2.99392
HLA-DRB1	Major histocompatibility complex, class II, DR beta 1	2.99211
CCR7	chemokine (C-C motif) receptor 7	2.98959
RNF182	ring finger protein 182	2.98805
RAP1GAP	RAP1 GTPase activating protein	2.98577
LOC284757	hypothetical protein LOC284757	2.98173
NPR3	natriuretic peptide receptor C/guanylate cyclase C	2.97845
DOCK10	dedicator of cytokinesis 10	2.97836
MEGF9	multiple EGF-like-domains 9	2.97772
ARHGEF12	Rho guanine nucleotide exchange factor (GEF) 12	2.97429
IFNGR1	interferon gamma receptor 1	2.97277
GGT1	gamma-glutamyltransferase 1	2.96768
IL6ST	interleukin 6 signal transducer (gp130, oncostatin M receptor)	2.96692
EMP1	epithelial membrane protein 1	2.96568
RGS10	regulator of G-protein signaling 10	2.96362
DACH1	dachshund homolog 1 (Drosophila)	2.95908
HNRPLL	heterogeneous nuclear ribonucleoprotein L-like	2.94966
LOC284837	hypothetical LOC284837	2.9392
PKIB	protein kinase (cAMP-dependent, catalytic) inhibitor beta	2.93329
BLVRB	biliverdin reductase B (flavin reductase (NADPH))	2.92884
ALOX5AP	arachidonate 5-lipoxygenase-activating protein	2.925

PLEC	plectin	2.92251
GIMAP7	GTPase, IMAP family member 7	2.91961
RNASE4	ribonuclease, RNase A family, 4	2.91573
PADI4	peptidyl arginine deiminase, type IV	2.91385
TMCC2	transmembrane and coiled-coil domain family 2	2.9118
PSTPIP1	proline-serine-threonine phosphatase interacting protein 1	2.90236
FAM132B	family with sequence similarity 132, member B	2.8985
SOCS1	suppressor of cytokine signaling 1	2.89388
CTSL1	cathepsin L1	2.88813
CLEC3B	C-type lectin domain family 3, member B	2.88343
CTSS	cathepsin S	2.88139
LOC728392	hypothetical protein LOC728392	2.88089
KBTBD8	kelch repeat and BTB (POZ) domain containing 8	2.87038
KLF9	Kruppel-like factor 9	2.86337
PTH2R	parathyroid hormone 2 receptor	2.85493
DDX17	DEAD (Asp-Glu-Ala-Asp) box polypeptide 17	2.85175
RXFP1	relaxin/insulin-like family peptide receptor 1	2.84816
PLAUR	plasminogen activator, urokinase receptor	2.8395
C6orf192	chromosome 6 open reading frame 192	2.82912
RGS1	regulator of G-protein signaling 1	2.82731
EPS8	epidermal growth factor receptor pathway substrate 8	2.82534
AQP1	aquaporin 1 (Colton blood group)	2.82451
SOX6	SRY (sex determining region Y)-box 6	2.82393
CPNE3	copine III	2.81532
SECTM1	secreted and transmembrane 1	2.81306
LEF1	lymphoid enhancer-binding factor 1	2.80536
ADAP2	ArfGAP with dual PH domains 2	2.80511
RXRA	retinoid X receptor, alpha	2.80484
LYST	lysosomal trafficking regulator	2.79267
MFHAS1	malignant fibrous histiocytoma amplified sequence 1	2.79211
MRPL43	mitochondrial ribosomal protein L43	2.78507
TBL1X	transducin (beta)-like 1X-linked	2.78241
SLC15A3	solute carrier family 15, member 3	2.77893
PDK4	pyruvate dehydrogenase kinase, isozyme 4	2.77494
THBD	thrombomodulin	2.77167
RASGRP1	RAS guanyl releasing protein 1 (calcium and DAG-regulated)	2.77073
FAM46C	family with sequence similarity 46, member C	2.76916
SIK1	salt-inducible kinase 1	2.7681
USP9Y	ubiquitin specific peptidase 9, Y-linked	2.76774
TMEM204	transmembrane protein 204	2.76443
C17orf91	chromosome 17 open reading frame 91	2.76372
ASAP1IT1	ASAP1 intronic transcript 1 (non-protein coding)	2.76309
KLF11	Kruppel-like factor 11	2.76068
CCL5	chemokine (C-C motif) ligand 5	2.75466
CSN1S1	casein alpha s1	2.75125
BLVRA	biliverdin reductase A	2.75093

ZC3H12A	zinc finger CCCH-type containing 12A	2.74835
NR4A2	nuclear receptor subfamily 4, group A, member 2	2.74438
TRIM10	tripartite motif-containing 10	2.74386
CA1	carbonic anhydrase I	2.7429
CXCL3	chemokine (C-X-C motif) ligand 3	2.73861
PPBP	pro-platelet basic protein (chemokine (C-X-C motif) ligand 7)	2.72882
PI4K2A	phosphatidylinositol 4-kinase type 2 alpha	2.72073
NCEH1	neutral cholesterol ester hydrolase 1	2.71966
ANK1	ankyrin 1, erythrocytic	2.71844
FTH1	ferritin, heavy polypeptide 1	2.71022
CTSB	cathepsin B	2.70328
PAX6	paired box 6	2.68856
GPX3	glutathione peroxidase 3 (plasma)	2.68826
LOC100286909	Hypothetical protein LOC100286909	2.67959
PLA2G16	phospholipase A2, group XVI	2.6784
HLA-DRA	major histocompatibility complex, class II, DR alpha	2.67825
SRGN	serglycin	2.67814
RASGEF1B	RasGEF domain family, member 1B	2.67798
KLRB1	killer cell lectin-like receptor subfamily B, member 1	2.67563
ADAM8	ADAM metallopeptidase domain 8	2.67278
LOC285812	hypothetical protein LOC285812	2.67257
CEBPB	CCAAT/enhancer binding protein (C/EBP), beta	2.66855
IL1R2	interleukin 1 receptor, type II	2.66823
SYNJ2	synaptojanin 2	2.66682
LILRB3	ocyte immunoglobulin-like receptor, subfamily B (with TM and ITIM doma	2.66267
FNIP2	folliculin interacting protein 2	2.6589
PRF1	perforin 1 (pore forming protein)	2.65758
C1orf162	chromosome 1 open reading frame 162	2.65476
DLG5	discs, large homolog 5 (Drosophila)	2.65383
CD83	CD83 molecule	2.65165
TLR4	toll-like receptor 4	2.6444
EML4	echinoderm microtubule associated protein like 4	2.64376
RASSF6	Ras association (RalGDS/AF-6) domain family member 6	2.6434
MGEA5	Meningioma expressed antigen 5 (hyaluronidase)	2.64159
IQSEC1	IQ motif and Sec7 domain 1	2.64114
GNG2	guanine nucleotide binding protein (G protein), gamma 2	2.64041
CRTAM	cytotoxic and regulatory T cell molecule	2.63463
SEC14L4	SEC14-like 4 (S. cerevisiae)	2.63381
MYH10	myosin, heavy chain 10, non-muscle	2.63009
PRR5L	proline rich 5 like	2.628
MX1	myxovirus (influenza virus) resistance 1, interferon-inducible protein p78	2.62561
METTL7B	methyltransferase like 7B	2.62271
ADAMTSL4	ADAMTS-like 4	2.62267
LATS2	LATS, large tumor suppressor, homolog 2 (Drosophila)	2.62233
SVIL	supervillin	2.6211
COTL1	coactosin-like 1 (Dictyostelium)	2.62002

ANKH	ankylosis, progressive homolog (mouse)	2.61943
SLC22A4	lute carrier family 22 (organic cation/ergothioneine transporter), membe	2.6101
AKAP13	A kinase (PRKA) anchor protein 13	2.60988
INO80D	INO80 complex subunit D	2.60831
SIRPB1	signal-regulatory protein beta 1	2.6061
TESC	tescalcin	2.60404
KRT18	keratin 18	2.6023
SSH2	slingshot homolog 2 (Drosophila)	2.59526
PILRA	paired immunoglobin-like type 2 receptor alpha	2.5943
RAB7L1	RAB7, member RAS oncogene family-like 1	2.59327
CD180	CD180 molecule	2.59032
C20orf103	chromosome 20 open reading frame 103	2.58902
CD44	CD44 molecule (Indian blood group)	2.58849
MYBL1	v-myb myeloblastosis viral oncogene homolog (avian)-like 1	2.5869
DNAJA4	DnaJ (Hsp40) homolog, subfamily A, member 4	2.58658
LST1	leukocyte specific transcript 1	2.58282
TPPP	tubulin polymerization promoting protein	2.5815
CYYR1	cysteine/tyrosine-rich 1	2.58061
KLF1	Kruppel-like factor 1 (erythroid)	2.57981
SPTLC2	serine palmitoyltransferase, long chain base subunit 2	2.57412
GLIPR2	GLI pathogenesis-related 2	2.56588
SP140	SP140 nuclear body protein	2.56473
CXCL16	chemokine (C-X-C motif) ligand 16	2.56463
IGJ	ınoglobulin J polypeptide, linker protein for immunoglobulin alpha and m	2.56352
NFIX	nuclear factor I/X (CCAAT-binding transcription factor)	2.56069
GGA2	golgi-associated, gamma adaptin ear containing, ARF binding protein 2	2.55978
XPO7	exportin 7	2.55475
AFF3	AF4/FMR2 family, member 3	2.55208
OR52K3P	olfactory receptor, family 52, subfamily K, member 3 pseudogene	2.55136
TRBC1	T cell receptor beta constant 1	2.5452
TRIM58	tripartite motif-containing 58	2.54048
MGLL	monoglyceride lipase	2.53645
C9orf72	chromosome 9 open reading frame 72	2.53465
CIDEB	cell death-inducing DFFA-like effector b	2.53385
LOC144571	hypothetical protein LOC144571	2.53003
VIM	vimentin	2.52964
RHCE	Rh blood group, CcEe antigens	2.52911
C17orf103	chromosome 17 open reading frame 103	2.52086
ZFHX3	zinc finger homeobox 3	2.52085
LOC200772	hypothetical protein LOC200772	2.51994
MDM2	Mdm2 p53 binding protein homolog (mouse)	2.51695
ITK	IL2-inducible T-cell kinase	2.51298
TNFRSF21	tumor necrosis factor receptor superfamily, member 21	2.51226
IRS2	insulin receptor substrate 2	2.51012
OGFRL1	opioid growth factor receptor-like 1	2.50604
ANKRD10	Ankyrin repeat domain 10	2.50456

PPIL4	peptidylprolyl isomerase (cyclophilin)-like 4	2.50444
CLCN5	chloride channel 5	2.50238
PSMG4	Proteasome (prosome, macropain) assembly chaperone 4	2.50223
SLC25A39	solute carrier family 25, member 39	2.49969
MS4A14	membrane-spanning 4-domains, subfamily A, member 14	2.49737
RNF213	ring finger protein 213	2.49578
BTBD19	BTB (POZ) domain containing 19	2.49568
C5orf4	chromosome 5 open reading frame 4	2.49567
EVI2A	ecotropic viral integration site 2A	2.49448
CLEC5A	C-type lectin domain family 5, member A	2.49131
MAFF	v-maf musculoaponeurotic fibrosarcoma oncogene homolog F (avian)	2.49043
NLRC5	NLR family, CARD domain containing 5	2.48183
LOC100127972	hypothetical LOC100127972	2.4813
RIN2	Ras and Rab interactor 2	2.4796
PLEKHA1	pleckstrin homology domain containing, family A	2.4769
GJA3	gap junction protein, alpha 3, 46kDa	2.47622
TLR7	toll-like receptor 7	2.47549
LOC401233	similar to HIV TAT specific factor 1	2.47542
GZMK	granzyme K (granzyme 3; tryptase II)	2.47485
TMEM56	transmembrane protein 56	2.4734
KAT2B	K(lysine) acetyltransferase 2B	2.47185
CES1	carboxylesterase 1 (monocyte/macrophage serine esterase 1)	2.46896
S1PR3	sphingosine-1-phosphate receptor 3	2.45981
CG012	hypothetical gene CG012	2.45749
LOC150166	hypothetical protein LOC150166	2.45741
CIITA	class II, major histocompatibility complex, transactivator	2.45509
FAM107B	family with sequence similarity 107, member B	2.45492
SYCP2L	synaptonemal complex protein 2-like	2.45367
SOCS3	suppressor of cytokine signaling 3	2.4502
APP	amyloid beta (A4) precursor protein	2.44189
GOLGA8B	golgin A8 family, member B	2.44043
P2RY14	purinergic receptor P2Y, G-protein coupled, 14	2.43963
FCRL3	Fc receptor-like 3	2.43762
IL32	interleukin 32	2.436
TFEB	transcription factor EB	2.43598
EIF4A2	eukaryotic translation initiation factor 4A2	2.43415
CLIP4	CAP-GLY domain containing linker protein family, member 4	2.43187
CYorf15A	chromosome Y open reading frame 15A	2.4309
HERPUD1	nocysteine-inducible, endoplasmic reticulum stress-inducible, ubiquitin-lil	2.4292
FAR1	fatty acyl CoA reductase 1	2.42767
PYHIN1	pyrin and HIN domain family, member 1	2.42044
TNFAIP2	tumor necrosis factor, alpha-induced protein 2	2.42011
MPPED2	metallophosphoesterase domain containing 2	2.41531
PER1	period homolog 1 (Drosophila)	2.41501
SAMHD1	SAM domain and HD domain 1	2.40893
DMXL2	Dmx-like 2	2.40591

PIK3R5	phosphoinositide-3-kinase, regulatory subunit 5	2.40549
CD300A	CD300a molecule	2.39397
PTGS1	prostaglandin-endoperoxide synthase 1	2.39196
IFI44L	interferon-induced protein 44-like	2.39159
GZMH	granzyme H (cathepsin G-like 2, protein h-CCPX)	2.39053
STK17B	serine/threonine kinase 17b	2.38928
RBM6	RNA binding motif protein 6	2.38349
CPM	carboxypeptidase M	2.37749
RIOK3	RIO kinase 3 (yeast)	2.3774
POSTN	periostin, osteoblast specific factor	2.37561
C2	complement component 2	2.37183
NFKBIZ	lear factor of kappa light polypeptide gene enhancer in B-cells inhibitor, z	2.37112
MEF2C	myocyte enhancer factor 2C	2.37026
HLX	H2.0-like homeobox	2.36878
AKT3	V-akt murine thymoma viral oncogene homolog 3	2.36806
MYL4	myosin, light chain 4, alkali; atrial, embryonic	2.36566
IGF1	insulin-like growth factor 1 (somatomedin C)	2.36092
BAG3	BCL2-associated athanogene 3	2.3602
RELL1	RELT-like 1	2.35978
WDFY3	WD repeat and FYVE domain containing 3	2.35823
TSC22D3	TSC22 domain family, member 3	2.35639
RBPMS	RNA binding protein with multiple splicing	2.35634
WIPI1	WD repeat domain, phosphoinositide interacting 1	2.34891
SPATA13	Spermatogenesis associated 13	2.34886
G0S2	G0/G1switch 2	2.34832
DOK2	docking protein 2, 56kDa	2.34567
LRRC8C	leucine rich repeat containing 8 family, member C	2.34541
GIMAP6	GTPase, IMAP family member 6	2.34468
CAPN3	calpain 3, (p94)	2.34419
SEL1L3	sel-1 suppressor of lin-12-like 3 (C. elegans)	2.34326
WNK1	WNK lysine deficient protein kinase 1	2.34041
ID2	inhibitor of DNA binding 2, dominant negative helix-loop-helix protein	2.3387
EIF2C2	eukaryotic translation initiation factor 2C, 2	2.33668
SAT1	spermidine/spermine N1-acetyltransferase 1	2.3364
RHD	Rh blood group, D antigen	2.33464
EPB41	erythrocyte membrane protein band 4.1 (elliptocytosis 1, RH-linked)	2.33323
SOAT1	sterol O-acyltransferase 1	2.33248
XK	X-linked Kx blood group (McLeod syndrome)	2.33143
UNC13B	unc-13 homolog B (C. elegans)	2.33085
OSBP2	oxysterol binding protein 2	2.33073
VWA5A	von Willebrand factor A domain containing 5A	2.32787
SFRS3	Splicing factor, arginine/serine-rich 3	2.32527
FAM49A	family with sequence similarity 49, member A	2.32519
C10orf54	chromosome 10 open reading frame 54	2.32515
EGR3	early growth response 3	2.32398
KLF3	Kruppel-like factor 3 (basic)	2.32393

KMO	kynurenine 3-monooxygenase (kynurenine 3-hydroxylase)	2.32216
VAV2	vav 2 guanine nucleotide exchange factor	2.32003
TUBA4A	tubulin, alpha 4a	2.31929
FZD6	frizzled homolog 6 (Drosophila)	2.31591
ETNK1	Ethanolamine kinase 1	2.31475
LOC150381	hypothetical LOC150381	2.314
CHRDL1	chordin-like 1	2.3131
SLA	Src-like-adaptor	2.31277
MAST3	microtubule associated serine/threonine kinase 3	2.31151
NLRP12	NLR family, pyrin domain containing 12	2.30938
MAP2K3	mitogen-activated protein kinase kinase 3	2.30907
ANXA2	annexin A2	2.30836
MT1F	metallothionein 1F	2.30539
CLEC2D	C-type lectin domain family 2, member D	2.30251
SLC2A6	solute carrier family 2 (facilitated glucose transporter), member 6	2.29789
ACSL1	acyl-CoA synthetase long-chain family member 1	2.2974
HCK	hemopoietic cell kinase	2.29196
GDF15	growth differentiation factor 15	2.29105
FHDC1	FH2 domain containing 1	2.28693
CREB5	CAMP responsive element binding protein 5	2.2859
UTS2	urotensin 2	2.28579
RBM38	RNA binding motif protein 38	2.28578
SOD2	superoxide dismutase 2, mitochondrial	2.28211
MTSS1	metastasis suppressor 1	2.28006
TRIB1	tribbles homolog 1 (Drosophila)	2.27821
RGS2	regulator of G-protein signaling 2, 24kDa	2.27459
ATAD2B	ATPase family, AAA domain containing 2B	2.27212
ENC1	ectodermal-neural cortex 1 (with BTB-like domain)	2.26998
GRN	granulin	2.26602
BEX2	brain expressed X-linked 2	2.26576
CST3	cystatin C	2.26341
TMEM170B	transmembrane protein 170B	2.261
SESN3	sestrin 3	2.26084
H2AFY	H2A histone family, member Y	2.25676
RAPGEF2	Rap guanine nucleotide exchange factor (GEF) 2	2.25279
CCDC149	coiled-coil domain containing 149	2.2519
STOX2	storkhead box 2	2.24592
OLFML2A	olfactomedin-like 2A	2.24586
HEXIM1	hexamethylene bis-acetamide inducible 1	2.24346
PDE4A	phosphodiesterase 4A, cAMP-specific	2.24252
LOC400931	hypothetical LOC400931	2.24233
MMRN1	multimerin 1	2.24179
PCNX	pecanex homolog (Drosophila)	2.2408
AHR	aryl hydrocarbon receptor	2.23981
LY9	lymphocyte antigen 9	2.23894
QPCT	glutaminyl-peptide cyclotransferase	2.23338

TOB1	transducer of ERBB2, 1	2.23141
RNFT2	ring finger protein, transmembrane 2	2.22505
KIAA1598	KIAA1598	2.22407
SRD5A1	steroid-5-alpha-reductase, alpha polypeptide 1	2.2239
IL13RA1	interleukin 13 receptor, alpha 1	2.22272
PPAP2B	phosphatidic acid phosphatase type 2B	2.22265
FRMD4A	FERM domain containing 4A	2.22223
RNASE6	ribonuclease, RNase A family, k6	2.22098
C12orf59	chromosome 12 open reading frame 59	2.21966
HIPK3	homeodomain interacting protein kinase 3	2.21656
CSGALNACT1	chondroitin sulfate N-acetylgalactosaminyltransferase 1	2.21645
LYN	v-yes-1 Yamaguchi sarcoma viral related oncogene homolog	2.21457
OAS2	2'-5'-oligoadenylate synthetase 2, 69/71kDa	2.21343
FCGR1B	Fc fragment of IgG, high affinity Ib, receptor (CD64)	2.21005
DENND4A	DENN/MADD domain containing 4A	2.20899
UBE2D1	ubiquitin-conjugating enzyme E2D 1 (UBC4/5 homolog, yeast)	2.20892
HLA-F	major histocompatibility complex, class I, F	2.19941
LILRA5	ocyte immunoglobulin-like receptor, subfamily A (with TM domain), mem	2.19823
ATP7A	ATPase, Cu++ transporting, alpha polypeptide	2.19624
FCGRT	Fc fragment of IgG, receptor, transporter, alpha	2.19497
EDEM1	ER degradation enhancer, mannosidase alpha-like 1	2.19082
ASAP1	ArfGAP with SH3 domain, ankyrin repeat and PH domain 1	2.18723
HEPACAM2	HEPACAM family member 2	2.18707
ISG20	interferon stimulated exonuclease gene 20kDa	2.18279
GNAI1	anine nucleotide binding protein (G protein), alpha inhibiting activity poly	2.18169
LCK	lymphocyte-specific protein tyrosine kinase	2.18038
STAB1	stabilin 1	2.17852
LOC100190986	hypothetical LOC100190986	2.17782
NR4A1	nuclear receptor subfamily 4, group A, member 1	2.17741
CD300LF	CD300 molecule-like family member f	2.17423
OPTN	optineurin	2.1729
FERMT1	fermitin family homolog 1 (Drosophila)	2.16726
WDR26	WD repeat domain 26	2.16448
CD4	CD4 molecule	2.16167
MARCH8	membrane-associated ring finger (C3HC4) 8	2.1615
CD3D	CD3d molecule, delta (CD3-TCR complex)	2.16124
CALML4	calmodulin-like 4	2.1578
BPGM	2,3-bisphosphoglycerate mutase	2.15718
CD27	CD27 molecule	2.15652
ABCB6	ATP-binding cassette, sub-family B (MDR/TAP), member 6	2.15482
TFEC	transcription factor EC	2.15474
HVCN1	hydrogen voltage-gated channel 1	2.15426
GALNT10	N-acetyl-alpha-D-galactosamine:polypeptide N-acetylgalactosaminyltrans	2.15257
BHLHE40	basic helix-loop-helix family, member e40	2.15251
FCGR1A	Fc fragment of IgG, high affinity Ia, receptor (CD64)	2.15201
CAMK1D	calcium/calmodulin-dependent protein kinase ID	2.1514

NFKBID	lear factor of kappa light polypeptide gene enhancer in B-cells inhibitor, d	2.15132
ENTPD1	ectonucleoside triphosphate diphosphohydrolase 1	2.15131
KLF2	Kruppel-like factor 2 (lung)	2.15082
PRDM1	PR domain containing 1, with ZNF domain	2.15042
APOB48R	apolipoprotein B48 receptor	2.14776
PHLDA2	pleckstrin homology-like domain, family A, member 2	2.1473
HPSE	heparanase	2.1454
TICAM2	toll-like receptor adaptor molecule 2	2.14507
ANXA2P2	annexin A2 pseudogene 2	2.14481
CCDC50	coiled-coil domain containing 50	2.1422
NAA15	N(alpha)-acetyltransferase 15, NatA auxiliary subunit	2.1413
ZNF844	zinc finger protein 844	2.13861
DIRC2	disrupted in renal carcinoma 2	2.13748
POU2F2	POU class 2 homeobox 2	2.13627
CELF1	CUGBP, Elav-like family member 1	2.13528
HK1	hexokinase 1	2.13059
CLU	clusterin	2.13045
AGTRAP	angiotensin II receptor-associated protein	2.13029
RAB20	RAB20, member RAS oncogene family	2.12983
LILRA5	ocyte immunoglobulin-like receptor, subfamily A (with TM domain), mem	2.12564
ANXA1	Annexin A1	2.1253
C5orf32	chromosome 5 open reading frame 32	2.1214
IL17RA	interleukin 17 receptor A	2.11892
ZCCHC6	zinc finger, CCHC domain containing 6	2.11851
FAM13A	family with sequence similarity 13, member A	2.11841
NBPF10	neuroblastoma breakpoint family, member 10	2.11801
FOS	FBJ murine osteosarcoma viral oncogene homolog	2.11707
CCL4	chemokine (C-C motif) ligand 4	2.11505
HTATIP2	HIV-1 Tat interactive protein 2, 30kDa	2.11163
P2RY13	purinergic receptor P2Y, G-protein coupled, 13	2.10914
FAM124A	family with sequence similarity 124A	2.10589
PPTC7	PTC7 protein phosphatase homolog (S. cerevisiae)	2.10488
IGSF10	immunoglobulin superfamily, member 10	2.10282
CAMK2D	calcium/calmodulin-dependent protein kinase II delta	2.10187
PTRF	polymerase I and transcript release factor	2.10023
MSR1	macrophage scavenger receptor 1	2.09993
TCF7L2	Transcription factor 7-like 2 (T-cell specific, HMG-box)	2.09957
HLA-E	major histocompatibility complex, class I, E	2.09847
ABLIM1	actin binding LIM protein 1	2.09731
TNNT1	troponin T type 1 (skeletal, slow)	2.09638
ENPP5	ectonucleotide pyrophosphatase/phosphodiesterase 5 (putative function	2.09588
CD8A	CD8a molecule	2.09537
LOC100131993	similar to hCG2020760	2.09532
SH2B3	SH2B adaptor protein 3	2.09497
EPB49	erythrocyte membrane protein band 4.9 (dematin)	2.09409
REEP5	receptor accessory protein 5	2.09316

TNFSF12	tumor necrosis factor (ligand) superfamily, member 12	2.09305
MCTP1	multiple C2 domains, transmembrane 1	2.09201
GGT1	gamma-glutamyltransferase 1	2.09189
NPTX2	neuronal pentraxin II	2.09088
LACTB	lactamase, beta	2.08776
CD68	CD68 molecule	2.08591
FCGR2B	Fc fragment of IgG, low affinity IIb, receptor (CD32)	2.08288
TSPO2	translocator protein 2	2.08103
ZNF467	zinc finger protein 467	2.08065
FRAT1	frequently rearranged in advanced T-cell lymphomas	2.07997
GATA3	GATA binding protein 3	2.07785
TLR1	toll-like receptor 1	2.07756
CMTM4	CKLF-like MARVEL transmembrane domain containing 4	2.07722
VSIG4	V-set and immunoglobulin domain containing 4	2.07675
CREM	cAMP responsive element modulator	2.07585
CDC42BPB	CDC42 binding protein kinase beta (DMPK-like)	2.07534
CXCR2	chemokine (C-X-C motif) receptor 2	2.07431
FOXO3	forkhead box O3	2.07374
TXNIP	thioredoxin interacting protein	2.07016
USP31	ubiquitin specific peptidase 31	2.0697
CDH2	cadherin 2, type 1, N-cadherin (neuronal)	2.06923
E2F2	E2F transcription factor 2	2.06855
FCGR2C	:c fragment of IgG, low affinity IIc, receptor for (CD32) (gene/pseudogene	2.06742
PPP1R16B	protein phosphatase 1, regulatory (inhibitor) subunit 16B	2.06518
IGKC	immunoglobulin kappa constant	2.06463
C1orf56	chromosome 1 open reading frame 56	2.06339
CYP1B1	cytochrome P450, family 1, subfamily B, polypeptide 1	2.06216
LAPTM5	lysosomal protein transmembrane 5	2.05803
ZAK	sterile alpha motif and leucine zipper containing kinase AZK	2.05589
PLCL2	phospholipase C-like 2	2.05549
SLCO4C1	solute carrier organic anion transporter family, member 4C1	2.05366
TPPP3	tubulin polymerization-promoting protein family member 3	2.05312
LRIG1	leucine-rich repeats and immunoglobulin-like domains 1	2.05297
OAZ3	ornithine decarboxylase antizyme 3	2.05294
ARTN	artemin	2.04938
RASGRP4	RAS guanyl releasing protein 4	2.04574
EZR	ezrin	2.04547
GNPDA1	glucosamine-6-phosphate deaminase 1	2.04269
GVIN1	GTPase, very large interferon inducible 1	2.04182
STS	steroid sulfatase (microsomal), isozyme S	2.04174
PAM	peptidylglycine alpha-amidating monooxygenase	2.04069
PRDX2	peroxiredoxin 2	2.03882
FOSB	FBJ murine osteosarcoma viral oncogene homolog B	2.03559
PTPRN2	protein tyrosine phosphatase, receptor type, N polypeptide 2	2.03346
ARF6	ADP-ribosylation factor 6	2.03317
HBG1	hemoglobin, gamma A	2.03182

FAIM3	Fas apoptotic inhibitory molecule 3	2.03161
CDH1	cadherin 1, type 1, E-cadherin (epithelial)	2.03127
NFAM1	NFAT activating protein with ITAM motif 1	2.0311
ADPRH	ADP-ribosylarginine hydrolase	2.03039
SERPINB2	serpin peptidase inhibitor, clade B (ovalbumin), member 2	2.02939
FAM83A	family with sequence similarity 83, member A	2.02853
GPR124	G protein-coupled receptor 124	2.02691
C1QC	complement component 1, q subcomponent, C chain	2.0259
ATP6V1A	ATPase, H+ transporting, lysosomal 70kDa, V1 subunit A	2.02506
C6orf62	Chromosome 6 open reading frame 62	2.02505
CTSZ	cathepsin Z	2.02494
SEC14L1	SEC14-like 1 (S. cerevisiae)	2.02325
LARP7	La ribonucleoprotein domain family, member 7	2.02221
TRAC	T cell receptor alpha constant	2.02211
SP1	Sp1 transcription factor	2.01913
MAML2	mastermind-like 2 (Drosophila)	2.01896
SEP9	septin 9	2.01881
LSP1	lymphocyte-specific protein 1	2.01855
HEATR5A	HEAT repeat containing 5A	2.01784
KLHL2	kelch-like 2, Mayven (Drosophila)	2.01337
GLI2	GLI family zinc finger 2	2.01189
MMP15	matrix metallopeptidase 15 (membrane-inserted)	2.01101
HLA-DMA	major histocompatibility complex, class II, DM alpha	2.01001
ARRB2	arrestin, beta 2	2.00904
MEIS1	Meis homeobox 1	2.00851
CD200	CD200 molecule	2.00746
CCR6	chemokine (C-C motif) receptor 6	2.00524
STX16	syntaxin 16	2.0038
TADA2B	transcriptional adaptor 2B	2.00217
CTTN	cortactin	2.00213
