# Supplementary material for: DNMT3A mutants provide proliferating advantage with augmentation of self-renewal activity in the pathogenesis of AML in KMT2A-PTD-positive leukemic cells
Source: Oncogenesis. 2020 Feb 3;9(2):7. doi: 10.1038/s41389-020-0191-6 (PMC6997180; doi:10.1038/s41389-020-0191-6)
Supplement: Supplementary file 15 — Dataset S2 [file 41389_2020_191_MOESM15_ESM.pdf]

List of downregulated (<-2 folds) genes in KMT2A-PTD/DNMT3A-MT AML cells compared to KMT2A-PTD/DNMT3A-WT cells

Gene Symbol	Gene Title	Fold-Change
C1QTNF4	C1q and tumor necrosis factor related protein 4	-7.82266
PRSS21	protease, serine, 21 (testisin)	-7.59796
IKZF2	IKAROS family zinc finger 2 (Helios)	-5.33019
ZNF521	zinc finger protein 521	-5.07743
IRX3	iroquois homeobox 3	-4.98089
TWIST1	twist homolog 1 (Drosophila)	-4.76687
C1orf186	chromosome 1 open reading frame 186	-4.57139
MLLT11	loid/lymphoid or mixed-lineage leukemia (trithorax homolog, Drosophila);	-4.48535
SLITRK4	SLIT and NTRK-like family, member 4	-4.32035
RHOBTB1	Rho-related BTB domain containing 1	-4.09441
STAR	steroidogenic acute regulatory protein	-4.04968
C19orf77	chromosome 19 open reading frame 77	-4.03542
LIMA1	LIM domain and actin binding 1	-4.02208
MEST	mesoderm specific transcript homolog (mouse)	-3.55054
SLITRK5	SLIT and NTRK-like family, member 5	-3.53285
LOC285628	hypothetical protein LOC285628	-3.49773
C5orf39	chromosome 5 open reading frame 39	-3.49326
HOPX	HOP homeobox	-3.48988
CXorf38	chromosome X open reading frame 38	-3.45785
LOC339862	hypothetical protein LOC339862	-3.38606
CCNA1	cyclin A1	-3.29882
TSIX	XIST antisense RNA (non-protein coding)	-3.26539
CPA3	carboxypeptidase A3 (mast cell)	-3.24226
BAMBI	BMP and activin membrane-bound inhibitor homolog (Xenopus laevis)	-3.21091
JPH1	junctophilin 1	-3.16284
C9orf43	chromosome 9 open reading frame 43	-3.14527
TMEM107	transmembrane protein 107	-3.14266
ADAMDEC1	ADAM-like, decysin 1	-3.07796
KIAA1211	KIAA1211	-3.04937
LOC100288152	Hypothetical protein LOC100288152	-3.04845
CFI	complement factor I	-3.04405
APOBEC3B	apolipoprotein B mRNA editing enzyme, catalytic polypeptide-like 3B	-3.02662
C7orf58	chromosome 7 open reading frame 58	-3.0236
ADAMTS1	ADAM metallopeptidase with thrombospondin type 1 motif, 1	-3.02143
JUN	jun oncogene	-3.01323
DPP10	dipeptidyl-peptidase 10 (non-functional)	-2.97925
HPGDS	hematopoietic prostaglandin D synthase	-2.96228
MARCKSL1	MARCKS-like 1	-2.94649
DEPDC7	DEP domain containing 7	-2.93917
MEX3C	mex-3 homolog C (C. elegans)	-2.91694
ZNF667	zinc finger protein 667	-2.91475
THSD7A	thrombospondin, type I, domain containing 7A	-2.88896

AEBP1	AE binding protein 1	-2.84835
ATP9A	ATPase, class II, type 9A	-2.82144
SFTA3	surfactant associated 3	-2.80288
TRAT1	T cell receptor associated transmembrane adaptor 1	-2.7963
IGLL1	immunoglobulin lambda-like polypeptide 1	-2.79368
MAPK12	mitogen-activated protein kinase 12	-2.791
TMEM107	transmembrane protein 107	-2.75342
C11orf95	chromosome 11 open reading frame 95	-2.75193
FAM69B	family with sequence similarity 69, member B	-2.73827
ABP1	amiloride binding protein 1 (amine oxidase (copper-containing))	-2.73817
NAP1L2	nucleosome assembly protein 1-like 2	-2.72674
CBX2	chromobox homolog 2 (Pc class homolog, Drosophila)	-2.68942
MLL	lyeloid/lymphoid or mixed-lineage leukemia (trithorax homolog, Drosophil	-2.65706
COL24A1	collagen, type XXIV, alpha 1	-2.65314
EMX2	empty spiracles homeobox 2	-2.65235
ATP8B3	ATPase, aminophospholipid transporter, class I, type 8B, member 3	-2.63791
SIKE1	suppressor of IKBKE 1	-2.62945
ITM2A	integral membrane protein 2A	-2.60574
MGC9913	hypothetical protein MGC9913	-2.60528
CRISPLD1	cysteine-rich secretory protein LCCL domain containing 1	-2.59847
BEND4	BEN domain containing 4	-2.59305
LOC285965	hypothetical protein LOC285965	-2.58935
ANKRD18A	ankyrin repeat domain 18A	-2.58638
ST7	suppression of tumorigenicity 7	-2.58078
SLC27A2	solute carrier family 27 (fatty acid transporter), member 2	-2.56607
TRIM73	tripartite motif-containing 73	-2.539
MANEAL	mannosidase, endo-alpha-like	-2.53077
CRNDE	colorectal neoplasia differentially expressed (non-protein coding)	-2.52166
AK5	adenylate kinase 5	-2.52023
KIAA1958	KIAA1958	-2.51351
ADA	adenosine deaminase	-2.50946
BEND6	BEN domain containing 6	-2.50578
CHST12	carbohydrate (chondroitin 4) sulfotransferase 12	-2.5043
FLJ32255	hypothetical protein LOC643977	-2.49282
LRRC34	leucine rich repeat containing 34	-2.47963
BCAT1	branched chain amino-acid transaminase 1, cytosolic	-2.47434
NOG	noggin	-2.45531
LOC100293492	hypothetical protein LOC100293492	-2.45522
NUMA1	nuclear mitotic apparatus protein 1	-2.4514
ERBB4	v-erb-a erythroblastic leukemia viral oncogene homolog 4 (avian)	-2.44605
PAWR	PRKC, apoptosis, WT1, regulator	-2.44484
ACSM3	acyl-CoA synthetase medium-chain family member 3	-2.43964
SEPT6	septin 6	-2.42977
STAP1	signal transducing adaptor family member 1	-2.42159
ZNRF1	zinc and ring finger 1	-2.39165
PUS7	pseudouridylate synthase 7 homolog (S. cerevisiae)	-2.38472

BCL10	B-cell CLL/lymphoma 10	-2.37925
PLD1	phospholipase D1, phosphatidylcholine-specific	-2.36229
IGFBP2	insulin-like growth factor binding protein 2, 36kDa	-2.35338
LTK	leukocyte receptor tyrosine kinase	-2.34861
C5orf13	chromosome 5 open reading frame 13	-2.3429
SERF1A	small EDRK-rich factor 1A (telomeric)	-2.32248
PDZD8	PDZ domain containing 8	-2.31072
NEFH	neurofilament, heavy polypeptide	-2.3106
DPY19L2	dpy-19-like 2 (C. elegans)	-2.31054
CYTH2	cytohesin 2	-2.30966
FOXC1	forkhead box C1	-2.30408
HOXA9	homeobox A9	-2.30025
SNCAIP	synuclein, alpha interacting protein	-2.29993
CHKB-CPT1B	ıoline kinase-like, carnitine palmitoyltransferase 1B (muscle) transcription ı	-2.29897
TMSB15B	thymosin beta 15B	-2.28765
C1orf150	chromosome 1 open reading frame 150	-2.28474
CCDC58	coiled-coil domain containing 58	-2.28332
ANXA8	annexin A8	-2.27826
CP	ceruloplasmin (ferroxidase)	-2.27681
TOX	thymocyte selection-associated high mobility group box	-2.27228
SFRS3	Splicing factor, arginine/serine-rich 3	-2.26496
LOC387647	patched domain containing 3 pseudogene	-2.25904
AGR2	anterior gradient homolog 2 (Xenopus laevis)	-2.25402
CAV2	caveolin 2	-2.25204
NRXN2	neurexin 2	-2.24898
TSGA14	testis specific, 14	-2.24833
FLJ39051	Hypothetical protein LOC399972	-2.24665
LIN7A	lin-7 homolog A (C. elegans)	-2.23972
LPAR4	lysophosphatidic acid receptor 4	-2.22806
SVOPL	SVOP-like	-2.22549
ZNF678	zinc finger protein 678	-2.22443
TRH	thyrotropin-releasing hormone	-2.2187
NEGR1	neuronal growth regulator 1	-2.21869
H2BFXP	H2B histone family, member X, pseudogene	-2.21451
SNHG3	small nucleolar RNA host gene 3 (non-protein coding)	-2.21322
IRX5	iroquois homeobox 5	-2.19437
PPP1R9A	protein phosphatase 1, regulatory (inhibitor) subunit 9A	-2.187
SLC39A10	solute carrier family 39 (zinc transporter), member 10	-2.18587
PPAT	phosphoribosyl pyrophosphate amidotransferase	-2.18494
NUCB2	nucleobindin 2	-2.18388
NDFIP2	Nedd4 family interacting protein 2	-2.18355
TBXA2R	thromboxane A2 receptor	-2.18211
KCNQ5	potassium voltage-gated channel, KQT-like subfamily, member 5	-2.18117
CTPS2	CTP synthase II	-2.17597
C5orf35	chromosome 5 open reading frame 35	-2.17544
ATL1	atlastin GTPase 1	-2.17376

CEL	carboxyl ester lipase (bile salt-stimulated lipase)	-2.16972
COL4A5	collagen, type IV, alpha 5	-2.16497
NASP	Nuclear autoantigenic sperm protein (histone-binding)	-2.15943
C5orf54	chromosome 5 open reading frame 54	-2.15928
C12orf73	chromosome 12 open reading frame 73	-2.15815
KCNK5	potassium channel, subfamily K, member 5	-2.15664
C3orf34	chromosome 3 open reading frame 34	-2.15639
DNAJC12	DnaJ (Hsp40) homolog, subfamily C, member 12	-2.15624
FGFR1	fibroblast growth factor receptor 1	-2.1537
C9orf122	chromosome 9 open reading frame 122	-2.14893
ARNTL2	aryl hydrocarbon receptor nuclear translocator-like 2	-2.14131
TSPAN13	tetraspanin 13	-2.12908
SLC35F2	solute carrier family 35, member F2	-2.12852
RNASEH2B	ribonuclease H2, subunit B	-2.12705
PAPOLA	poly(A) polymerase alpha	-2.12253
MIB1	mindbomb homolog 1 (Drosophila)	-2.12104
ZNF117	zinc finger protein 117	-2.1156
FKBP7	FK506 binding protein 7	-2.11362
BNIP3	BCL2/adenovirus E1B 19kDa interacting protein 3	-2.11072
MSRB3	methionine sulfoxide reductase B3	-2.10136
ZNF677	zinc finger protein 677	-2.09449
CCDC125	coiled-coil domain containing 125	-2.09294
LOC286467	hypothetical LOC286467	-2.08743
B4GALT6	UDP-Gal:betaGlcNAc beta 1,4- galactosyltransferase, polypeptide 6	-2.08473
DPY19L2P2	dpy-19-like 2 pseudogene 2 (C. elegans)	-2.08408
NLRP2	NLR family, pyrin domain containing 2	-2.082
SSBP2	single-stranded DNA binding protein 2	-2.07476
ARMC8	armadillo repeat containing 8	-2.07214
KHDRBS3	KH domain containing, RNA binding, signal transduction associated 3	-2.07211
DNMT3A	DNA (cytosine-5-)-methyltransferase 3 alpha	-2.06924
B3GALTL	beta 1,3-galactosyltransferase-like	-2.06834
CERCAM	cerebral endothelial cell adhesion molecule	-2.06625
EMX2OS	EMX2 opposite strand (non-protein coding)	-2.06605
NTNG2	netrin G2	-2.06539
CASP6	caspase 6, apoptosis-related cysteine peptidase	-2.06154
C1orf228	chromosome 1 open reading frame 228	-2.0609
CREB3L4	cAMP responsive element binding protein 3-like 4	-2.05899
FGFR1	fibroblast growth factor receptor 1	-2.05768
STRAP	Serine/threonine kinase receptor associated protein	-2.05678
PTPRG	protein tyrosine phosphatase, receptor type, G	-2.05532
DHTKD1	dehydrogenase E1 and transketolase domain containing 1	-2.05481
FAM30A	family with sequence similarity 30, member A	-2.05367
RAB33A	RAB33A, member RAS oncogene family	-2.05337
CENPV	centromere protein V	-2.05326
PPP1R3E	protein phosphatase 1, regulatory (inhibitor) subunit 3E	-2.05306
MAP1A	microtubule-associated protein 1A	-2.05276

MATK	megakaryocyte-associated tyrosine kinase	-2.0499
ANKRD27	ankyrin repeat domain 27 (VPS9 domain)	-2.04937
XIST	X (inactive)-specific transcript (non-protein coding)	-2.04784
ACTR2	ARP2 actin-related protein 2 homolog (yeast)	-2.04672
CDK6	cyclin-dependent kinase 6	-2.04426
C7orf68	chromosome 7 open reading frame 68	-2.03999
SOX4	SRY (sex determining region Y)-box 4	-2.03949
PHF16	PHD finger protein 16	-2.03816
NHEDC2	Na+/H+ exchanger domain containing 2	-2.03742
CCDC34	coiled-coil domain containing 34	-2.03361
AVEN	apoptosis, caspase activation inhibitor	-2.03195
TFPI	issue factor pathway inhibitor (lipoprotein-associated coagulation inhibitor	-2.02854
LOC253039	hypothetical LOC253039	-2.02793
SORD	sorbitol dehydrogenase	-2.02756
HLA-DRB1	major histocompatibility complex, class II, DR beta 1	-2.02397
TRNT1	tRNA nucleotidyl transferase, CCA-adding, 1	-2.02334
HPGD	hydroxyprostaglandin dehydrogenase 15-(NAD)	-2.02114
SMAD5	SMAD family member 5	-2.01613
H3F3B	H3 histone, family 3B (H3.3B)	-2.01297
ZNF512B	zinc finger protein 512B	-2.01279
MLC1	megalencephalic leukoencephalopathy with subcortical cysts 1	-2.01179
STON1	stonin 1	-2.00918
ACAD11	acyl-CoA dehydrogenase family, member 11	-2.0084
CCNL1	cyclin L1	-2.00659
PM20D2	peptidase M20 domain containing 2	-2.00656
SSX2IP	synovial sarcoma, X breakpoint 2 interacting protein	-2.00588
TIPRL	TIP41, TOR signaling pathway regulator-like (S. cerevisiae)	-2.00391
MSI2	musashi homolog 2 (Drosophila)	-2.00087
LOC729570	hypothetical LOC729570	-2.00032
