# Supplementary material for: DNMT3A mutants provide proliferating advantage with augmentation of self-renewal activity in the pathogenesis of AML in KMT2A-PTD-positive leukemic cells
Source: Oncogenesis. 2020 Feb 3;9(2):7. doi: 10.1038/s41389-020-0191-6 (PMC6997180; doi:10.1038/s41389-020-0191-6)
Supplement: Supplementary file 16 — Dataset S3 [file 41389_2020_191_MOESM16_ESM.pdf]

List of differentially methylated (differential β-value <-0.3) genes in DNMT3A-R882C-expressing EOL-1 cells compared to DNMT3A-WT-expressing EOL-1 cells, which were upregulated (>2 folds) in KMT2A-PTD/DNMT3A-MT AML cells compared to KMT2A-PTD/DNMT3A-WT cells

Gene Symbol	β-value difference	UCSC_REFGENE_GROUP
C10orf54	-0.7448533	TSS1500; Body
CRISPLD2	-0.7340438	5'UTR
FRMD3	-0.7248747	TSS1500; Body
EPB41L3	-0.69948546	Body
FHL1	-0.6945275	Body; 5'UTR
RASSF4	-0.67277678	TSS200; Body
RAB20	-0.6707888	Body; 1stExon
BHLHE40	-0.6622778	Body
ZFHX3	-0.6488489	Body; 5'UTR
PILRA	-0.6464545	TSS1500
UBE2D1	-0.6423429	5'UTR; Body
EMP1	-0.6370132	TSS1500
TSC22D3	-0.62739079	5'UTR; Body; 1stExon
SH3TC1	-0.6230362	Body
KLF9	-0.6213391	Body
FAM198B	-0.616808	Body; TSS1500
BCL6	-0.6143837	TSS1500;5'UTR
FAM13A	-0.6130758	Body
IRF8	-0.6101864	Body, TSS1500
IQSEC1	-0.609829	Body; TSS1500
S100A8	-0.6053717	5'UTR; TSS200
GZMK	-0.6018102	Body; TSS1500
LRRK2	-0.6010765	Body
ACSL1	-0.5997377	TSS1500; 5'UTR
ANKH	-0.5970819	Body
CLCN5	-0.5965196	Body
PTPRN2	-0.5910232	Body
PTPRE	-0.589514	5'UTR; Body; TSS1500
PRR5L	-0.5865238	5'UTR; Body
HEMGN	-0.5861669	Body
SVIL	-0.5788705	5'UTR; TSS1500
FLVCR2	-0.5759042	1stExon
SLC8A1	-0.5739802	Body
C17orf91	-0.5733334	Body
PHLDA2	-0.5724622	5'UTR; 1stExon
FRMD4A	-0.5707413	Body
TPM1	-0.5682452	Body
MLLT4	-0.56638611	5'UTR; Body
PLCL2	-0.566385	Body
TBL1X	-0.5636624	5'UTR;TSS1500
ISG20	-0.5632623	5'UTR; Body
SH2B3	-0.56283	1stExon; Body; TSS200
SLA	-0.56273	TSS1500; Body; 5'UTR

DOCK10	-0.5624155	Body
ALOX5	-0.5609773	Body
COTL1	-0.5608404	Body
FAM107B	-0.5584019	Body; 5'UTR
PSTPIP1	-0.5577007	Body
SRGN	-0.5567712	TSS1500
MGLL	-0.5556679	Body
LDLRAD3	-0.54663804	5'UTR; Body
MFHAS1	-0.545402	Body
ANXA2	-0.5384838	5'UTR
SSH2	-0.5364392	Body; TSS1500
RAP1GAP	-0.5364303	Body; 5'UTR
PPP1R16B	-0.5310447	Body
CD36	-0.5285476	TSS1500; 5'UTR
KCTD12	-0.5279445	TSS1500
POU2F2	-0.5244348	Body; TSS1500
FNIP2	-0.5227966	Body
MAFF	-0.5223219	5'UTR
TLR5	-0.5218563	5'UTR
CD48	-0.5215979	TSS1500
UBAC2	-0.5199648	TSS1500;Body;Body;Body
CD109	-0.5182999	Body
RHCE	-0.5182939	Body
TADA2B	-0.5180837	Body
LSP1	-0.5176125	5'UTR;1stExon;Body; TSS200
DAAM1	-0.5159518	5'UTR. Body
CAMK2D	-0.5139878	Body
ALOX5AP	-0.5139547	Body
ARHGAP21	-0.5138569	Body
TOB1	-0.5136134	TSS1500
BACH2	-0.5132258	Body
GLI2	-0.5131778	Body
NID1	-0.5130828	Body
AKAP13	-0.5129723	5'UTR
PRKCA	-0.5096307	Body
ZAK	-0.5095449	TSS1500
TNFRSF21	-0.5095178	Body
GGT1	-0.5092209	TSS1500; 5'UTR
SERPINA1	-0.5076948	3'UTR; TSS1500
PDE4B	-0.5072849	Body; TSS200
WIPI1	-0.5063712	Body
SV2B	-0.5050368	Body; 5'UTR
TMEM204	-0.5025248	Body
RELL1	-0.5023235	Body
ZBTB38	-0.5016118	5'UTR
MAML2	-0.5009717	Body
AGPAT9	-0.5001985	TSS1500; TSS200
NBPF10	-0.4998325	TSS1500; Body

SOX6	-0.4997559	Body; 5'UTR; TSS1500
ZCCHC6	-0.4997508	Body
CCDC50	-0.4996355	1stExon; Body; TSS200; 5'UTR
GJA3	-0.4991543	5'UTR
AKT3	-0.4984337	Body
NLRP1	-0.4951142	5'UTR; 1stExon
PRKAR2B	-0.494937	Body
HLA-DPA1	-0.4949012	Body
DPP4	-0.4946085	TSS200
KAT2B	-0.4934696	Body
MEF2C	-0.4926797	Body
GZMA	-0.4907764	TSS1500
MARCH1	-0.4901049	Body
METRNL	-0.4898561	Body
DLG5	-0.4895952	Body
RGS2	-0.4895047	TSS1500
CSGALNACT1	-0.4892977	Body
CYorf15A	-0.4889303	TSS1500
TNS1	-0.4879496	Body
TBC1D8	-0.487796	Body
SPATA13	-0.4876146	TSS1500; Body
OSBP2	-0.4871527	Body
LMNA	-0.4852475	TSS1500; 5'UTR
UNC13B	-0.4829811	Body
LRRFIP1	-0.4824296	Body
ABLIM1	-0.4823841	Body; TSS1500
NLRC5	-0.4802267	Body
PIK3R5	-0.4798723	5'UTR
SYNJ2	-0.4793395	Body; TSS200
PTRF	-0.47907293	Body
RAPGEF2	-0.4779515	Body
DNTT	-0.4769257	Body
CD86	-0.4767302	5'UTR; Body; TSS1500; 1stExon
DNAJA4	-0.4765609	TSS200; Body
VAV2	-0.4762945	Body; Body
SULF2	-0.4759795	Body
LYN	-0.47553255	TSS1500
AFF3	-0.4749197	Body
MYH10	-0.4745814	Body
ZNF185	-0.4737663	Body
IL6ST	-0.4722394	Body; 5'UTR
RORA	-0.4721302	Body
PTPRC	-0.4691066	Body
PPTC7	-0.4683741	Body
CREB5	-0.4682421	5'UTR
CTSZ	-0.4640994	TSS1500
NLRP3	-0.4640821	TSS1500
RXRA	-0.46387501	Body

ASAP1	-0.4613817	Body; TSS1500
MS4A6A	-0.459494	TSS1500
KMO	-0.4582141	Body
CECR1	-0.4579015	5'UTR
PSAP	-0.4565745	TSS1500
TGFBI	-0.4556418	TSS1500
ANK1	-0.4525256	Body
HK3	-0.4514168	TSS200
ENPP5	-0.4503968	TSS1500
MEIS1	-0.4497944	3'UTR
DOK2	-0.449545	Body
HLA-DRB1	-0.4492423	3'UTR
PCNX	-0.44913	Body; TSS1500
SAMSN1	-0.4482174	Body
LGALS3	-0.447143	TSS200
CAMK1D	-0.4462876	Body
NOD2	-0.445834	TSS1500
BCL11B	-0.4458238	TSS200
LATS2	-0.445821	TSS1500
SGK1	-0.4453821	Body; TSS200; TSS1500
PADI4	-0.44529465	Body
ALDH1A1	-0.4452888	Body
MCL1	-0.443913	TSS1500
CD34	-0.441925	1stExon; 5'UTR
CD52	-0.4408143	TSS200; 5'UTR
TMCC2	-0.4378504	Body; TSS1500
FAM65C	-0.4348094	Body
BASP1	-0.4342952	Body; TSS1500
SIDT1	-0.4333144	Body
ENTPD1	-0.4319658	Body; 5'UTR
CXCR4	-0.4312863	3'UTR; 1stExon
CLEC2D	-0.4304484	TSS1500
MCTP1	-0.4302984	Body
CYP1B1	-0.4300579	Body
LOC150381	-0.4295006	Body; TSS1500
CELF1	-0.4289571	1stExon; 5'UTR
LRRC8C	-0.4282353	5'UTR; TSS1500; Body
FERMT1	-0.4280507	Body
MARCH8	-0.4276344	TSS200
SLC2A6	-0.4256721	Body
EPB41	-0.4244789	5'UTR
FAM124A	-0.4244743	Body
NACC2	-0.4242108	5'UTR
TLR4	-0.4238864	Body; 3'UTR
IGF2R	-0.42300699	Body
FRAT1	-0.4217034	TSS1500
STOX2	-0.4211109	Body
CMTM4	-0.4201415	Body

MX1	-0.4173493	Body
CPEB4	-0.4163958	Body
RASGEF1B	-0.4162298	TSS1500
CACNA2D3	-0.4157644	TSS1500; Body
GABBR1	-0.415323	Body, TSS1500
BAG3	-0.4152779	Body; TSS1500
PDZK1IP1	-0.41525787	Body
RHOU	-0.4122259	Body
EPS8	-0.4118753	TSS200
GZMH	-0.41127101	Body
MYOF	-0.4107823	Body
GATA3	-0.4106491	Body
GALNT10	-0.4103069	Body; 3'UTR
HLA-DRA	-0.4101755	TSS1500
TRIM10	-0.4100815	Body
C1orf38	-0.4094834	Body; TSS200
HIP1	-0.4080506	Body
IGF1	-0.4076891	TSS200; Body
GIMAP8	-0.4074185	TSS1500
FGR	-0.4069728	Body
STK17B	-0.4063358	TSS1500
ADAP2	-0.4057109	TSS200
SLC16A6	-0.4054715	5'UTR
IL1R2	-0.4051378	1stExon; 5'UTR; TSS200
CIITA	-0.402844	Body
SIAE	-0.4015416	5'UTR; Body; TSS1500; 1stExon
SGMS1	-0.4014815	5'UTR
CCR6	-0.4007177	5'UTR; 1stExon
KLF11	-0.4003994	3'UTR
ZC3H12A	-0.40022616	Body; TSS1500
NFAM1	-0.3998265	3'UTR; Body; TSS200
SORT1	-0.3993694	TSS1500
SOAT1	-0.3992002	TSS1500
HLX	-0.3989063	Body
CTTN	-0.3988483	Body
CDC42EP3	-0.3985891	TSS1500
HLA-DMB	-0.3985718	TSS1500; Body
CYBB	-0.3977865	Body
SLC4A1	-0.3960382	TSS1500
CD44	-0.3958158	Body
FOXO3	-0.3950043	Body; TSS200; 5'UTR
IGSF10	-0.3946555	Body
TFEB	-0.3945108	TSS200; TSS1500
EPB42	-0.3934424	Body; TSS1500
FAM49A	-0.3922532	5'UTR
MTSS1	-0.3921433	Body
TREM1	-0.3913257	TSS1500
SYTL3	-0.3908251	Body

GPR183	-0.389124	5'UTR; Body
LOXL1	-0.3878678	Body
JAZF1	-0.3877991	Body; TS200
RAB31	-0.3864439	Body
LY9	-0.386207	Body
CDA	-0.3858735	TSS1500
CCDC50	-0.3853439	TSS200; Body
E2F2	-0.3846047	Body
GNAI1	-0.3844818	TSS200; Body
HCK	-0.3844469	5'UTR; Body
CD80	-0.3842651	5'UTR
PTPRJ	-0.3839098	TSS1500
ITGAM	-0.3832651	Body
KYNU	-0.3815912	5'UTR
BPGM	-0.3809939	Body
LY86	-0.3808367	TSS1500;Body
RTN1	-0.3807298	TSS1500
CCR7	-0.38012772	5'UTR; 1stExon
RBM38	-0.379814	Body
P2RY13	-0.3797357	Body
FTH1	-0.3786042	Body
IL7R	-0.378413	Body
APP	-0.3782924	5'UTR; Body
ITK	-0.3753772	Body; TS200
IL1R1	-0.3751715	5'UTR
MYBL1	-0.3742029	Body;TSS1500
CDKN1A	-0.3739632	TSS1500; TSS1500
NCEH1	-0.3738486	5'UTR; Body
LILRB1	-0.3736937	1stExon; 5'UTR
DMXL2	-0.3718864	TSS1500
GNG2	-0.3714105	5'UTR
LRIG1	-0.3703783	TSS1500
KLF3	-0.3701541	5'UTR; TSS1500
TM6SF1	-0.3693959	Body; TSS200
GPR124	-0.3687225	Body
EPB49	-0.3679338	TSS1500; 5'UTR
LCK	-0.367693	5'UTR; TSS1500
MICAL2	-0.3675344	5'UTR; Body
HNRNPU	-0.3672348	Body
FAIM3	-0.3670141	5'UTR; TSS1500
EIF2C2	-0.3664859	Body
SLC24A4	-0.3660158	TSS1500; 5'UTR; Body
PTAFR	-0.3656107	TSS1500; 5'UTR
GCNT2	-0.3623836	TSS1500
SGSH	-0.362181	TSS1500; Body
OAS2	-0.3614378	Body
FCER1A	-0.36109896	5'UTR
NFIX	-0.3600177	Body

CKAP4	-0.3598422	Body
FGL2	-0.3597343	1stExon; 5'UTR; Body
CTSB	-0.3588331	TSS1500; 5'UTR
MXD1	-0.3577471	Body
SGMS2	-0.3559443	5'UTR; 3'UTR; Body
CPNE3	-0.3554335	5'UTR
GGA2	-0.3537893	TSS200
KIAA1598	-0.353777	TSS1500
SECTM1	-0.3537652	5'UTR
NR4A1	-0.3536526	Body; 5'UTR
AMICA1	-0.3532758	TSS1500
LONRF2	-0.3531726	Body; TSS1500
FGD4	-0.3528258	5'UTR
ZNF503	-0.3526502	Body
CLEC3B	-0.3517973	TSS1500
SLC7A7	-0.3517074	1stExon; 5'UTR
CD163	-0.3516303	Body
MAP2K3	-0.3508558	Body
SIRPB1	-0.3506107	Body
HBG1	-0.3504848	TSS1500
CCDC149	-0.3502016	5'UTR
NEDD9	-0.3497434	TSS200
AQP1	-0.3496939	Body; TSS200; 5'UTR
STS	-0.3480817	Body
DDIT4	-0.3479907	3'UTR
ARHGEF12	-0.3478758	TSS1500
FHDC1	-0.3470921	Body; TSS1500
FAM65C	-0.3466354	Body
RNF144B	-0.3462124	Body
PLAUR	-0.3445905	TSS1500
EIF1AY	-0.3445179	TSS200
CREM	-0.3439662	5'UTR
TRIB1	-0.3422189	Body; 5'UTR
CCR2	-0.3410734	TSS1500
PLA2G16	-0.3407739	Body
CD74	-0.340652	TSS1500
CLEC7A	-0.3401647	TSS1500
MMP19	-0.3399606	3'UTR
RAB7L1	-0.33995927	1stExon; 5'UTR
FAM83A	-0.3397714	1stExon; 5'UTR
PMP22	-0.3394877	TSS1500; 5'UTR
OLFML2A	-0.3389963	TSS1500; Body
LOC200772	-0.3384061	TSS200
P2RY14	-0.3381514	Body; 5'UTR
WDFY3	-0.3378837	TSS1500; Body
RBM47	-0.3373566	5'UTR
SEC14L1	-0.3358908	Body
MRPL43	-0.3358361	Body; 3'UTR

SPTLC2	-0.3355075	Body
RBPMS	-0.3341432	Body
THBS1	-0.3338226	TSS1500
C1orf56	-0.3335805	1stExon
IL1RN	-0.3324023	5'UTR;Body;Body
ARTN	-0.3324015	ExonBnd; 5'UTR
CCL5	-0.3323543	1stExon; 5'UTR
ADAMTSL4	-0.3320604	5'UTR;5'UTR
NRGN	-0.3317802	Body
HCP5	-0.3312686	3'UTR
LEF1	-0.3309254	Body
GIMAP4	-0.3301855	TSS1500
SP140	-0.3297458	Body
CD4	-0.3289801	TSS200
ARL4C	-0.3286406	1stExon
ID1	-0.32830176	1stExon
OAZ3	-0.3279758	TSS200; Body
TNNT1	-0.3272874	3'UTR
PHLDA1	-0.3271443	3'UTR
CX3CR1	-0.3269341	Body; 5'UTR
MPP7	-0.3260074	Body
FOXF1	-0.325404	TSS1500
LILRB4	-0.3252312	TSS1500
NCF2	-0.3250809	Body
PPAP2B	-0.3250181	Body
G0S2	-0.324448	TSS1500
VIM	-0.3235573	Body
RIN2	-0.3234372	Body; TSS1500
SERPINB6	-0.3198813	5'UTR; 1stExon; TSS1500
CDH1	-0.3193179	Body
RASSF6	-0.3186354	5'UTR; Body
CDH2	-0.3183975	TSS1500; Body
SH3BP5	-0.31830303	Body; 5'UTR
ADAM8	-0.3182273	Body
MARCKS	-0.31787906	TSS1500
NPL	-0.3177076	Body
CLU	-0.3170251	TSS200; Body
MS4A4A	-0.3168389	5'UTR;Body
NPTX2	-0.3166648	Body
BLNK	-0.3165006	Body
C6orf192	-0.3161532	Body
CH25H	-0.3153188	TSS1500
STAB1	-0.3148122	3'UTR
KLHL2	-0.3143506	Body; TSS1500
RXFP1	-0.3139587	5'UTR; Body
ZNF467	-0.313808	TSS200
LONRF2	-0.3137	TSS1500
C12orf59	-0.31238063	Body

FGL2	-0.31118155	Body; TSS200
FGD2	-0.31080545	Body
SLFN13	-0.3106282	TSS1500
CHRDL1	-0.3096038	5'UTR; Body
LYST	-0.3092074	Body; 5'UTR
C1orf162	-0.3076488	Body
MAFB	-0.307111	TSS1500
CDC42BPB	-0.3065188	Body
MDM2	-0.3046978	Body
SESN3	-0.3046702	TSS200; Body; 5'UTR
FCGRT	-0.3046685	5'UTR; TSS200
C11orf9	-0.3039539	TSS1500
GLIPR2	-0.3033509	Body
CREB5	-0.3026936	TSS1500
LDLR	-0.3025171	Body
ATAD2B	-0.3024738	Body
PAX6	-0.3014565	Body
NEURL1B	-0.3014342	3'UTR
NLRP12	-0.3013897	Body
VNN1	-0.3010801	TSS200
