# Supplementary material for: DNMT3A mutants provide proliferating advantage with augmentation of self-renewal activity in the pathogenesis of AML in KMT2A-PTD-positive leukemic cells
Source: Oncogenesis. 2020 Feb 3;9(2):7. doi: 10.1038/s41389-020-0191-6 (PMC6997180; doi:10.1038/s41389-020-0191-6)
Supplement: Supplementary file 17 — Dataset S4 [file 41389_2020_191_MOESM17_ESM.pdf]

(a) List of differentially methylated (differential β-value >0.3) genes in DNMT3A-R882C-expressing EOL-1 cells compared to DNMT3A-WT-expressing EOL-1 cells, which were upregulated (>2 folds) in KMT2A-PTD/DNMT3A-MT AML cells compared to KMT2A-PTD/DNMT3A-WT cells

Gene Symbol	β-value difference	UCSC_REFGENE_GROUP
CD109	0.6125317	TSS1500;Body
ZNF185	0.4908035	Body
PLEKHA1	0.4513276	5'UTR
POSTN	0.44994791	TSS1500
SULF2	0.4342636	Body
FBP1	0.43024017	1stExon;5'UTR
PRKCA	0.4264034	TSS1500
REEP5	0.4195737	TSS1500
GZMK	0.4123287	TSS200
DAAM1	0.40863776	5'UTR
FHL1	0.4080973	TSS1500
BCL6	0.38938945	5'UTR
ALDH1A1	0.3879484	Bod; TSS1500
IL15	0.3828523	5'UTR;Body
PTPRN2	0.3819769	Body
TRIM10	0.3739218	3'UTR;Body
CH25H	0.3691504	TSS200
SOAT1	0.3647139	TSS200
CAMK2D	0.36069366	1stExon;5'UTR
RTN1	0.3562632	Body;TSS1500
SLC25A39	0.3554849	5'UTR
ARL4C	0.3533147	1stExon
RNFT2	0.3526455	Body
VCAN	0.3483615	1stExon;5'UTR
NFKBIZ	0.3450187	3'UTR;TSS1500
SYCP2L	0.3437252	Body
C6orf192	0.3436309	5'UTR;1stExon
OAS2	0.3353315	3'UTR;Body
IRS2	0.3303996	TSS1500
SOX6	0.3301905	Body
RBM6	0.32934711	5'UTR
MYH10	0.3270482	TSS1500
MPPED2	0.3229321	Body
SV2B	0.3221993	5'UTR
ABLIM1	0.3220434	5'UTR;Body
ADCY9	0.3211843	5'UTR
GABBR1	0.3164673	Body
MPP7	0.3147897	5'UTR
IL13RA1	0.3146417	Body
POU2AF1	0.3141886	TSS200
ARHGEF12	0.3107595	Body

CACNA2D3	0.3106442	Body
ZC3H12A	0.3056309	TSS1500
CREB5	0.3051882	5'UTR;Body
WNK1	0.30480489	1stExon
PHLDA2	0.3014534	TSS1500
EIF1AY	0.3009613	3'UTR
RORA	0.3007747	Body
GLI2	0.3007714	Body

(b) List of differentially methylated genes (differential β-value both >0.3 and <-0.3) in the different genomic region of DNMT3A-R882C-expressing EOL-1 cells compared to DNMT3A-WT-expressing EOL-1 cells, which were upregulated (>2 folds) in KMT2A-PTD/DNMT3A-MT AML cells compared to KMT2A-PTD/DNMT3A-WT cells

Gene Symbol	β-value difference	UCSC_REFGENE _GROUP	β-value difference	UCSC_REFGENE _GROUP
ABLIM1	-0.4823841	Body; TSS1500	0.3220434	5'UTR;Body
ARHGEF12	-0.3478758	TSS1500	0.3107595	Body
ARL4C	-0.3286406	1stExon	0.3533147	1stExon
BCL6	-0.6143837	TSS1500	0.38938945	5'UTR
C6orf192	-0.3161532	Body	0.3436309	Body
CACNA2D3	-0.4157644	TSS1500; Body	0.3106442	Body
CAMK2D	-0.5139878	Body	0.36069366	1stExon;5'UTR
CD109	-0.5182999	Body	0.6125317	TSS1500;Body
DAAM1	-0.5159518	5'UTR. Body	0.40863776	5'UTR
EIF1AY	-0.3445179	TSS200	0.3009613	3'UTR
FHL1	-0.6945275	Body; 5'UTR	0.4080973	TSS1500
GABBR1	-0.415323	Body, TSS1500	0.3164673	Body
GLI2	-0.5131778	Body	0.3007714	Body
GZMK	-0.6018102	Body; TSS1500	0.4123287	TSS200
MPP7	-0.3260074	Body	0.3147897	5'UTR
MYH10	-0.4745814	Body	0.3270482	TSS1500
OAS2	-0.3614378	Body	0.3353315	3'UTR;Body
PHLDA2	-0.5724622	5'UTR; 1stExon	0.3014534	TSS1500
PRKCA	-0.5096307	Body	0.4264034	TSS1500
PTPRN2	-0.5910232	Body	0.3819769	Body
RORA	-0.4721302	Body	0.3007747	Body
RTN1	-0.3807298	TSS1500	0.3562632	Body;TSS1500
SOAT1	-0.3992002	TSS1500	0.3647139	TSS200
SOX6	-0.4997559	Body; 5'UTR; TSS1500	0.3301905	Body
SULF2	-0.4759795	Body	0.4342636	Body
SV2B	-0.5050368	Body; 5'UTR	0.3221993	5'UTR
TRIM10	-0.4100815	Body	0.3739218	3'UTR;Body
ZC3H12A	-0.40022616	Body; TSS1500	0.3056309	TSS1500
ZNF185	-0.4737663	Body	0.4908035	Body
