# Supplementary material for: DNMT3A mutants provide proliferating advantage with augmentation of self-renewal activity in the pathogenesis of AML in KMT2A-PTD-positive leukemic cells
Source: Oncogenesis. 2020 Feb 3;9(2):7. doi: 10.1038/s41389-020-0191-6 (PMC6997180; doi:10.1038/s41389-020-0191-6)
Supplement: Supplementary file 18 — Dataset S5 [file 41389_2020_191_MOESM18_ESM.pdf]

List of hypomethylated (β-value <0.25) genes in DNMT3A-R882C-expressing EOL-1 cells compared to DNMT3A-WT-expressing EOL-1 cells, which were upregulated (>2 folds) in KMT2A-PTD/DNMT3A-MT AML cells compared to KMT2A-PTD/DNMT3A-WT cells

Gene Symbol	DNMT3A-WT β-value (>0.4)	DNMT3A-R882C β-value (<0.25)	UCSC_REFGENE_GROUP
ABLIM1	0.6111315	0.1287474	Body;TSS1500;1stExon;5'UTR
ACSL1	0.7864226	0.1866849	TSS1500;5'UTR;5'UTR
ADAP2	0.5969528	0.1912419	TSS200
AFF3	0.5876899	0.1127702	Body
AGPAT9	0.646695	0.1464965	TSS1500;TSS200;Body
AKAP13	0.4729445	0.0979597	5'UTR;Body
AKT3	0.5903663	0.1257104	Body
ALOX5	0.7571095	0.1961322	Body
ALOX5AP	0.6191711	0.1052164	TSS1500;Body
AMICA1	0.4790553	0.1582269	Body;TSS200;5'UTR
APP	0.6168313	0.2385389	5'UTR;Body
ARHGEF12	0.8527059	0.1838934	Body;5'UTR
ASAP1	0.5913829	0.1300012	Body
ATAD2B	0.400523	0.1920222	Body
BACH2	0.7286569	0.2256455	5'UTR;Body
BAG3	0.5663179	0.1536337	Body;TSS1500
BASP1	0.6653357	0.2310405	Body;TSS1500
BCL11B	0.6353362	0.1895124	TSS200;Body
BCL6	0.7672022	0.1528185	TSS1500
BHLHE40	0.7667215	0.1044437	Body
BPGM	0.5063702	0.1253763	Body
BTBD19	0.4273343	0.1882442	TSS1500
C10orf54	0.8634833	0.11863	TSS1500;Body
C11orf9	0.4630885	0.1696132	Body
C17orf91	0.8055875	0.2322541	TSS1500;Body
C1orf38	0.6497338	0.2402504	Body;TSS200
C6orf192	0.4324945	0.1163413	Body
C9orf72	0.4452483	0.1888233	TSS1500
CACNA2D3	0.6915024	0.2095766	Body;TSS200;5'UTR;TSS1500
CAMK2D	0.5392638	0.1932873	3'UTR;Body
CCDC149	0.5507274	0.2450846	TSS1500;5'UTR
CCDC50	0.5518672	0.2028667	TSS200;5'UTR;1stExon;Body
CCL5	0.4994499	0.1670956	1stExon;5'UTR
CCR2	0.5189702	0.1325785	3'UTR;TSS200
CCR7	0.4734981	0.09337038	5'UTR;1stExon
CD109	0.7151116	0.1968117	Body
CD44	0.52953	0.1337142	Body
CD48	0.4256742	0.1816362	TSS1500
CD86	0.6963372	0.219607	5'UTR;Body;1stExon
CDA	0.6305026	0.2446291	TSS1500;Body
CDC42EP3	0.4719882	0.1929881	5'UTR
CDH2	0.5250155	0.2458024	ExonBnd;Body
CDKN1A	0.4787558	0.1376642	TSS1500
CELF1	0.5325876	0.1036305	1stExon;5'UTR

CHST15	0.4615681	0.2154131	Body
CLCN5	0.743822	0.1994958	Body
CLEC2D	0.5437726	0.2218428	Body
CLU	0.5075555	0.194682	TSS200;Body
CMTM4	0.4599714	0.2182803	TSS1500
CREB5	0.6262407	0.1579986	5'UTR
CRISPLD2	0.8671889	0.1331451	5'UTR
CSGALNACT1	0.6627461	0.1734484	Body;5'UTR
CTSB	0.4943563	0.170621	5'UTR
CTSZ	0.6790727	0.2449974	Body
CTTN	0.5346184	0.1640795	Body
CXCR4	0.6658582	0.2345719	3'UTR;1stExon;3'UTR
CYBB	0.5701232	0.1723367	Body
CYorf15A	0.6339779	0.1450476	TSS1500;1stExon;5'UTR
CYTIP	0.4062312	0.1438899	TSS1500
DAAM1	0.762267	0.2463152	5'UTR;Body
DLG5	0.5515322	0.2084315	Body
DMXL2	0.5357864	0.1639	TSS1500
DNAJA4	0.6952356	0.2186747	TSS200;Body
DNTT	0.5395293	0.1675722	Body
DOCK10	0.796639	0.2342235	Body
DOK2	0.5156754	0.2325113	Body
EIF2C2	0.5773162	0.2475445	Body
EMP1	0.7875094	0.1504962	TSS1500;5'UTR
ENTPD1	0.6153943	0.1834285	5'UTR;Body;
EPB41	0.6593991	0.2349202	5'UTR
EPB41L3	0.7976951	0.2261582	Body
EPB42	0.5345967	0.1787282	Body
EPB49	0.4381279	0.226514	TSS1500
EPS8	0.523399	0.1115237	TSS200;TSS1500
FAM107B	0.706801	0.1483991	Body;5'UTR
FAM124A	0.55549	0.1310157	Body
FAM13A	0.4348371	0.124888	Body
FAM198B	0.8025237	0.1857157	Body
FAM49A	0.5490013	0.1567481	5'UTR
FAM65C	0.5243077	0.2046524	5'UTR;Body
FCER1A	0.4505766	0.08947764	5'UTR;Body
FCGRT	0.5031478	0.1984793	5'UTR;TSS200
FERMT1	0.5354626	0.1416152	Body
FGD4	0.5223092	0.1694834	5'UTR
FGL2	0.4628832	0.1031489	1stExon;5'UTR;Body;TSS200
FGR	0.5966402	0.1896674	Body
FHL1	0.8806153	0.1860878	Body;5'UTR
FLVCR2	0.8121551	0.2362509	1stExon
FNIP2	0.6058655	0.1045345	3'UTR;Body
FOS	0.4317363	0.1897441	Body
FRAT1	0.6658452	0.2441418	TSS1500
FRMD3	0.870102	0.1452273	TSS1500;Body
FRMD4A	0.6828222	0.2336487	TSS1500;Body
FTH1	0.4846087	0.1060045	Body

G0S2	0.5646282	0.2401802	TSS1500
GABBR1	0.448646	0.2332671	TSS1500;Body
GCNT2	0.5143322	0.1686167	Body
GGA2	0.5462515	0.1924622	TSS200
GLI2	0.5378314	0.1050164	Body
GLIPR2	0.4418921	0.1385412	Body
GOLGA8B	0.4114852	0.216333	Body
GPR183	0.6642824	0.1443176	TSS1500;Body;5'UTR
GZMA	0.7097123	0.2189359	TSS1500
GZMH	0.5022259	0.09095489	Body
GZMK	0.7648544	0.1630442	Body;TSS1500
H2AFY	0.5221581	0.2369775	TSS1500;TSS200
HCK	0.5138176	0.2221029	TSS1500;5'UTR;Body
HEMGN	0.7398857	0.1537188	Body
HEPACAM2	0.4215817	0.2003385	TSS1500;Body
HIP1	0.6021605	0.1941099	Body
HK1	0.4535467	0.1264971	Body
HK3	0.6860662	0.2346494	TSS200
HLA-DMB	0.4552509	0.237738	1stExon;5'UTR;Body
HLA-DPA1	0.71842	0.2235188	Body
HLA-DPB1	0.4943464	0.2356359	Body
HLA-DRA	0.6220057	0.2118302	TSS1500
HLA-DRB1	0.6758301	0.2265878	3'UTR
HLX	0.5238761	0.1249698	Body
HNRNPU	0.5876265	0.2203917	Body
ID1	0.4091833	0.08088154	1stExon
IGF1	0.4170577	0.1008382	Body;TSS1500
IGF2R	0.5542388	0.164649	Body
IL1R1	0.6242039	0.2490324	5'UTR
IL1R2	0.5271727	0.1681784	TSS200;1stExon;5'UTR
IQSEC1	0.7183223	0.1084933	TSS1500;Body
IRF8	0.4541445	0.07402007	TSS1500
ITK	0.5792623	0.2038851	Body;TSS200;TSS1500
JAZF1	0.497488	0.1100798	Body;TSS200
KAT2B	0.6631923	0.1697227	Body
KCTD12	0.7506894	0.2227449	TSS1500
KIAA1598	0.4817513	0.2252491	TSS1500
KLF3	0.6004699	0.2303158	TSS1500;5'UTR
KLF9	0.8447675	0.2234284	Body
KMO	0.7034646	0.2452505	Body
KYNU	0.6109754	0.2293842	5'UTR
LAPTM5	0.4041839	0.1177967	Body
LATS2	0.5677074	0.1218864	TSS1500;5'UTR
LDLR	0.4439834	0.1479336	Body
LDLRAD3	0.643079	0.09644096	5'UTR;Body
LEF1	0.4678631	0.1369377	Body
LOC150381	0.5899045	0.1604039	Body;TSS1500
LOC200772	0.5395477	0.2365537	TSS1500
LOXL1	0.5689698	0.2372352	Body
LRIG1	0.4367895	0.1589565	Body

LRRFIP1	0.6109995	0.2348645	Body
LRRK2	0.8151468	0.2140703	Body
LSP1	0.7548262	0.2372137	5'UTR;1stExon;Body;TSS200
LY9	0.5154005	0.1291935	Body
LYN	0.554941	0.07940845	TSS1500;Body
LYST	0.4970591	0.2102646	1stExon;Body;5'UTR
MAFF	0.6811712	0.1588493	5'UTR
MAML2	0.5378467	0.2193962	Body
MAP2K3	0.5574429	0.2065871	Body
MARCH1	0.480783	0.14157	Body
MARCKS	0.4064782	0.08859914	TSS1500
MCL1	0.5969227	0.1530097	TSS1500
MCTP1	0.4583155	0.1769619	TSS1500;Body
MDM2	0.5310652	0.2263674	Body
MEF2C	0.614737	0.1982118	Body
MEIS1	0.5661173	0.1163229	3'UTR
METRNL	0.6227032	0.234173	Body
METTL7B	0.4955159	0.2140837	1stExon;5'UTR
MFHAS1	0.6821117	0.1367097	Body
MGLL	0.5249072	0.2212167	Body
MICAL2	0.4912432	0.1237088	5'UTR;Body
MLLT4	0.6618008	0.09541469	5'UTR;Body
MMRN1	0.4245221	0.2168145	Body
MRC1	0.4541021	0.1560752	Body
MS4A6A	0.6588762	0.1993822	TSS1500
MTSS1	0.4922317	0.1652969	Body
MX1	0.5874563	0.170107	Body
MYBL1	0.4661919	0.1645559	TSS1500
MYL4	0.4979782	0.2220596	Body
NACC2	0.5601272	0.1359164	5'UTR
NFAM1	0.6070086	0.2147162	Body;TSS200;3'UTR
NID1	0.4658277	0.2093201	Body
NLRP1	0.570488	0.2440642	Body;1stExon;5'UTR
NLRP3	0.660673	0.1965909	TSS1500;TSS1500
NOD2	0.6124679	0.1666339	TSS1500
NR4A1	0.4817209	0.1516088	Body;TSS1500
OAS2	0.5357651	0.1743273	Body
OPTN	0.4720755	0.2342613	5'UTR
OSBP2	0.4458734	0.2163553	Body
P2RY13	0.5853816	0.2056459	Body
PADI4	0.5381649	0.09287025	Body
PCNX	0.5167426	0.1226321	TSS1500;Body
PDE4B	0.6587439	0.1764447	TSS1500;Body
PDZK1IP1	0.511849	0.09659113	Body
PIK3R5	0.4193839	0.1323814	5'UTR
PILRA	0.8313374	0.1848829	TSS1500;TSS200;Body
PKIB	0.503436	0.2410547	TSS1500;5'UTR
PLCL2	0.7831241	0.2167391	Body
PPAP2B	0.4566197	0.1896268	Body
PRDX2	0.4165202	0.2231473	TSS1500

PRKAR2B	0.6003155	0.1053785	Body
PRKCA	0.730038	0.2204073	Body
PSAP	0.6526313	0.1960568	TSS1500
PSTPIP1	0.7656977	0.207997	Body
PTAFR	0.5039145	0.1383038	TSS1500;5'UTR;Body
PTPRC	0.6561763	0.1870697	Body
PTPRE	0.7560015	0.2181123	5'UTR;Body;TSS1500
PTPRJ	0.629119	0.2466728	Body
PTPRN2	0.7089328	0.1179096	Body
PTRF	0.6020005	0.2294237	Body
RAB20	0.8384151	0.1676263	Body
RAB31	0.4910504	0.1046065	Body
RAP1GAP	0.7798095	0.2433792	Body;5'UTR
RASGEF1B	0.6122789	0.1960491	TSS1500
RASGRP4	0.4533592	0.2046587	Body
RASSF4	0.4614336	0.1370048	TSS200;Body
RBM47	0.5088573	0.2194167	5'UTR
REEP5	0.4397507	0.1940157	TSS1500
RELL1	0.7090983	0.2067748	Body
RGS2	0.5934973	0.1039926	TSS1500
RHCE	0.7315207	0.2132268	Body
RNASE4	0.4635774	0.1925793	5'UTR
RNFT2	0.4634969	0.2205407	Body
RORA	0.6092895	0.1371593	Body
RTN1	0.5202156	0.1394858	TSS1500;Body
RXRA	0.544114	0.08023899	Body
S100A8	0.8290078	0.2236361	5'UTR;TSS200
S1PR3	0.4786995	0.2320424	Body;1stExon;5'UTR
SEC14L1	0.4746867	0.1387959	Body
SGMS1	0.5398133	0.2403387	5'UTR
SGMS2	0.5788477	0.2229034	5'UTR;Body
SGSH	0.5138456	0.1516646	TSS1500;Body
SH2B3	0.5208831	0.1777752	TSS200;Body
SH3BP5	0.4026373	0.08433427	Body;5'UTR
SIAE	0.5152772	0.1137356	5'UTR;Body;TSS1500
SIRPB1	0.4554967	0.104886	Body
SLA	0.8093455	0.2466155	TSS1500;Body;5'UTR
SLC15A3	0.411242	0.1444066	Body
SLC16A6	0.5921295	0.2337108	5'UTR
SLC24A4	0.6135894	0.2475736	TSS1500;5'UTR;Body
SLC2A14	0.4599469	0.1700796	Body
SLC2A6	0.6385185	0.2128464	Body;1stExon;5'UTR
SLC8A1	0.5075589	0.2163268	TSS1500;5'UTR;Body
SORT1	0.509473	0.1101036	TSS1500;Body;5'UTR
SOX6	0.5515471	0.1204822	5'UTR;TSS1500;Body
SPATA13	0.6455416	0.157927	Body;TSS200;5'UTR;Body
SRD5A1	0.5001769	0.2089388	TSS1500;Body
SSH2	0.5522372	0.1006535	TSS1500;Body
SSH2	0.7648937	0.2284545	Body
STK17B	0.4572664	0.1024357	TSS1500

STOX2	0.4118459	0.1504764	TSS200
SULF2	0.4614516	0.1871425	Body
SV2B	0.6158153	0.1107785	Body;5'UTR
SVIL	0.7038605	0.1731791	5'UTR;Body
SYCP2L	0.4896611	0.208303	Body
SYNJ2	0.6989185	0.219579	Body;TSS200
TBC1D8	0.7301997	0.2424037	Body
TBL1X	0.6391248	0.1142443	5'UTR
TGFBI	0.68317	0.2275282	TSS1500
TLR5	0.6905285	0.1686722	5'UTR
TMCC2	0.4654757	0.1483985	5'UTR;TSS1500;Body
TMEM49	0.4108082	0.1601317	Body
TNS1	0.5725815	0.2176868	5'UTR;ExonBnd;Body;TSS200;5'UTR
TOB1	0.4600603	0.207775	Body;TSS1500
TREM1	0.4078784	0.1633388	TSS1500
TSC22D3	0.7067406	0.07934981	5'UTR;Body;1stExon
TXNIP	0.7208661	0.2367023	TSS1500;Body
UBE2D1	0.8328801	0.1905372	5'UTR;Body
UNC13B	0.6389117	0.1559306	Body
VAV2	0.4934168	0.207177	Body
VIM	0.467902	0.1443447	Body
VNN1	0.4256141	0.124534	TSS200
VNN2	0.4618669	0.2275781	Body
WDFY3	0.4642869	0.1264032	TSS1500;Body
WIPI1	0.4435276	0.1338905	Body;5'UTR
WNK1	0.4440389	0.2063823	Body
YPEL4	0.4321942	0.2238027	5'UTR
ZAK	0.6943122	0.2374947	TSS1500;Body
ZC3H12A	0.5691403	0.2439046	TSS1500;Body
ZCCHC6	0.4055097	0.1847199	Body
ZFHX3	0.8841931	0.2353442	Body;5'UTR
ZNF467	0.4487167	0.1349087	TSS200;1stExon;5'UTR
